# A Stimuli-Responsive Rotaxane–Gold Catalyst: Regulation of Activity and Diastereoselectivity

**DOI:** 10.1002/anie.201505464

**Published:** 2015-09-21

**Authors:** Marzia Galli, James E M Lewis, Stephen M Goldup

**Affiliations:** School of Chemistry, University of Southampton Highfield, Southampton, SO17 1BJ (UK)

**Keywords:** catalysts, gold, molecular machines, rotaxanes, supramolecular chemistry

## Abstract

A rotaxane-based Au catalyst was developed and the effect of the mechanical bond on its behavior was studied. Unlike the non-interlocked thread, the rotaxane requires a catalytically innocent cofactor, the identity of which significantly influences both the yield and diastereoselectivity of the reaction. Under optimized conditions, Au^I^ (the catalyst), Ag^I^ (to abstract the Cl^−^ ligand), and Cu^I^ (the cofactor) combine to produce a catalyst with excellent activity and selectivity.

Catalysts based on interlocked molecules have recently begun to receive increased attention.^1^ The majority of the systems reported take advantage of the well-studied ability of the mechanically bonded components to undergo large-amplitude relative motion,^2^ including examples in which this motion alters the reaction chemoselectivity,^3^ machines inspired by DNA polymerase that employ an interlocked catalyst–substrate architecture to produce highly processive reactions,^4^ and systems where the catalytic activity of the rotaxane is controlled by reversible shielding of organocatalytic moieties in the thread.^5^–^7^

In contrast to these applications of mechanical motion, comparatively little is known about the influence of the mechanical bond itself on the outcome of catalyzed reactions.^8^ In 2004, Takata and co-workers reported that an achiral imidazolium organocatalyst encircled by a chiral macrocycle mediates an enantioselective benzoin reaction, albeit with moderate *ee*.^9^ Very recently, Leigh and co-workers reported a chiral [2]rotaxane ligand for Ni that exhibits higher enantioselectivity than a non-interlocked model complex, although at the cost of reduced activity owing to steric hindrance.^10^ These results suggest that the sterically crowded environment provided by the mechanical bond could be used to engineer novel reaction fields to alter the stereoselectivity of catalysts that are hard to control with conventional scaffolds.

Although synthetically powerful, gold(I)-mediated reactions are perhaps the quintessential example of an activation mode for which it is hard to engineer the ligand to sterically influence the reaction because of the linear coordination geometry of Au^I^.^11^ Herein, we report a rotaxane–gold catalyst^12^, ^13^ in which the mechanical bond influences both diastereoselectivity and catalytic activity, and we demonstrate stimuli-responsive control of both of these important reaction parameters.

Rotaxane–gold complex [**4**AuCl] was synthesized^14^ in excellent yield over three steps from macrocycle **1**, alkyne **2**, and azide **3** by using our small-macrocycle modification^15^ of Leigh’s active template Cu-mediated alkyne–azide cycloaddition^16^ (AT-CuAAC) reaction,^17^ followed by reduction of the phosphine oxide moiety and formation of the Au complex (Scheme 1). Non-interlocked complex [**5**AuCl] was synthesized in an analogous manner.

**scheme 1:**
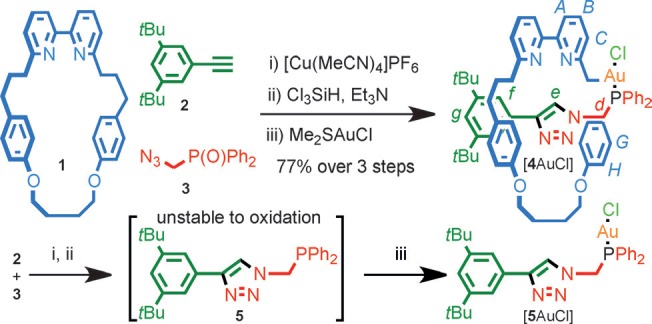
Synthesis of [4AuCl] and [5AuCl]. Reagents and Conditions: i) [Cu(MeCN)_4_]PF_6_, *i*Pr_2_EtN, EtOH, 80 °C, 18 h; ii) Cl_3_SiH, Et_3_N, PhMe/CH_2_Cl_2_ (6:1), 100 °C, 18 h; iii) Me_2_SAuCl, CH_2_Cl_2_, RT, 1 h.

During the synthesis of **4** and **5**, the first significant effect of the mechanical bond became apparent: although phosphine rotaxane **4** does not require special handling, non-interlocked **5** is extremely susceptible to oxidation and reverts to the corresponding phosphine oxide on standing in CDCl_3_. Thus, the mechanical bond appears to stabilize the relatively electron-rich alkyl phosphine moiety.^8^ Single crystals of [**4**AuCl] suitable for X-ray analysis were grown by slow evaporation from CDCl_3_ (Figure 1).^18^ The space-filling representation of [**4**AuCl] clearly demonstrates the sterically hindered environment around the Au center provided by the mechanical bond. In the solid state, triazole proton H_*e*_ and one of methylene protons H_*d*_ participate in bifurcated C–H⋅⋅⋅N hydrogen bonds with the pyridine nitrogen atoms.

**Figure 1:**
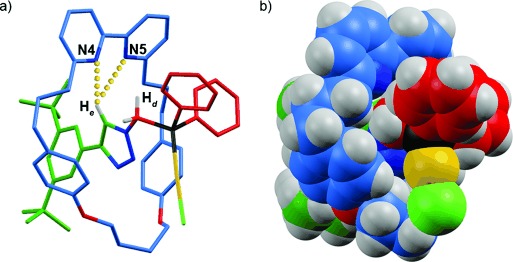
Single-crystal X-ray structure of [4AuCl] as a) a capped-stick model with the shorter of the N⋅⋅⋅H contacts indicated; b) a space-filling representation. ^1^H labelling as in Scheme 1. Selected interatomic distances: N4–H_*d*_=2.7 Å; N5–H_*d*_=3.1 Å; N4–H_*e*_=2.9 Å; N5–H_*e*_=2.7 Å; P–Au=2.2 Å; Au–Cl=2.3 Å; angle P-Au-Cl=176°.

Despite the unusual nature of the Au coordination environment in [**4**AuCl], the ^31^P NMR spectra of the thread and rotaxane–AuCl complexes are similar (Figure 2), exhibiting single resonances at 26.2 and 29.2 ppm, respectively. In contrast, their ^1^H NMR spectra are very different; as well as the expected shielding of thread resonances (e.g. H_*d*_, H_*f*_ and H_*g*_), triazole proton H_*e*_ resonates at 9.5 ppm in the rotaxane–Au complex, which is 1.7 ppm higher than in [**5**AuCl], thus suggesting that the C–H⋅⋅⋅N hydrogen bond between H_*e*_ and the bipyridine nitrogen atom observed in the solid state is at least partially maintained in solution.

**Figure 2:**
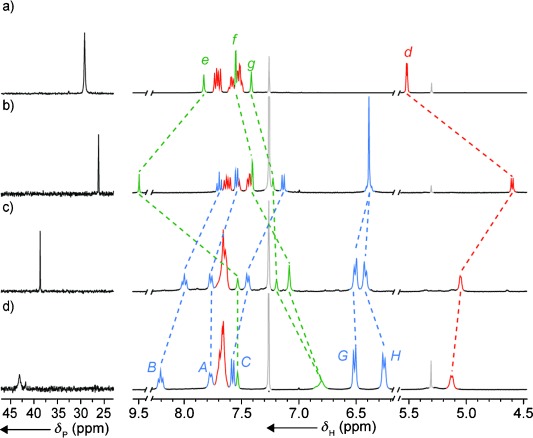
Partial ^31^P (162 MHz) and ^1^H (400 MHz) NMR (CDCl_3_, 300 K) spectra for a) [5AuCl], b) [4AuCl], c) [4AuCl]+AgSbF_6_, and d) [4AuCl]+[Cu(MeCN)_4_]PF_6_+AgSbF_6_. Selected peaks are assigned with labelling as shown in Scheme 1. Solvent peaks are shown in dark grey.

To investigate the effect of the mechanical bond on the catalytic behavior of [**4**AuCl], we selected Toste’s Au^I^-mediated modification of the Ohe–Uemura cyclopropanation reaction as a simple, well-understood model system.^19^, ^20^ As in many Au^I^-mediated reactions, the active catalyst is proposed to be an LAu^+^ π acid, typically generated by abstraction of the Cl ligand from LAuCl by Ag^I^ salts.^11^ ^31^P NMR analysis of the product of treating [**4**AuCl] with AgSbF_6_ (Figure 2 c) revealed a new resonance at higher chemical shift (38.7 ppm), which is consistent with Cl abstraction and formation of a solvated PAu^+^ complex.^21^ However, in the presence of AgSbF_6_, [**4**AuCl] failed to mediate the reaction between propargylic ester **6** and styrene (**7**) to produce cyclopropanes **8** (Table 1, entry 1). Despite the lack of activity of [**4**AuCl], in situ NMR analysis of reactions containing [**4**AuCl] confirmed the presence of [**4**Au]^+^ under the reaction conditions. In contrast, non-interlocked complex [**5**AuCl] (entry 2) successfully produced **8** with moderate selectivity in good yield.

**Table 1:**
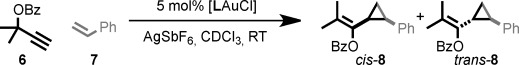
Effect of additives on the catalytic activity of [4AuCl] and [5AuCl].

Entry	[LAuCl]	Additive	Yield^[a]^ (*cis*/*trans*^[b]^)
1	[**4**AuCl]	–	n.r.
2	[**5**AuCl]	–	85 % (10:1)
3	[**4**AuCl]	TsOH	62 % (15:1)
4	[**4**AuCl]	[Cu(MeCN)_4_]PF_6_	93 % (16:1)
5	[**4**AuCl]	Zn(OTf)_2_	76 % (13:1)
6	[**4**AuCl]	Cd(OTf)_2_	58 % (11:1)
7	[**5**AuCl]	Zn(OTf)_2_	86 % (10:1)
8	[**5**AuCl]	[Cu(MeCN)_4_]PF_6_	68 % (9:1)
9	–	H^+^, Cu^I^, Zn^II^ or Cd^II^	n.r.

[a] Determined by ^1^H NMR with Cl_2_CHCHCl_2_ as internal standard. n.r.=no reaction. [b] Determined by HPLC analysis.

Comparison of the ^1^H NMR spectra of [**4**AuCl] and [**4**Au]^+^ provided a clue to the origin of the lack of activity of the rotaxane catalyst: upon abstraction of the Cl anion, the resonance corresponding to triazole proton H_*e*_ shifts from 9.5 to 7.5 ppm, thus suggesting that the C–H⋅⋅⋅N interaction present in [**4**AuCl] is at least partially interrupted in [**4**Au]^+^. This observation is consistent with the Au^+^ center interacting with the N donor atoms of the macrocycle, thereby interrupting the weaker hydrogen-bonding interaction. The proposed N–Au interaction would be expected to temper the catalytic activity of [**4**Au]^+^ by reducing the π-acidity of the metal center and preventing coordination of the substrate.^22^ Based on this hypothesis, a solution suggested itself: the addition of a guest that coordinates in the macrocyclic cavity and is able to effectively compete with the N–Au interaction should remove the inhibition and switch the catalyst “on”.^23^

Addition of TsOH, [Cu(MeCN)_4_]PF_6_, Zn(OTf)_2_, or Cd(OTf)_2_ to a solution of [**4**AuCl] in CDCl_3_ led to dramatic changes in the ^1^H NMR spectra but minimal change in the ^31^P resonance (∂_P_=24.5, 23.9, 27.7, and 24.4 ppm respectively; see the Supporting Information for details), thus suggesting that the P–Au bond is unaffected by guest binding. Addition of AgSbF_6_ to [**4**AuCl] and [Cu(MeCN)_4_]PF_6_ in CDCl_3_ resulted in the formation of a complex with a broad ^31^P resonance consistent with the desired PAu^+^ species (Figure 2 d). Furthermore, in the presence of 1 equiv of TsOH (Table 1, entry 3), [**4**AuCl] produced cyclopropanes **8** in reasonable yield in just 1 h. More effective still, both Cu^I^ and Zn^II^ (entries 4 and 5 respectively) resulted in rapid reactions and excellent yields of **8**. Replacing Zn(OTf)_2_ with Cd(OTf)_2_ (entry 6) led to a diminished yield. In contrast to the behavior of the rotaxane, reactions mediated by [**5**AuCl] were unaffected by the presence of Zn^II^ (entry 7), while addition of Cu^I^ (entry 8) led to partial decomposition of the catalyst and a diminished yield of **8**.^24^ In the absence of Au^I^, the additives have no intrinsic catalytic behavior (entry 9).

The effect of guest binding on the catalytic behavior of [**4**AuCl] is noteworthy on a number of counts. Firstly, and most obviously, the activity of the catalyst is strongly dependent on the presence of the guest. Building on these results, we performed in situ switching experiments with [**4**AuCl] and the best performing guests, Cu^I^ and Zn^II^ (Scheme 2). After 1 h, no reaction was observed in the absence of additives. Addition of Zn(OTf)_2_ or [Cu(MeCN)_4_]PF_6_ led to rapid production of cyclopropanes **8** in comparable yield and diastereoselectivity to the reaction in which the guest was introduced prior to the substrate. [**4**AuCl] thus behaves as a switchable catalyst, with an extremely large difference in activity between the “off” and “on” states.

**scheme 2:**
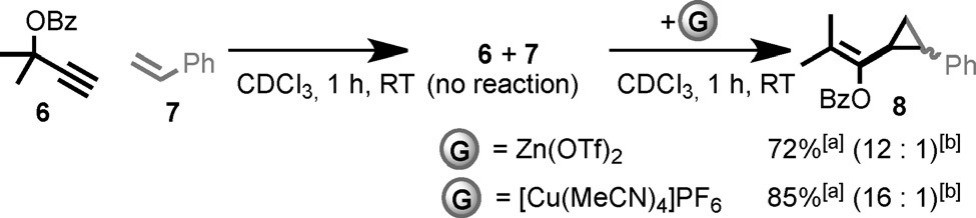
Switching Experiment. Reagents and Conditions: [4AuCl] (5 mol %), AgSbF_6_ (5 mol %), CDCl_3_, RT, 1 h then addition of guest (5 mol %) and a further 1 h at RT. [a] Determined by ^1^H NMR with Cl_2_CHCHCl_2_ as an internal standard. [b] Determined by HPLC analysis.

Secondly, the selectivity observed in the presence of Cu^I^ or TsOH is among the highest achieved by monodentate phosphines for these substrates.^25^ Since diastereoselectivity in Toste’s cyclopropanation reaction is correlated with ligand steric hindrance,^20^ we examined the steric demand of the rotaxane ligand by using Nolan’s % buried volume (%V_bur_) parameter, a gross measure of the volume around the metal center occupied by the ligand atoms.^26^ Applying the calculation^27^ to the solid-state structure of [**4**AuCl] (Figure 1) revealed a %V_bur_ value of 44 %, which is significantly higher than that of PPh_3_ (30 %) and even sterically hindered ligand P*t*Bu_3_ (38 %).^26^

Finally, and perhaps most strikingly, each guest examined gives rise to a different degree of diastereoselectivity. This variation is tentatively attributed to the modulation of the reaction field provided by the mechanical bond upon guest binding; as well as disrupting the N–Au coordination, the binding of guests into the macrocycle cavity will alter the co-conformation between the macrocycle and thread, thereby rigidifying the catalyst framework and modifying the space around the reaction site in a manner akin to allosteric modulation of enzymatic catalysts.

The serendipitous isolation of [**4**(H)(AuCl)]AuCl_2_ as a minor byproduct during the synthesis of [**4**AuCl] provides insight into the effect of guest binding on the steric environment around the gold. In the solid-state structure of [**4**(H)(AuCl)]^+^ (see the Supporting Information), the proton guest is located between a triazole nitrogen and one of the bipyridine nitrogens. This binding event causes a co-conformational rearrangement compared with [**4**AuCl]; the Au–Cl bond of [**4**(H)(AuCl)]AuCl_2_ is projected towards rather than away from the bipyridine unit (compare to Figure 1), which clearly alters the three dimensional environment around the Au center. The calculated %V_bur_ of 42 % for [**4**(H)(AuCl)]AuCl_2_ also differs from that of [**4**AuCl], albeit by only 2 %. Although neither [**4**AuCl] or [**4**(H)(AuCl)]AuCl_2_ is of direct catalytic relevance, the large co-conformational change observed on protonation provides evidence for the proposed allosteric role of the guest.

To investigate the generality of these observations, we compared the reactions of substrates with different steric properties mediated by [*t*Bu_3_PAuCl], [**5**AuCl], or [**4**AuCl] (Figure 3). In keeping with previous reports, alkyl-substituted cyclopropanes **9** and **12** were formed in low selectivity in the presence of [*t*Bu_3_PAuCl], whereas variation of the ester moiety between OAc (**10**) and pivalate (OPiv; **11**) led to no significant change in selectivity.20a This trend was repeated in the case of [**5**AuCl], although in all cases the selectivity was inferior to that of the bulkier *t*Bu_3_P ligand. Reactions mediated by [**4**AuCl] gave the target cyclopropane with significantly higher selectivity than that produced with either non-interlocked catalyst, thus further demonstrating the sterically hindered environment provided by the mechanical bond. Once again, the diastereoselectivity of reactions mediated by [**4**AuCl] varied in a guest-dependent manner. However, whereas [**4**AuCl] produced benzoate esters **8** and **9** in higher d.r. in the presence of Cu^I^ than Zn^II^, this trend was reversed in the case of alkyl ester derived products **10**–**12**. Thus, although the guest-dependent behavior of [**4**AuCl] is reproducible across the substrates investigated, the optimal guest appears to depend on the detailed structure of the reagents. This suggests that, in addition to a simple steric component, specific interactions between substrate and catalyst that vary with the identity of the guest may play a significant role.

**Figure 3:**
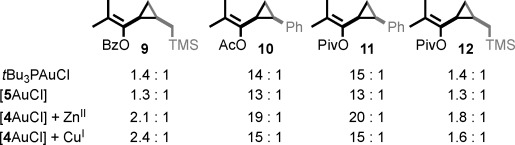
Comparison of *t*Bu_3_PAuCl, [5AuCl], and [4AuCl] with representative substrates. Selectivity determined by ^1^H NMR. Conditions as in Table 1.

In conclusion, we have demonstrated a rotaxane-based Au catalyst and identified a new approach to the development of stimuli-responsive interlocked catalysts more generally. Although such behavior has previously been demonstrated in organocatalytic rotaxanes,^5^ this is the first example of a rotaxane-based metal complex with stimuli-responsive catalytic activity. Once switched on by guest binding, the flexible but sterically crowded^28^ environment of the mechanical bond was shown to strongly influence the diastereoselectivity of an Au-mediated reaction, thus demonstrating the potential of interlocked molecules for the design of new reaction fields for hard-to-influence transformations. The influence of the mechanical bond is also responsive to external stimuli, depending as it does on the nature of the guest, and this is the first time that such stimuli-responsive stereoselectivity has been observed in a rotaxane catalyst. The origin of this effect is tentatively proposed to be similar to allosteric modulation in enzymes, in which cofactor binding subtly influences the environment of the active site. Modification of the reaction field by guest binding in such rotaxane architectures offers a supramolecular approach to the optimization of catalyst activity and selectivity, the potential for catalytic signal application for the development of sensors,^29^ and the possibility of controlling not just the stereoselectivity but also the chemoselectivity of metal-catalyzed reactions in a stimuli-responsive manner.

## Experimental Section

Switching experiment: CDCl_3_ (0.5 mL) was added to a sealed vial containing rotaxane [**4**AuCl] (7.1 mg, 0.0062 mmol) and AgSbF_6_ (2.2 mg, 0.0062 mmol). After 5 min, **6** (23.0 mg, 0.122 mmol) and **7** (50.9 mg, 0.489 mmol,) were added as solutions in CDCl_3_ (2.5 mL). A solution of 1,1,2,2-tetrachloroethane in CDCl_3_ (1.22 m, 0.1 mL) was added and the resulting mixture was stirred for 1 h and analyzed by ^1^H NMR spectroscopy. The mixture was transferred into a vial containing the guest (0.0062 mmol). After 1 h, the reaction was analyzed by ^1^H NMR and HPLC.

Full crystallographic data and characterization of all novel compounds is given in the Supporting Information. CCDC 1406074–1406077 contains the supplementary crystallographic data for this paper. These data are provided free of charge by The Cambridge Crystallographic Data Centre.

## Supporting Information

As a service to our authors and readers, this journal provides supporting information supplied by the authors. Such materials are peer reviewed and may be re-organized for online delivery, but are not copy-edited or typeset. Technical support issues arising from supporting information (other than missing files) should be addressed to the authors.

miscellaneous_information
