# Supplementary material for: A Stimuli-Responsive Rotaxane–Gold Catalyst: Regulation of Activity and Diastereoselectivity
Source: Angew Chem Int Ed Engl. 2015 Sep 21;54(46):13545–9. doi: 10.1002/anie.201505464 (PMC4678423; doi:10.1002/anie.201505464)
Supplement: Supplementary file 1 [file anie0054-13545-sd1.pdf]

Angewandte Chemie
Eine Zeitschrift der Gesellschaft Deutscher Chemiker

Supporting Information

A Stimuli-Responsive Rotaxane–Gold Catalyst: Regulation of Activity and Diastereoselectivity

*Marzia Galli, James E. M. Lewis, and Stephen M. Goldup**

anie_201505464_sm_miscellaneous_information.pdf

Table of Contents

1.	General Experimental Section	S2
2.	Experimental Procedures	S3
2.1	Synthesis of Novel Compounds	S3
2.2	Binding studies with [4AuCl]	S10
2.3	Analysis of cationic Au complexes	S11
2.4	Au(I)-Catalyzed Cyclopropanation reactions	S13
4.	X-Ray Data for Rotaxanes	S18
5.	NMR Data for Novel Compounds	S23
6.	References	S75

1. General Experimental Section

Unless otherwise stated, all reagents were purchased from commercial sources and used without further purification. Oxygen- and moisture-sensitive reactions were carried out under an atmosphere of N_2 using anhydrous solvents. Anhydrous solvents were obtained by passing the solvent through an activated alumina column on an MBRAUN MB SPS-800 solvent purification system. Petrol refers to the fraction of petroleum ether boiling in the range 40-60 °C. IPA refers to iso-propyl alcohol. Flash column chromatography was performed using Biotage Isolera-4 automated chromatography system, employing Biotage SNAP or ZIP cartridges. Analytical TLC was performed on pre-coated silica gel plates (0.25 mm thick, 60F254, Merck, Germany) and observed under 254 nm UV light. NMR spectra were recorded in $CDCl_3$, on Bruker AV400, AV3400, AV500 or AV600 instruments, at a constant temperature of 300 K. Chemical shifts δ (ppm) are reported in parts per million from low to high field and referenced to residual solvent. Coupling constants (*J*) are reported in Hertz (Hz). Standard abbreviations indicating multiplicity were used as follows: m = multiplet, quint. = quintet, q = quartet, t = triplet, d = doublet, s = singlet, br = broad. EDTA-NH_3 solution refers to an aqueous solution of NH_3 (17% w/w) saturated with sodium-ethylenediaminetetraacetate. Melting points were determined using a Sanyo Gallenkamp apparatus and are uncorrected. Low-resolution mass spectrometry was carried out by the mass spectrometry services at the Queen Mary University of London (Agilent SL Ion Trap MSD) or in Southampton using (Waters TQD mass spectrometer equipped with a triple quadrupole analyser with UHPLC injection [BEH C18 column; MeCN-hexane gradient {0.2% formic acid}]) . High-resolution mass spectrometry was carried out by the EPSRC National Mass Spectrometry in Swansea or the University of Southampton. To calculate diastereoselectivities for the cyclopropanation reaction, HPLC (Agilent Technologies 1120 LC) was run on a CHIRALCEL AD-H column using UV detection. The following compounds were obtained using literature procedures: macrocycle **1**,[1] 3-di-*tert*-butyl-5-ethynylbenzene (alkyne stopper) **2**,[2] (azidomethyl)diphenylphosphine oxide (azide stopper) **3**,[3] **6**,[4] **S3**[4] and **S4**.[4]

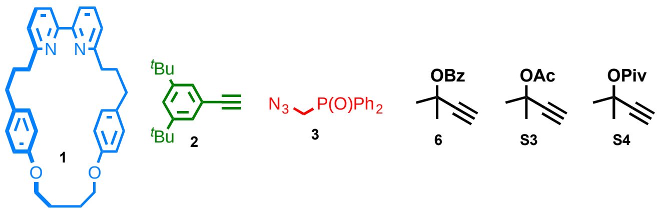

2. Experimental Procedures

2.1. Synthesis of Novel Compounds

2.1.0 Synthesis of [4AuCl]

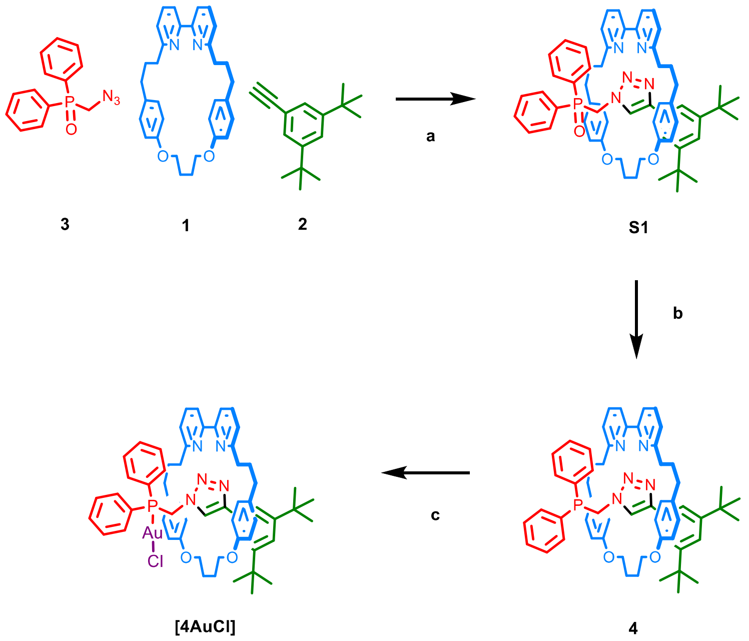

Scheme S1: Synthesis of Rotaxane-AuCl Complex. Reagents and conditions: a) $[Cu(MeCN)_4]PF_6$, $^{i}Pr_2EtN$, EtOH, 80 ºC, 18 h; b) Cl_3SiH, NEt_3, $PhMe/CH_2Cl_2$ (6:1), 100 ºC, 18 h c) Me_2SAuCl, CH_2Cl_2, rt, 1 h.

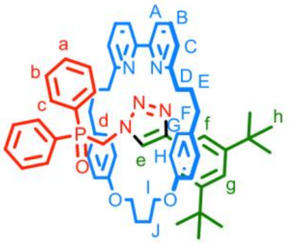

S1

Rotaxane S1: A dry CEM MW vial was charged with macrocycle **1** (12 mg, 0.025 mmol), azide **3** (6.4 mg, 0.025 mmol), ethynyl-3,5-di-*tert*-butylbenzene **2** (5.4 mg, 0.025 mmol), and $[Cu(MeCN)_4]PF_6$ (8.9 mg, 0.024 mmol). EtOH (2.5 mL) was added, followed by DIPEA (4.0 µL, 0.025 mmol) and the reaction mixture stirred at 80 °C for 18 h. After this time, the ethanolic phase was decanted and the remaining solid was washed with EtOH (3 × 2.5 mL). The solid was then dissolved in CH_2Cl_2 (30 mL) and washed with EDTA-$NH_{3\ (aq)}$ (30 mL) and brine (30 mL) and dried over $MgSO_4$. After evaporation of the solvent, rotaxane **S1** was obtained as a white solid (20.2 mg, 85% yield). m.p. = 195-197 ºC. ^{1}H NMR (500 MHz, $CDCl_3$) δ: 9.72 (s, 1H, H_e), 7.77-7.71 (m, 4H, H_c), 7.68 (t, J = 7.8 Hz, 2H, H_B), 7.56-7.49 (m, 4H, H_{A+a}), 7.47 (d, J = 2.0 Hz, 2H, H_f), 7.45-7.40 (m, 4H, H_b), 7.18 (t, J = 1.5 Hz, 1H, H_g), 7.14 (d, J = 7.8 Hz, 2H, H_C), 6.33 (d, J = 8.4 Hz, 4H, H_H), 6.26 (d, J = 8.4 Hz, 4H, H_G), 4.59 (d, J = 4.4 Hz, 2H, H_d), 4.27-4.17 (m, 4H, H_I), 2.48-2.29 (m, 8H, H_{D+F}), 2.27-2.18 (m, 2H, 2 of H_J), 2.06-1.95 (m, 2H, 2 of H_J), 1.70-1.56 (m, 4H, H_E), 1.20 (s, 18H, H_h). ^{13}C NMR (125 MHz, $CDCl_3$) δ: 163.5 (C), 157.7 (C), 157.4 (C), 149.5 (C), 145.8 (C), 136.7 (CH), 132.0 (CH), 131.9 (d, J_{CP}= 4.7 Hz, C), 131.5 (d, J_{CP}= 9.5 Hz, CH), 131.2 (C), 130.9 (C), 128.5 (d, J_{CP}= 12.1 Hz, CH), 128.3 (CH), 125.2 (CH), 121.6 (CH), 120.5 (CH), 120.3 (CH), 119.7 (CH), 115,2 (CH), 66.9 (C), 48.2 (d, J_{CP}= 74.0 Hz, CH_2), 36.9 (CH_2), 34.9 (CH_2), 34.7 (C), 31.4 (CH_3), 30.9 (CH_2), 24.9 (CH_2). ^{31}P NMR (202 MHz, $CDCl_3$) δ: 24.4. HRMS (EI +ve) 950.5120 $[M+H]^+$ (calc. for $C_{61}H_{69}N_5O_3P$ 950.5093 $[M+H]^+$).

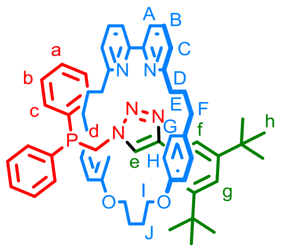

4

Rotaxane 4: $HSiCl_3$ (42 µL, 0.42 mmol) and Et_3N (117 µL, 0.843 mmol) were added to a sealed dry CEM MW vial containing rotaxane **S1** (40.0 mg, 0.042 mmol) in PhMe (1.2 mL) and CH_2Cl_2 (200 µL). The solution was stirred at 100 °C for 18 h. The reaction was then cooled, diluted with CH_2Cl_2 (10 mL) and washed with 1 M NaOH (5 mL) and brine (5 mL). The aqueous layers were extracted with CH_2Cl_2 (10 mL) and the combined organic layers were dried ($MgSO_4$), filtered and the solvent removed *in vacuo*. The crude mixture was filtered through a pad of celite and silica and eluted with $CHCl_3$/IPA 3:1 (50 mL). Removal of solvent *in vacuo* provided phosphine **4** as a white solid (36.1 mg, 92% yield) m.p. = 171-173 °C. ^{1}H NMR (500 MHz, $CDCl_3$) δ: 10.04 (s, 1H, H_e), 7.63 (t, *J* = 6.5 Hz, 2H, H_B), 7.60 (d, *J* = 1.5 Hz, 2H, H_f), 7.47 (d, *J* = 6.5 Hz, 2H, H_A), 7.20-7.16 (m, 7H, H_{a+b+g}), 7.15-7.11 (m, 4H, H_c) 7.10 (d, *J* = 6.5 Hz, H_C), 6.52-6.46 (m, 8H, H_{G+H}), 4.67-4.60 (m, 2H, 2 of H_I), 4.19 (s, 2H, H_d), 4.18-4.13 (m, 2H, 2 of H_I), 2.55-2.48 (m, 2H, 2 of H_F), 2.41-2.28 (m, 6H, 2 of H_F + H_D), 2.26-2.20 (m, 2H, 2 of H_J), 2.12-2.04 (m, 2H, 2 of H_J), 1.77-1.67 (m, 2H, 2 of H_E), 1.60-1.51 (m, 2H, 2 of H_E), 1.17 (s, 18H, H_h). ^{13}C NMR (125 MHz, $CDCl_3$) δ: 163.3 (C), 157.5 (C), 157.2 (C), 150.2 (C), 146.9 (C), 137.5 (d, J_{CP} = 17.6 Hz, C), 136.7 (CH), 133.0 (d, J_{CP} = 25.2 Hz, CH), 132.3 (CH), 131.7 (C), 128.9 (CH), 128.6 (CH), 128.2 (d, J_{CP}= 8.2 Hz, CH), 124.8 (C), 121.6 (CH), 120.5 (CH), 120.5 (CH), 119.8 (CH), 115.3 (CH), 66.7 (C), 49.9 (d, J_{CP} = 70 Hz, CH_2), 36.9 (CH_2), 35.3 (CH_2), 34.9 (C), 31.5 (CH_3), 31.2 (CH_2), 25.0 (CH_2). ^{31}P NMR (202 MHz, $CDCl_3$) δ: -15.3. HRMS (EI +ve) 934.5176 $[M+H]^+$ (calc. for $C_{61}H_{69}N_5O_2P$ 934.5144 $[M+H]^+$).

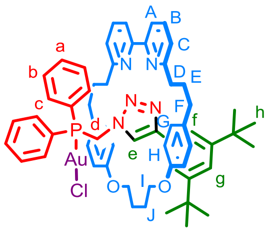

[4AuCl]

Rotaxane [4AuCl]: Rotaxane **4** (20 mg, 0.021 mmol) was stirred for 1 h in CH_2Cl_2 (2 mL) at rt with Me_2SAuCl (5.9 mg, 0.020 mmol). The reaction mixture was then filtered through celite and the solvent removed *in vacuo*. Recrystallization with a 1:1 mixture of hexane/CH_2Cl_2 afforded rotaxane **[4AuCl]** as white fibrous crystals (22.2 mg, 95% yield) m.p. = 224-226 °C. ^{1}H NMR (600 MHz, $CDCl_3$) δ: 9.48 (s, 1H, H_e), 7.69 (t, J = 7.8 Hz, 2H, H_B), 7.66-7-60 (m, 4H, H_c), 7.56-7.51 (m, 4H, H_{A+a}), 7.46-7.39 (m, 6H, H_{b+f}), 7.24-7.21 (m, 1H, H_g), 7.13 (d, J = 7.8 Hz, 2H, H_C), 6.42-6.36 (m, 8H, H_{G+H}), 4.60 (d, 2H, J_{HP} = 6.6 Hz, H_d), 4.34-4.24 (m, 4H, H_I), 2.42-2.22 (m, 10H, H_{D+F} + 2 of H_J), 2.11-2.00 (m, 2H, 2 of H_J), 2.67-2.58 (m, 2H, 2 of H_E), 1.53-1.46 (m, 2H, 2 of H_E), 1.24 (s, 18H, H_h). ^{13}C NMR (151 MHz, $CDCl_3$) δ: 163.0 (C), 157.9 (C), 157.4 (C), 149.8 (C), 145.9 (C), 136.9 (CH), 133.7 (d, J_{CP} = 14.0 Hz, CH), 132.2 (CH), 131.9 (C), 130.6 (C), 129.3 (d, J_{CP} = 11.8 Hz, CH), 128.3 (CH), 127.8 (d, J_{CP} = 59.2 Hz, C), 124.7 (CH), 121.8 (CH), 120.6 (CH), 120.5 (CH), 120.1 (CH), 115.2 (CH), 67.0 (C), 46.8 (d, J_{CP} = 40.8 Hz, CH_2), 36.9 (CH_2), 34.7 (CH_2), 31.5 (CH_3), 30.9 (CH_2), 29.7 (CH_2), 24.8 (CH_2). ^{31}P NMR (240 MHz, $CDCl_3$) δ: 25.5. LRMS (ESI +ve) for $C_{61}H_{69}AuClN_5O_2P$: 1166.4 m/z $[M+H]^+$.

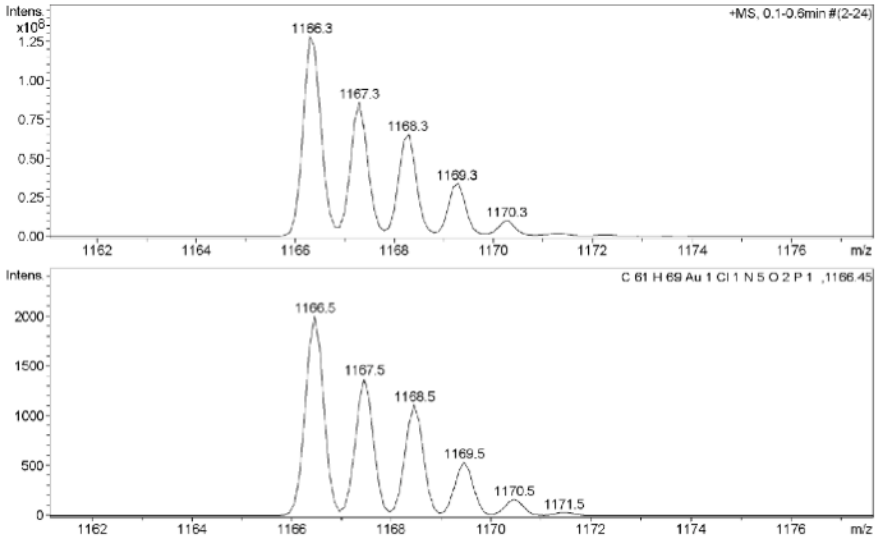

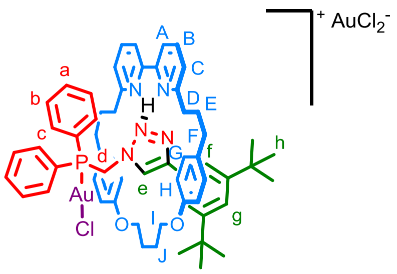

[4AuClH]AuCl$_2$

Note: If care was not taken in the neutralization of **4** after the reduction step, a white precipitate was observed to form during the synthesis of [**4**AuCl]. The solid was collected by suction filtration and redissolved in $CDCl_3$. Slow evaporation gave crystals suitable for single crystal x-ray diffraction. X-ray analysis revealed the byproduct to be [**4**(H)AuCl]$AuCl_2$, presumably formed by reaction of [**4**AuCl] with HCl carried through from the reduction step. Vigorous washing with aqueous NaOH during the work up of **4** prevented the formation of this byproduct: m.p. = 264-266 °C. ^{1}H NMR (400 MHz, $CDCl_3$) δ: 8.31-8.17 (m, 4H, H_{A+B}), 7.86-7.77 (m, 4H, H_c), 7.66-7.50 (m, 8H, H_{b+a+C}), 7.37-7.34 (m, 1H, H_g), 7.23-7.19 (m, 2H, H_f), 6.72-6.63 (m, 8H, H_{G+H}), 5.94 (s, 1H, H_e), 5.08 (d, J = 8.0 Hz, 2H, H_d), 4.22-4.12 (m, 4H, H_I), 3.16-3.06 (m, 4H, H_J), 2.59-2.43 (m, 4H, H_D), 2.41-2.31 (m, 4H, H_F), 1.62-1.47 (m, 4H, H_E), 1.18 (s, 18H, H_h). ^{31}P NMR (160 MHz, $CDCl_3$) δ: 25.6. LRMS (ESI +ve): 1166.4 m/z $[M+H]^+$.

2.4.1 Synthesis of [5AuCl]

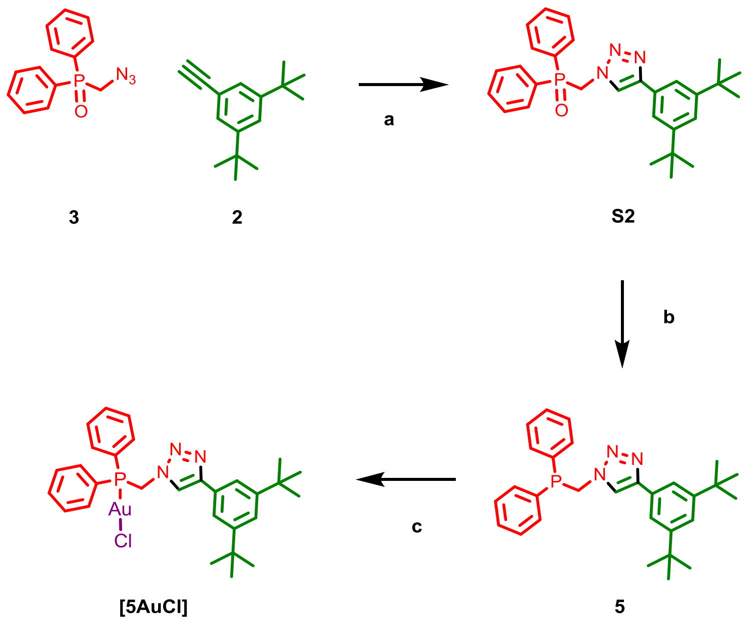

Scheme S2: Synthesis of Thread-AuCl Complex. Reagents and conditions: a) $[Cu(MeCN)_4]PF_6$, CH_2Cl_2, rt, 2 h; **b)** Cl_3SiH, NEt_3, PhMe/CH_2Cl_2 (6:1), 100 °C, 18 h c) Me_2SAuCl, CH_2Cl_2, rt, 1 h.

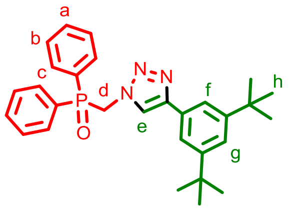

S2

Thread S2: A dry CEM MW vial was charged with azide **3** (6.4 mg, 0.025 mmol), ethynyl-3,5-di-*tert*-butylbenzene **2** (5.4 mg, 0.025 mmol), and $[Cu(MeCN)_4]PF_6$ (9.0 mg, 0.024 mmol). CH_2Cl_2 (2.5 mL) was added and the reaction mixture stirred at rt for 2 h. After this time the reaction mixture was poured into EDTA-$NH_{3(aq)}$ (30 mL) and extracted with CH_2Cl_2 (3 × 30 mL). The combined organic extracts were washed with brine (30 mL) and dried over $MgSO_4$. Removal of solvent and column chromatography (0-20% Et_2O in $CHCl_3$) afforded product **S2** as a light-orange solid (10.3 mg, 87% yield). m.p. = 100-102 ºC. ^{1}H NMR (600 MHz, $CDCl_3$) δ: 8.17 (s, 1H, H_e), 7.80-7.75 (m, 4H, H_c), 7.63 (d, J = 1.8 Hz, 2H, H_f), 7.60-7.56 (m, 2H, H_a), 7.52-7.48 (m, 4H, H_b), 7.40 (t, J = 1.8 Hz, 1H, H_g), 5.28 (d, J_{HP} = 6.6 Hz, 2H, H_d), 1.35 (s, 18H, H_h). ^{13}C NMR (151 MHz, $CDCl_3$) δ: 151.5 (C), 149.2 (C), 133.1 (d, J_{CP}= 2.7 Hz, CH), 131.2 (d, J_{CP}= 9.7 Hz, CH), 129.5 (d, J_{CP}= 7.6 Hz, C), 129.1 (d, J_{CP}= 12.1 Hz, CH), 128.5 (C), 122.5 (CH), 120.8 (CH), 120.1 (CH), 50.3 (d, J_{CP}= 71.6 Hz, CH_2), 34.9 (C), 31.4 (CH_3). ^{31}P NMR (240 MHz, $CDCl_3$) δ: 26.2. HRMS (EI +ve) 472.2501 $[M+H]^+$ (calc. for $C_{29}H_{35}N_3OP$ 472.2439 $[M+H]^+$).

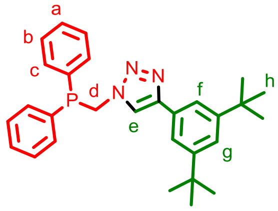

5

Thread 5: Cl_3SiH (42 µL, 0.42 mmol) and Et_3N (117 µL, 0.843 mmol) were added to a sealed dry CEM MW vial containing solution of **S2** (20.0 mg, 0.042 mmol) in PhMe (1.37 mL) and CH_2Cl_2 (230 µL). The solution was stirred at 100 ºC for 18 h. The reaction was then cooled, diluted with CH_2Cl_2 (10 mL) and washed with aqueous 1M NaOH (5 mL) and brine (5 mL). The aqueous layers were then extracted with CH_2Cl_2 (10 mL). The combined organic layers were dried over $MgSO_4$, and the solvent removed *in vacuo*. The crude mixture was filtered through a pad of celite and silica and the pad eluted with $CHCl_3$/IPA 3:1 (50 mL). Removal of solvent *in vacuo* provided phosphine **5** as a yellow solid (15.0 mg, 78% yield). m.p. = 97-99 ºC. ^{1}H NMR (600 MHz, $CDCl_3$) δ: 7.57-7.54 (m, 2H, H_f), 7.50-7.37 (m, 12H, $H_{a+b+c+g+e}$), 5.13 (d, J = 5.4 Hz, 2H, H_d), 1.35 (s, 18H, H_h). ^{13}C NMR (151 MHz, $CDCl_3$) δ: 151.3 (C), 148.8 (C), 134.4 (d, J_{CP} = 12.1 Hz, C), 133.1 (d, J_{CP} = 19.2 Hz, CH), 131.3 (d, J_{CP} = 9.09 Hz, CH), 129.8 (C), 129.0 (d, J_{CP} = 7.1 Hz, CH), 122.3 (CH), 120.1

(CH), 119.7 (CH), 50.2 (d, J_{CP} = 21.9 Hz, CH_2), 34.9 (C), 31.4 (CH_3). ^{31}P NMR (240 MHz, $CDCl_3$) δ: -13.9. HRMS (EI +ve) not possible to evaluate because the phosphine is highly prone to reoxidation.

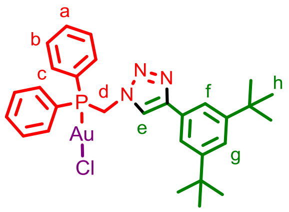

[5AuCl]

Thread [5AuCl]: Thread **5** (18 mg, 0.041 mmol) was stirred for 1 h in CH_2Cl_2 (2 mL) at rt with Me_2SAuCl (12 mg, 0.041 mmol). The reaction mixture was then filtered through basic alumina eluting with dry CH_2Cl_2. Removal of solvent *in vacuo* afforded thread **[5AuCl]** as a white solid (25.3 mg, 92% yield). m.p. = 152-154 °C. ^{1}H NMR (500 MHz, $CDCl_3$) δ: 7.82 (s, 1H, H_e), 7.75-7.68 (m, 4H, H_c), 7.63-7.58 (m, 2H, H_a), 7.55 (d, J = 2.0 Hz, 2H, H_f), 7.54-7.51 (m, 4H, H_b), 7.42 (t, J = 2.0 Hz, 1H, H_g), 5.49 (d, J = 3.5 Hz, 2H, H_d), 1.35 (s, 18H, H_h). ^{13}C NMR (125 MHz, $CDCl_3$) δ: 151.6 (C), 149.7 (C), 134.0 (d, J_{CP} = 13.8 Hz, CH), 133.3 (d, J_{CP} = 2.5 Hz, CH), 129.9 (d, J_{CP} =12.0 Hz, C), 129.0 (C), 125.3 (d, J_{CP} = 59.4 Hz, C), 123.0 (CH), 120.5 (CH), 120.5 (CH), 49.3 (d, J_{CP} = 36.0 Hz, CH_2), 35.1 (C), 31.6 (CH_3). ^{31}P NMR (202 MHz, $CDCl_3$) δ: 29.1. HRMS (ESI +ve): 688.1922 m/z $[M]^+$ (calc. for $C_{29}H_{34}N_3PAuCl$ 688.1917 $[M^+]$).

2.2 Guest binding studies

Rotaxane [**4**AuCl] (8.8 mg, 0.0075 mmol) and the guest (0.0075 mmol) were dissolved in $CDCl_3$ (0.6 mL), sonicated for 15 min at rt., filtered through celite and analyzed by ^{1}H and ^{31}P NMR.

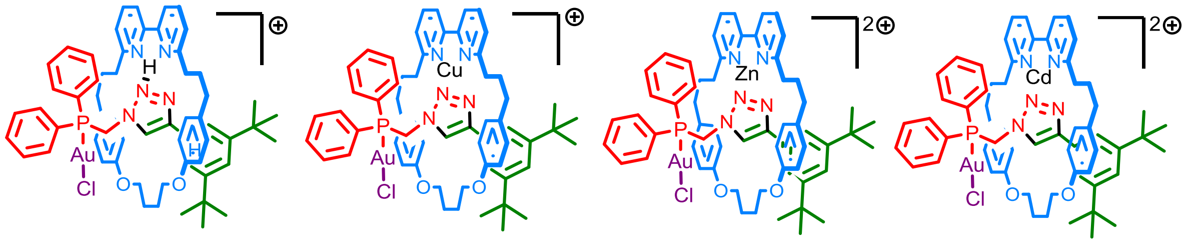

[**4**AuClH]OTs [**4**AuClCu]PF_6 [**4**AuClZn]$(OTf)_2$ [**4**AuClCd]$(OTf)_2$

Note: the detailed structures of the guest complexes are unclear at the stage – for instance the Zn and Cd may retain one or more of the OTf anions. The structures shown are for illustrative purposes only.

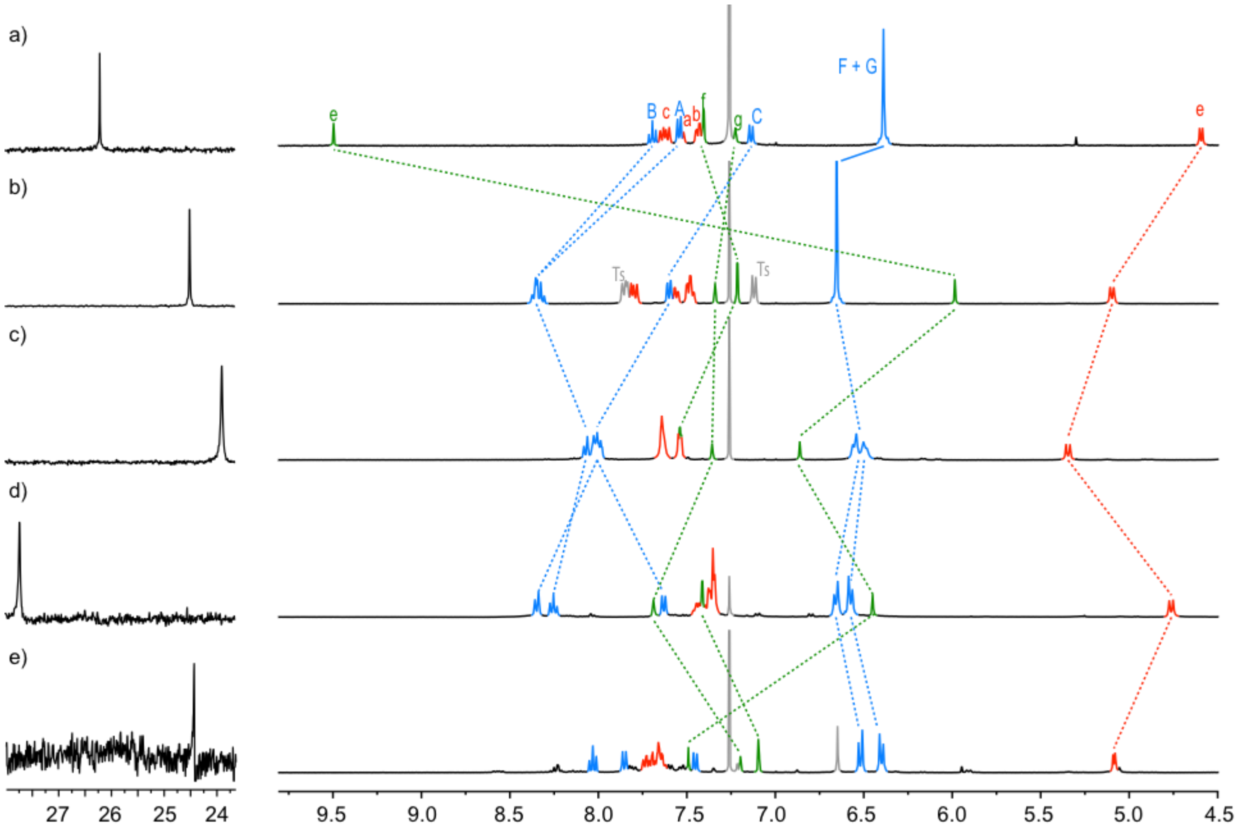

Fig S1: ^{31}P and ^{1}H NMR spectra of a) [**4**AuCl] and the complexes formed on addition of 1 equiv. b) HOTs; c) $[Cu(MeCN)_4]PF_6$; d) $Zn(OTf)_2$; e) $Cd(OTf)_2$. Peaks are assigned in accordance with compound labelling above. Solvent and counterion (TsO) peaks are shown in grey.

2.3 Analysis of cationic Au complexes

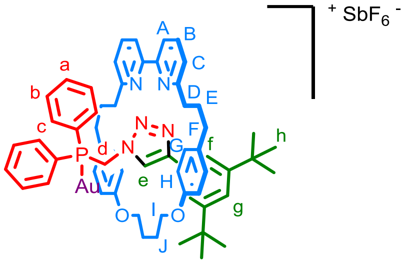

[4Au]SbF$_6$

Rotaxane [4Au]SbF$_6$: an NMR tube was charged with the rotaxane [**4**AuCl] (8.8 mg, 0.0075 mmol) and with AgSbF$_6$ (2.6 mg, 0.0075 mmol) and was sonicated for 15 minutes in CDCl$_3$ (0.6 mL) at rt. The reaction mixture was then filtered through celite and analyzed directly by ^{1}H NMR spectroscopy: ^{1}H NMR (600 MHz, CD$_2$Cl$_2$) δ: 8.00 (t, J = 7.8 Hz, 2H, H$_B$), 7.77-7.72 (m, 2H, H$_a$), 7.72-7.69 (m, 3H, H$_{A+e}$), 7.68-7.65 (m, 4H, H$_c$), 7.61-7.57 (m, 4H, H$_b$), 7.46 (d, J = 7.2 Hz, H$_C$), 7.21 (t, J = 1.8 Hz, H$_g$), 7.07 (d, J = 1.8 Hz, H$_f$), 6.55-6.48 (m, 8H, H$_{G+H}$), 5.10 (d, J = 4.2 Hz, H$_d$), 3.87-3.80 (m, 2H, 2 of H$_I$), 4.03-3.95 (m, 2H, 2 of H$_I$), 2.83-2.77 (m, 2H, 2 of H$_F$), 2.64-2.50 (m, 4H, H$_D$), 2.16-2.08 (m, 2H, 2 of H$_F$), 1.88-1.81 (m, 6H, H$_J$ + 2 of H$_E$), 1.69-1.61 (m, 2H, 2 of H$_E$), 1.19 (s, 18H, H$_h$). ^{13}C NMR (150 MHz, CD$_2$Cl$_2$) δ (ppm) 163.8 (C), 158.6 (C), 156.5 (C), 151.3 (C), 148.9 (C), 141.0 (CH), 134.1 (d, J_{CP} = 12.0 Hz, C), 133.8 (d, J_{CP} = 13.5 Hz, CH), 133.14 (CH), 130.5 (d, J_{CP} = 12 Hz, CH), 129.7 (C), 129.5 (C), 125.7 (CH), 123.9 (CH), 122.5 (CH), 121.7 (CH), 119.8 (CH), 116.3 (CH), 100.6 (CH), 68.1 (C), 50.2 (d, J_{CP} = 54.5 Hz, CH$_2$), 39.0 (CH$_2$), 35.3 (CH$_2$), 35.3 (CH$_2$), 33.3 (CH$_2$), 31.8 (CH$_3$), 25.4 (CH$_2$). ^{31}P NMR (202 MHz, CD$_2$Cl$_2$) δ: 38.9. LRMS (ESI +ve) for C$_{61}$H$_{68}$AuN$_5$O$_2$P 1130 m/z [M+H]$^+$.

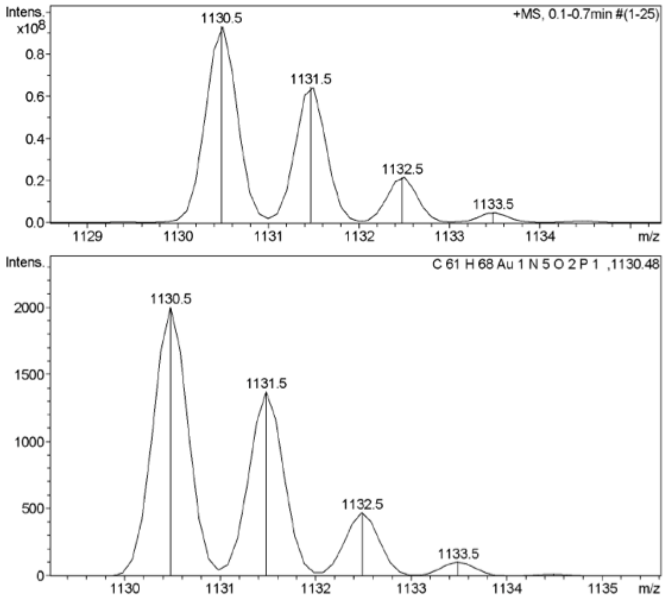

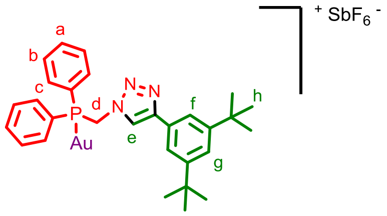

[5Au]SbF$_6$

Thread [5Au]SbF$_6$: An NMR tube was charged with the thread **[5AuCl]** (5.2 mg, 0.0075 mmol) and AgSbF$_6$ (2.6 mg, 0.0075 mmol) and was sonicated for 15 minutes in CD_2Cl_2 (0.6 mL) at rt. The reaction mixture was then filtered through celite and analyzed directly by ^{1}H NMR spectroscopy. ^{1}H NMR (500 MHz, CD_2Cl_2) δ: 8.29 (s, 1H, H_e), 7.85-7.78 (m, 4H, H_c), 7.77-7.72 (m, 2H, H_a), 7.68-7.64 (m, 4H, H_{b+f}), 7.60 (t, J = 2 Hz, 1H, H_g), 5.89 (d, J = 5.1 Hz, 2H, H_d), 1.26 (s, 18H, H_h). ^{13}C NMR (125 MHz, CD_2Cl_2) δ (ppm) 152.8 (C), 134.3 (d, J_{CP} = 2.5 Hz, CH), 133.6 (d, J_{CP} = 15.0 Hz, CH), 130.5 (d, J_{CP} = 12.5 Hz, CH), 126.0 (CH), 125.6 (C), 124.9 (C), 123.1 (C), 122.6 (CH), 121.3 (CH), 50.8 (d, J_{CP} = 41.3 Hz, CH_2), 34.9 (C), 30.9 (CH_3). ^{31}P NMR (202 MHz, CD_2Cl_2) δ: 34.6. HRMS (ESI +ve) for $C_{29}H_{34}N_3PAu$ 652.2136 m/z $[M+H]^+$ (calc. for $C_{29}H_{34}N_3PAu$ 652.2150 $[M+H]^+$).

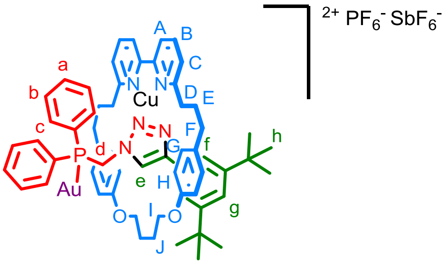

[4AuCu]PF$_6$SbF$_6$

Rotaxane [4AuCu]PF$_6$SbF$_6$: An NMR tube was charged with the rotaxane [**4**AuCl] (8.8 mg, 0.0075 mmol) and $[Cu(MeCN)_4]PF_6$ (2.8 mg, 0.0075 mmol) and was sonicated for 15 minutes in $CDCl_3$ (0.6 mL) at rt. After the solution turned yellow, implying the formation of the copper complex, AgSbF$_6$ (2.6 mg, 0.0075 mmol) was added and the solution was sonicated for further 15 minutes. The reaction mixture was then filtered through celite and analyzed directly by ^{1}H NMR spectroscopy. ^{1}H NMR (400 MHz, $CDCl_3$) δ: 8.20 (t, J = 7.6 Hz, 2H, H_B), 7.77 (bd, 2H, H_A), 7.73-7.60 (m, 11H, $H_{a+b+c+g}$), 7.57 (d, J = 8.0 Hz, 2H, H_C), 7.53 (bs, 1H, H_e), 6.84-6.74 (m, 2H, H_f), 6.54-6.48 (m, 4H, H_H), 6.30-6.23 (m, 4H, H_G), 5.16-5.07 (m, 2H, H_d), 4.12-4.00 (m, 4H, H_I), 3.03-2.87 (m, 4H, H_J), 2.47-2.38 (m, 2H, $H_{D'}$), 2.32-2.21 (m, 2H, $H_{D''}$), 2.00-1.88 (m, 4H, H_F), 1.66-1.49 (m, 4H, H_E), 1.36 (s, 18H, H_h). ^{31}P NMR (160 MHz, $CDCl_3$) δ: 43.3. LRMS (ESI +ve): 1196 m/z $[M+H]^+$.

2.4 Cyclopropanation Reactions

General procedure for Au(I)-Catalyzed Cyclopropanation reactions[4]:

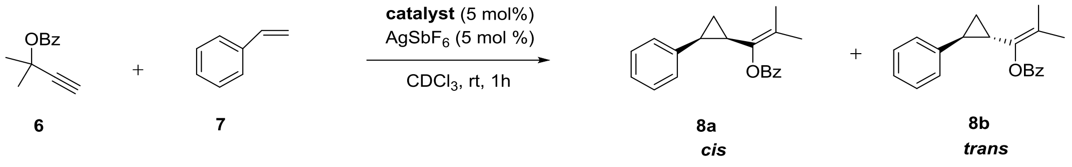

$CDCl_3$ (0.5 mL) was added to a MW CEM vial containing rotaxane [**4**AuCl] (7.1 mg, 0.0062 mmol) (and the guest (0.0062 mmol) where applicable). $AgSbF_6$ (2.1 mg, 0.0062 mmol) was added. After 5 minutes **6** (23.0 mg, 0.122 mmol, 1 equiv.) and **7** (50.9 mg, 0.489 mmol,) were added as solutions, each in 2.5 mL $CDCl_3$. After 1 h a solution of 1,1,2,2-tetrachloroethane (20.5 mg, 0.122 mmol) in $CDCl_3$ (0.1 mL) was added as an internal standard and the reaction mixture was analysed by ^{1}H NMR and HPLC.

General procedure for the *in situ* switching experiments with [4AuCl]

$CDCl_3$ (0.5 mL) was added to a MW CEM vial containing rotaxane [**4**AuCl] (7.1 mg, 0.0062 mmol) and $AgSbF_6$ (2.1 mg, 0.0062 mmol). After 5 minutes **6** (23.0 mg, 0.122 mmol, 1 equiv.) and **7** (50.9 mg, 0.489 mmol,) were added as solutions, each in 2.5 mL $CDCl_3$. A solution of 1,1,2,2-tetrachloroethane (20.5 mg, 0.122 mmol) in $CDCl_3$ (0.1 mL) was added as an internal standard. The resulting mixture was stirred for 1 h and analysed by ^{1}H NMR. No conversion was observed. The solution was then syringed into another MW CEM vial containing the metal salt (0.0062 mmol). The mixture was then stirred for a 1 h. and the reaction was analysed by ^{1}H NMR and HPLC.

2.4.1 HPLC Analysis of Diastereomeric Ratio for Cyclopropane 8

Samples were analysed on an AD-H column at 25 °C with a concentration of ~1 × 10^{-4} M and injection volume = 5 µL, in a solution of chloroform, with an isocratic gradient of 99:1 hexane/IPA at a flow rate of 1 mL/min.

Fig S2. Example HPLC report for the reaction of [4AuCl] in the presence of $Cu(MeCN)_4]PF_6$

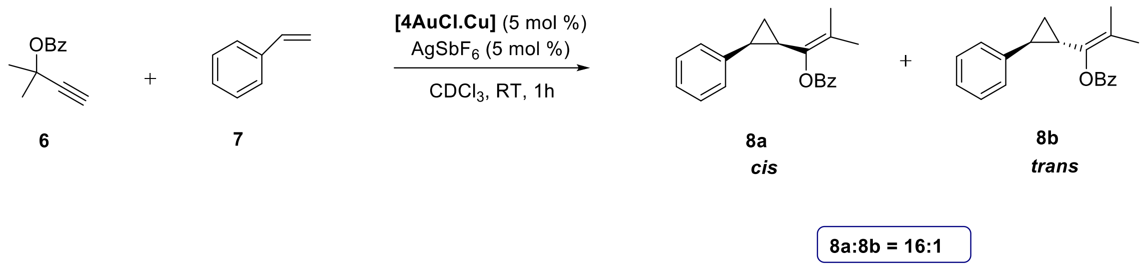

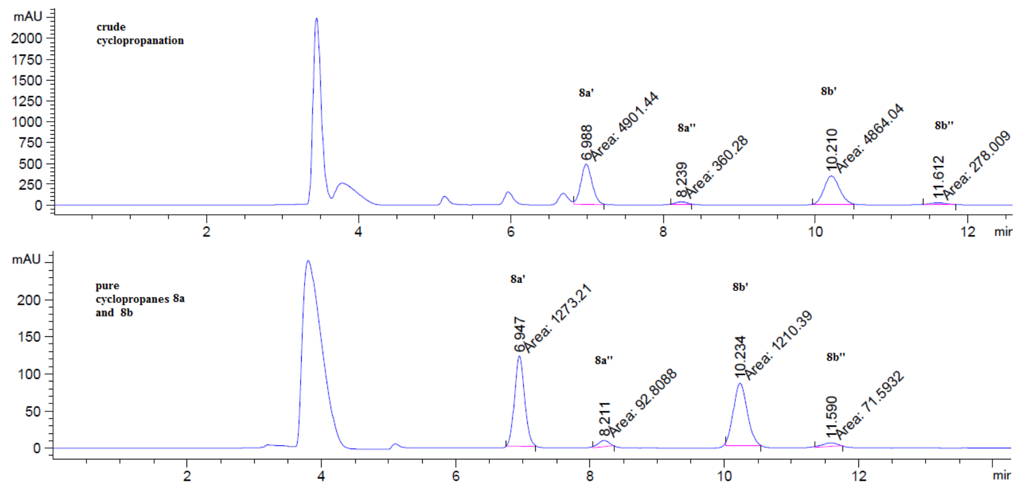

Table S1. Control Reactions between 6 and 7 in the Absence of Au^I

Entry[a]	Additive	Yield[b] (%)
1[c]	--	0
2	$AgSbF_6$	0
3	$[Cu(MeCN)_4]PF_6$	0
4	$Zn(OTf)_2$	0
5	TsOH	0

[a] Yields were measured after 1 h by ^1H NMR analysis by using an internal standard (1,1,2,2-tetrachloroethane).

2.4.2 Effect of ligand on reactions to produce cyclopropane 9

Reactions between **6** and allyl TMS were performed using the general procedure described above to give cyclopropane **9** as a mixture of diastereoisomers that were analyzed by ^{1}H NMR with tetrachloroethane as an internal standard to determine yield and diastereoisomeric ratio. To aid analysis, novel cyclopropane **9** was isolated as an inseparable mixture of isomers and characterised:

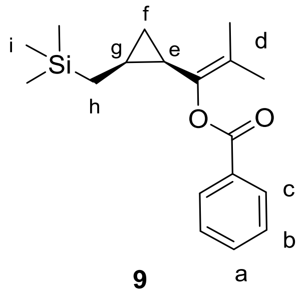

9

^{1}H NMR (400 MHz, $CDCl_3$) δ: 8.10-8.01 (m, 2H, H_c), 7.61-7.53 (m, 1H, H_a), 7.47-7.38 (m, 2H, H_b), 1.84 (s, 3H, 3 of $H_{d-trans}$), 1.82 (s, 3H, 3 of H_{d-cis}), 1.63 (s, 3H, 3 of H_{d-cis}), 1.54 (s, 3H, 3 of $H_{d-trans}$), 1.48-1.44 (m, 1H, $H_{e-trans}$), 1.05-0.94 (m, 2H, $H_{e-cis+g-trans}$), 0.90-0.81 (m, 3H, $H_{g-cis+f-cis}$), 0.77-0.66 (m, 2H, $H_{f-trans}$), 0.43-0.35 (m, 2H, $H_{h-trans}$), 0.15-0.08 (m, 2H, H_{h-cis}), 0.00 (s, 9H, $H_{i-trans}$), -0.03 (m, 9H, H_{i-cis}). ^{13}C NMR (100 MHz, $CDCl_3$) δ (ppm) 164.9 (C), 133.2 (Cx2), 130.0 (CHx2), 129.0 (CH), 100.1 (C), 21.9 (CH_2), 20.7 (CH), 19.0 (CH), 18.0 (CH), 17.5 (CH), 16.6 (CH_2), 14.8 (CH_3), 14.7 (CH_3), 14.1 (CH_2), 12.3 (CH_2), -1.2, -1.4. HRMS (ESI +ve) for $C_{18}H_{26}NaO_2Si$ 325.1596 m/z $[M+Na]^+$ (calc. for $C_{18}H_{26}NaO_2Si$ 325.1594 m/z $[M+Na]^+$).

Table S2. Effect of ligand on the reaction of 6 and allyl TMS to give 9

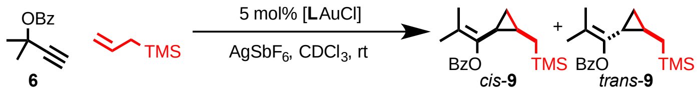

Entry	[LAuCl]	Additive	Yield[a] (*cis-trans*[a])
1	$^{t}Bu_3PAuCl$	-	35% (1.4 : 1)
2	[**5**AuCl]	-	56% (1.3 : 1)
3	[**4**AuCl]	$Zn(OTf)_2$	43% (2.1 : 1)
4	[**4**AuCl]	$[Cu(MeCN)_4]PF_6$	51% (2.4 : 1)

[a] Determined by ^{1}H NMR with $Cl_2CHCHCl_2$ as internal standard.

2.4.3 Effect of ligand on reactions to produce cyclopropane 10

Reactions between **S3** and sytrene were performed using the general procedure described above to give cyclopropane **10** as a mixture of diastereoisomers that were analysed by ^{1}H NMR by reference to previously reported data[4] and using tetrachloroethane as an internal standard to determine yield and diastereoisomeric ratio.

Table S3. Effect of ligand on the reaction of S3 and styrene to give 10

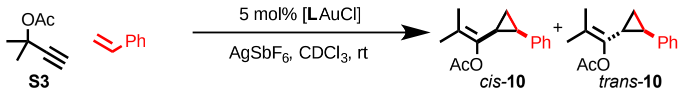

Entry	[LAuCl]	Additive	Yield[a] (*cis-trans*[a])
1	^{t}Bu$_3$PAuCl	-	37% (14 : 1)
2	[**5**AuCl]	-	71% (13 : 1)
3	[**4**AuCl]	$Zn(OTf)_2$	86% (19 : 1)
4	[**4**AuCl]	$[Cu(MeCN)_4]PF_6$	81% (15 : 1)

[a] Determined by ^{1}H NMR with $Cl_2CHCHCl_2$ as internal standard.

2.4.4 Effect of ligand on reactions to produce cyclopropane 11

Reactions between **S4** and sytrene were performed using the general procedure described above to give cyclopropane **11** as a mixture of diastereoisomers that were analysed by ^{1}H NMR with reference to previously reported data[4] and using tetrachloroethane as an internal standard to determine yield and diastereoisomeric ratio.

Table S4. Effect of ligand on the reaction of S4 and styrene to give 11

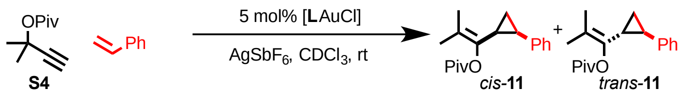

Entry	[LauCl]	Additive	Yield[a] (*cis-trans*[a])
1	^{t}Bu$_3$PauCl	-	49% (15 : 1)
2	[**5**AuCl]	-	85% (10 : 1)
3	[**4**AuCl]	$Zn(OTf)_2$	42% (20 : 1)
4	[**4**AuCl]	$[Cu(MeCN)_4]PF_6$	62% (15 : 1)

[a] Determined by ^{1}H NMR with $Cl_2CHCHCl_2$ as internal standard.

2.4.5 Effect of ligand on reactions to produce cyclopropane 12

Reactions between **S4** and allyl TMS were performed using the general procedure described above to give cyclopropane **12** as a mixture of diastereoisomers that were analysed by ^{1}H NMR with reference to previously reported data[4] and using tetrachloroethane as an internal standard to determine yield and diastereoisomeric ratio.

Table S5. Effect of ligand on the reaction of S4 and allyl TMS to give 12

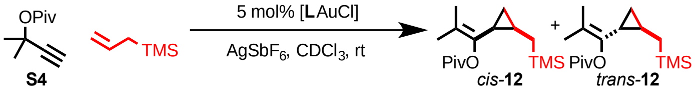

Entry	[LAuCl]	Additive	Yield[a] (*cis-trans*[a])
1	^{t}Bu$_3$PAuCl	-	40% (1.4 : 1)
2	[**5**AuCl]	-	90% (1.3 : 1)
3	[**4**AuCl]	$Zn(OTf)_2$	>95% (1.8 : 1)
4	[**4**AuCl]	$[Cu(MeCN)_4]PF_6$	>95% (1.6 : 1)

[a] Determined by ^{1}H NMR with $Cl_2CHCHCl_2$ as internal standard.

3 X-Ray Crystallographic Data

X-ray data for rotaxane **S1** were collected at 100 K on a KAPPA APEX II DUO diffractometer with Bruker APEX-II CCD area detector using Mo Kα radiation. The structures were solved and refined against F_2 using anisotropic thermal displacement parameters for all non-hydrogen atoms using Olex2. software. Except where noted hydrogen atoms were placed in calculated positions and refined using a riding model.

X-ray data for [**4**AuCl] and [**4**HAuCl]$AuCl_2$ were collected at 100 K with a Rigaku AFC12 goniometer equipped with an enhanced sensitivity (HG) Saturn724+ detector mounted at the window of an FR-E+ SuperBright molybdenum rotating anode generator with VHF Varimax optics (70µm focus). The structures were solved by SUPERFLIP and refined against F_2 using anisotropic thermal displacement parameters for all non-hydrogen atoms using SHELXL-2014/7 software. Except where noted hydrogen atoms were placed in calculated positions and refined using a riding model.

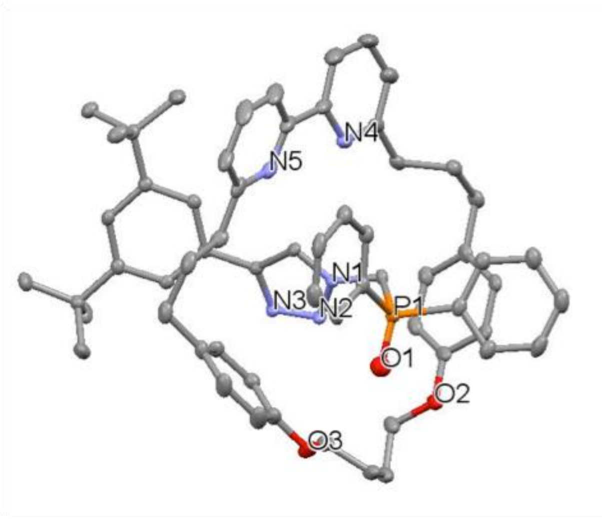

Figure S3. Ellipsoid plot of rotaxane S1. Hydrogens were omitted for clarity. Ellipsoids are shown at 50% probability. Bond lengths (Å) and angles (º): P1-O1 1.448(2); C7-P1-O1 113.3(2). Single crystals suitable for X-ray diffraction were obtained by slow diffusion of diethyl ether into a solution of compound **4** in $CHCl_3$.

CCDC No.	1406075	
Empirical formula	$C_{61}H_{68}N_5O_3P$	
Formula weight	950.17	
Temperature	100 K	
Wavelength	0.71073 Å	
Crystal system	Orthorhombic	
Space group	$P2_12_12_1$	
Unit cell dimensions	a = 9.1879 (2) Å	α = 90°
	b = 10.8708 (3) Å	β = 90°
	c = 51.6162 (12) Å	γ = 90°
Volume	5155.4 (2) $Å^3$	
Z	4	
Density (calculated)	1.224 Mg/m^3	
Absorption coefficient	0.868 mm^{-1}	
F(000)	2032.0	
Crystal size	0.20 × 0.15 × 0.05 mm^3	
Theta range for data collection	1.71 to 66.63°	
Index ranges	$-10\leq h\leq 10$, $-8\leq k\leq 12$, $-61\leq l\leq 58$	
Reflections collected	55943	
Independent reflections	8642 [R(int) = 0.0322]	
Completeness to theta = 66.63°	96.8 %	
Refinement method	Full-matrix least-squares on F^2	
Data / restraints / parameters	8642/ 0 / 637	
Goodness-of-fit on F^2	1.090	
Final R indices [I>2sigma(I)]	R_1 = 0.0407, wR_2 = 0.1024	
R indices (all data)	R_1 = 0.0423, wR_2 = 0.1034	
Largest diff. peak and hole	9.135 and -3.554 e.$Å^{-3}$	

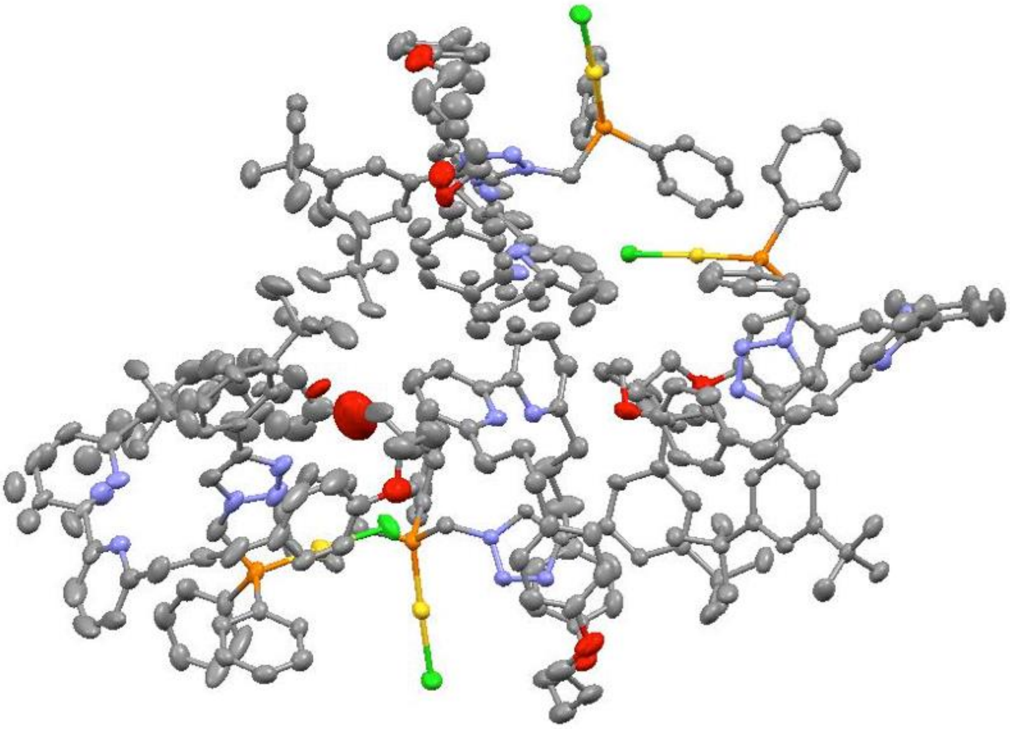

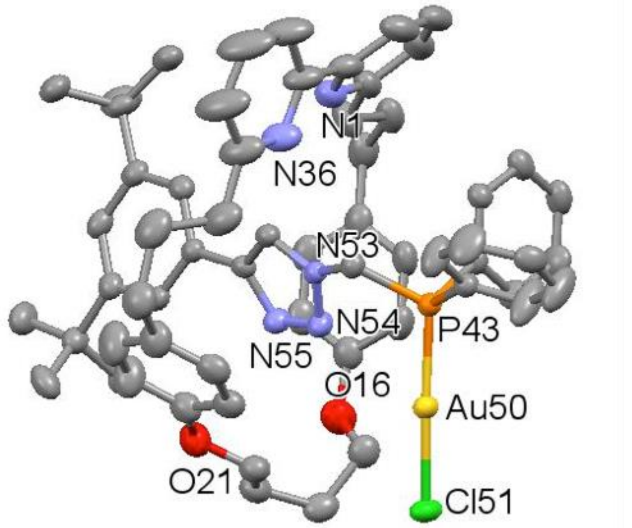

Figure S4. Ellipsoid plot of rotaxane [4AuCl]. Hydrogens were omitted for clarity . Ellipsoids are shown at 50% probability. Bond lengths (Å) and angles (º): P43-Au50 2.234(1), Au50-Cl51 2.295(1); P43-Au50-Cl51 175.50(5). Single crystals suitable for X-ray diffraction were obtained by slow diffusion of hexane into a solution of compound **[4AuCl]** in CH_2Cl_2.

CCDC No.	1406077	
Empirical formula	$C_{244}H_{269}Au_4Cl_4N_{20}O_8P_4$	
Formula weight	4663.32	
Temperature	293(2) K	
Wavelength	0.71073 Å	
Crystal system	Monoclinic	
Space group	P 2/c	
Unit cell dimensions	a = 43.239(3) Å	α = 90°.
	b = 12.4592(9) Å	β = 107.3460(10)°.
	c = 42.915(3) Å	γ = 90°.
Volume	22068(3) $Å^3$	
Z	4	
Density (calculated)	1.404 Mg/m^3	
Absorption coefficient	2.791 mm^{-1}	
F(000)	9524	
Crystal size	0.24 × 0.09 × 0.09 mm^3	
Theta range for data collection	2.356 to 27.562°.	
Index ranges	-56≤h≤56, -16≤k≤16, -55≤l≤52	
Reflections collected	363434	
Independent reflections	50716 [R(int) = 0.0899]	
Completeness to theta = 25.242°	99.9 %	
Absorption correction	Empirical	
Refinement method	Full-matrix least-squares on F^2	
Data / restraints / parameters	50716 / 138 / 3000	
Goodness-of-fit on F^2	1.016	
Final R indices [I>2sigma(I)]	R_1 = 0.0439, wR_2 = 0.1052	
R indices (all data)	R_1 = 0.0752, wR_2 = 0.1199	
Largest diff. peak and hole	2.444 and -2.133 e.$Å^{-3}$	

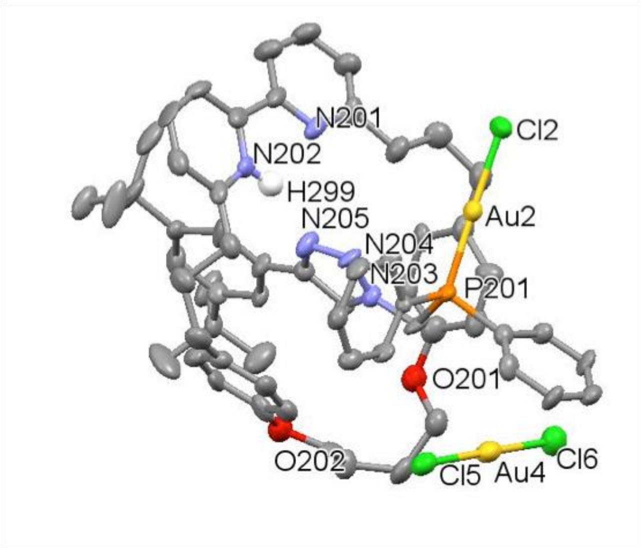

Figure S5. Ellipsoid plot of rotaxane [4AuCl.H]$AuCl_2$. Hydrogens were omitted for clarity. Ellipsoids are shown at 50% probability. Bond lengths (Å) and angles (°): P201-Au2 2.230(4), Au2-Cl2 2.279(4), Cl5-Au4 2.268(6), Au4-Cl6 2.271(6), N202···H299 0.86, H299···N205 2.08, N205···N202 2.90(2); Cl5-Au4-Cl6 179.2(2), P201-Au2-Cl2 176.5(2), N202···H299···N205 157.7. Single crystals suitable for X-ray diffraction were obtained by slow diffusion of hexane into a solution of **[4AuCl.H]$AuCl_2$** in CH_2Cl_2. The hydrogen atoms of the protonated bipyridines were observed as peaks of electron density, however they could not be freely refined and thus were placed in calculated positions.

CCDC No.	1406074	
Empirical formula	$C_{122}H_{135}Au_4Cl_6N_{10}O_4P_2$	
Formula weight	2867.90	
Temperature	293(2) K	
Wavelength	0.71073 Å	
Crystal system	Triclinic	
Space group	P-1	
Unit cell dimensions	a = 12.9773(9) Å	α = 74.345(5)°.
	b = 18.3295(13) Å	β = 82.855(6)°.
	c = 25.1964(18) Å	γ = 88.471(6)°.
Volume	5726.1(7) $Å^3$	
Z	2	
Density (calculated)	1.663 Mg/m^3	
Absorption coefficient	5.334 mm^{-1}	
F(000)	2834	
Crystal size	0.20 × 0.20 × 0.20 mm^3	
Theta range for data collection	3.032 to 27.484°.	
Index ranges	-16≤h≤16, -23≤k≤23, -32≤l≤32	
Reflections collected	95325	
Independent reflections	26118 [R(int) = 0.1330]	
Completeness to theta = 25.242°	99.7 %	
Refinement method	Full-matrix least-squares on F^2	
Data / restraints / parameters	26118 / 12 / 1345	
Goodness-of-fit on F^2	1.020	
Final R indices [I>2sigma(I)]	R_1 = 0.1008, wR_2 = 0.2451	
R indices (all data)	R_1 = 0.1685, wR_2 = 0.2810	
Largest diff. peak and hole	9.135 and -3.554 e.$Å^{-3}$	

4 NMR Data for Novel Compounds

Rotaxane S1 ^{1}H NMR ($CDCl_3$, 500 MHz, 300 K)

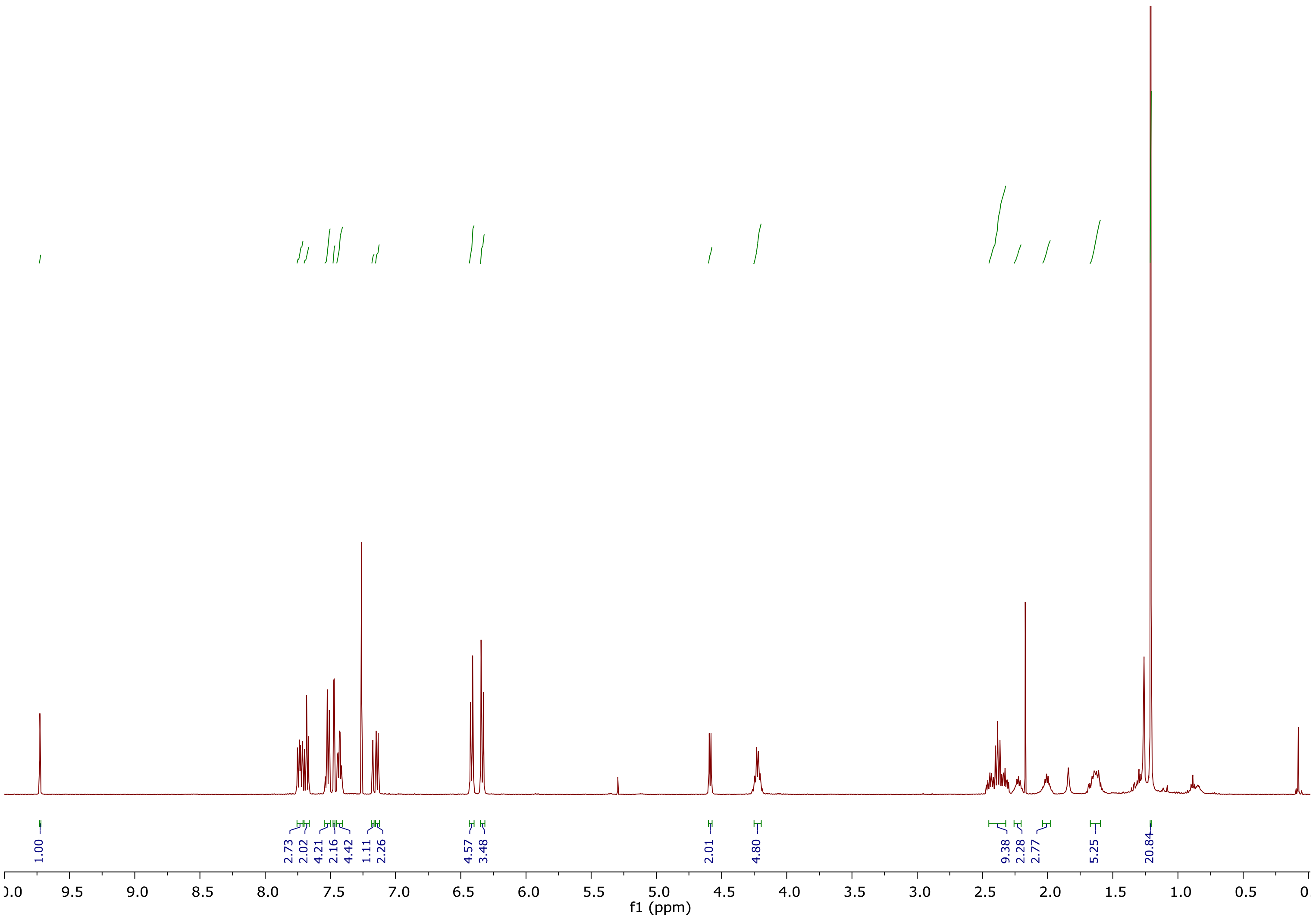

Rotaxane S1 ^{31}P NMR ($CDCl_3$, 202 MHz, 300 K)

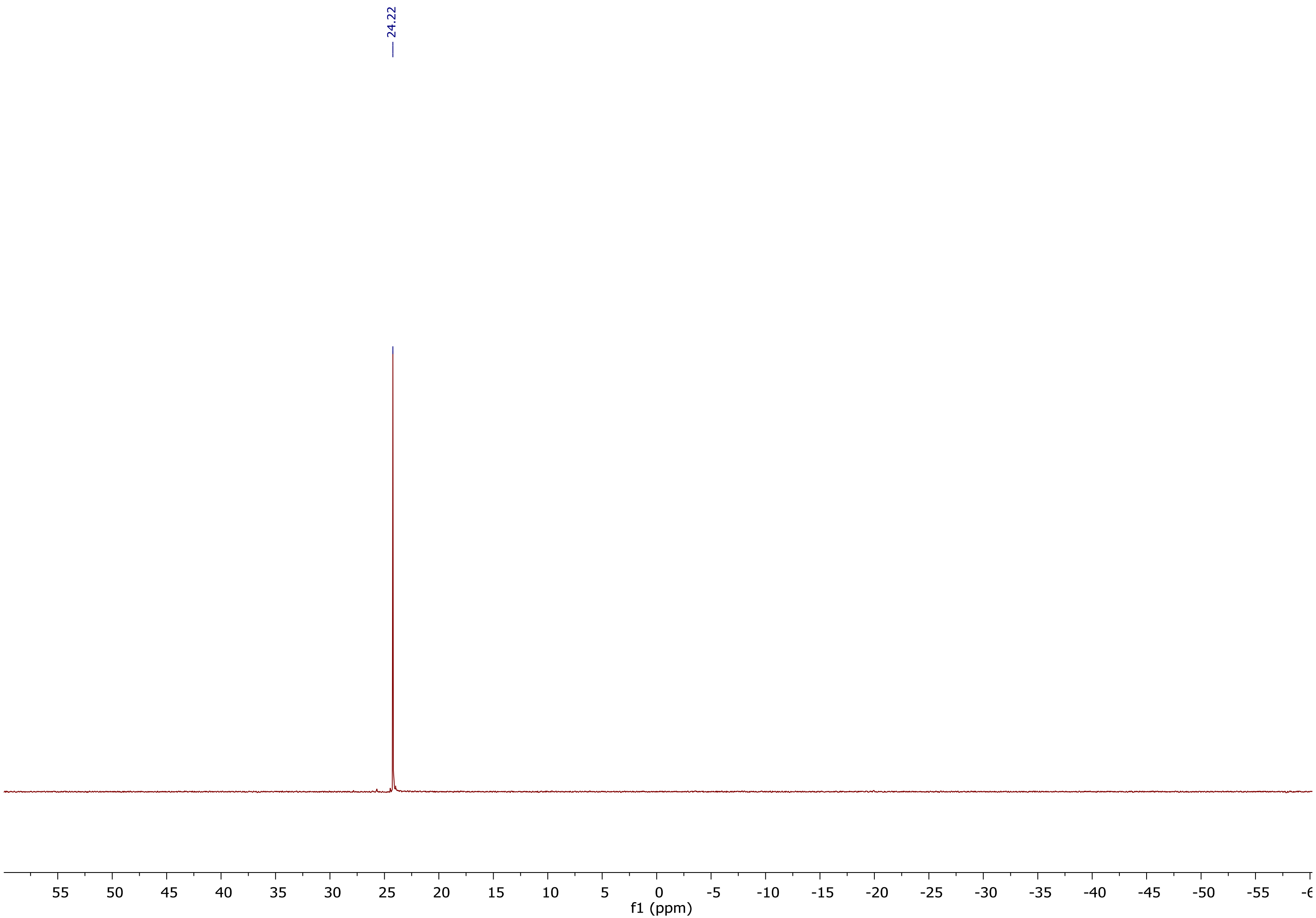

Rotaxane S1 ^{13}C NMR ($CDCl_3$, 125 MHz, 300 K)

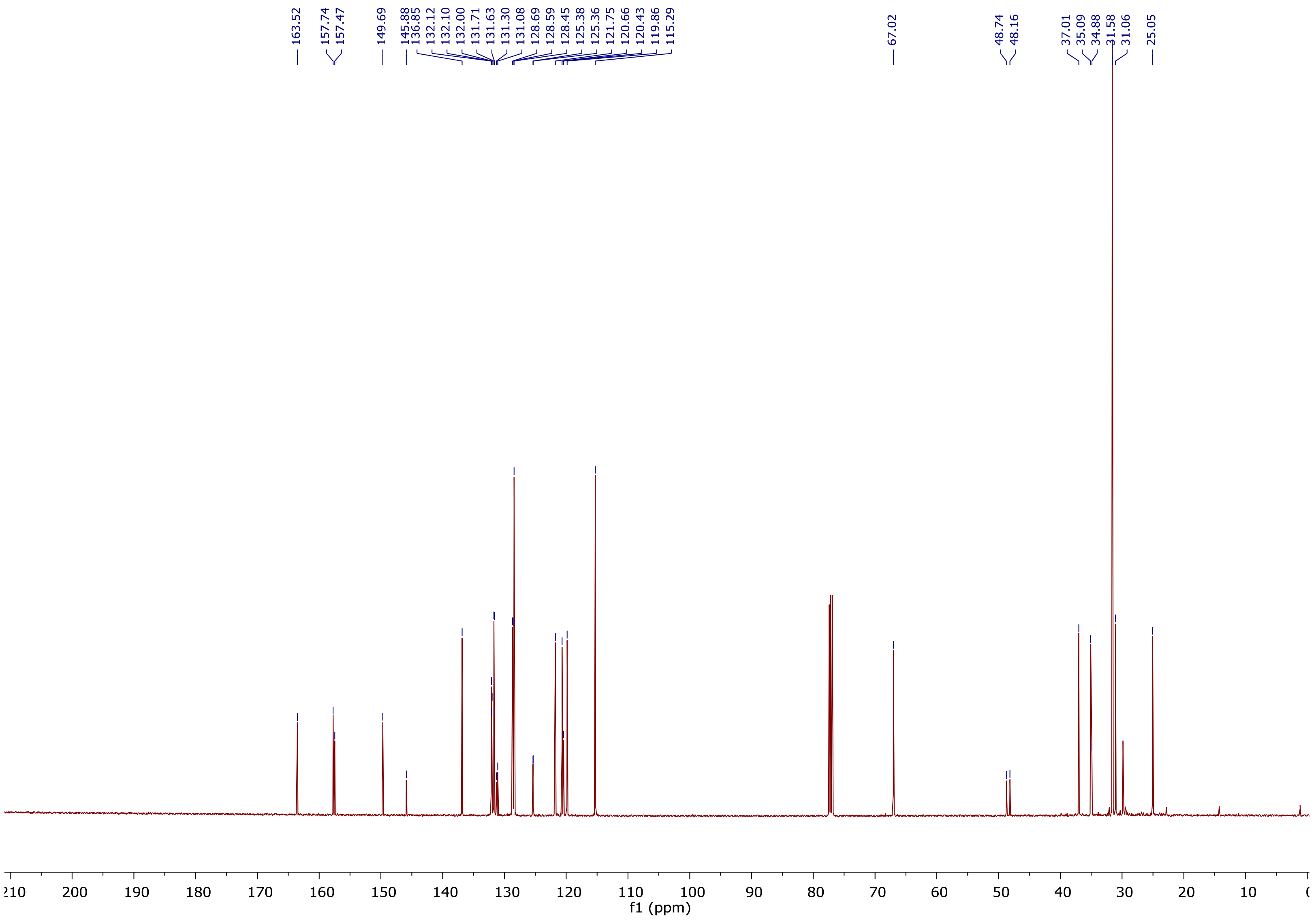

Rotaxane S1 COSY ($CDCl_3$, 500 MHz, 300 K)

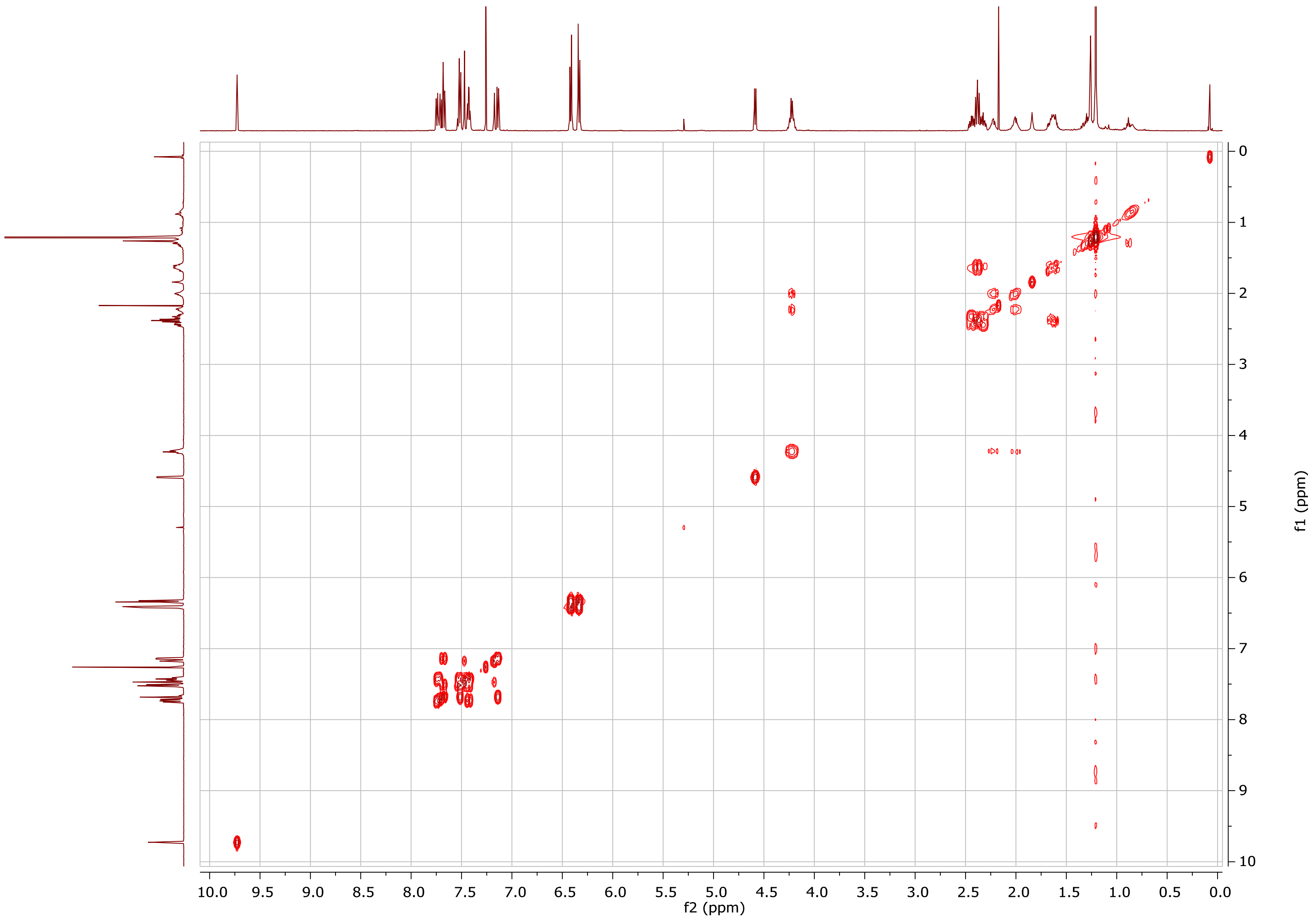

Rotaxane S1 HSQC ($CDCl_3$, 500 MHz, 300 K)

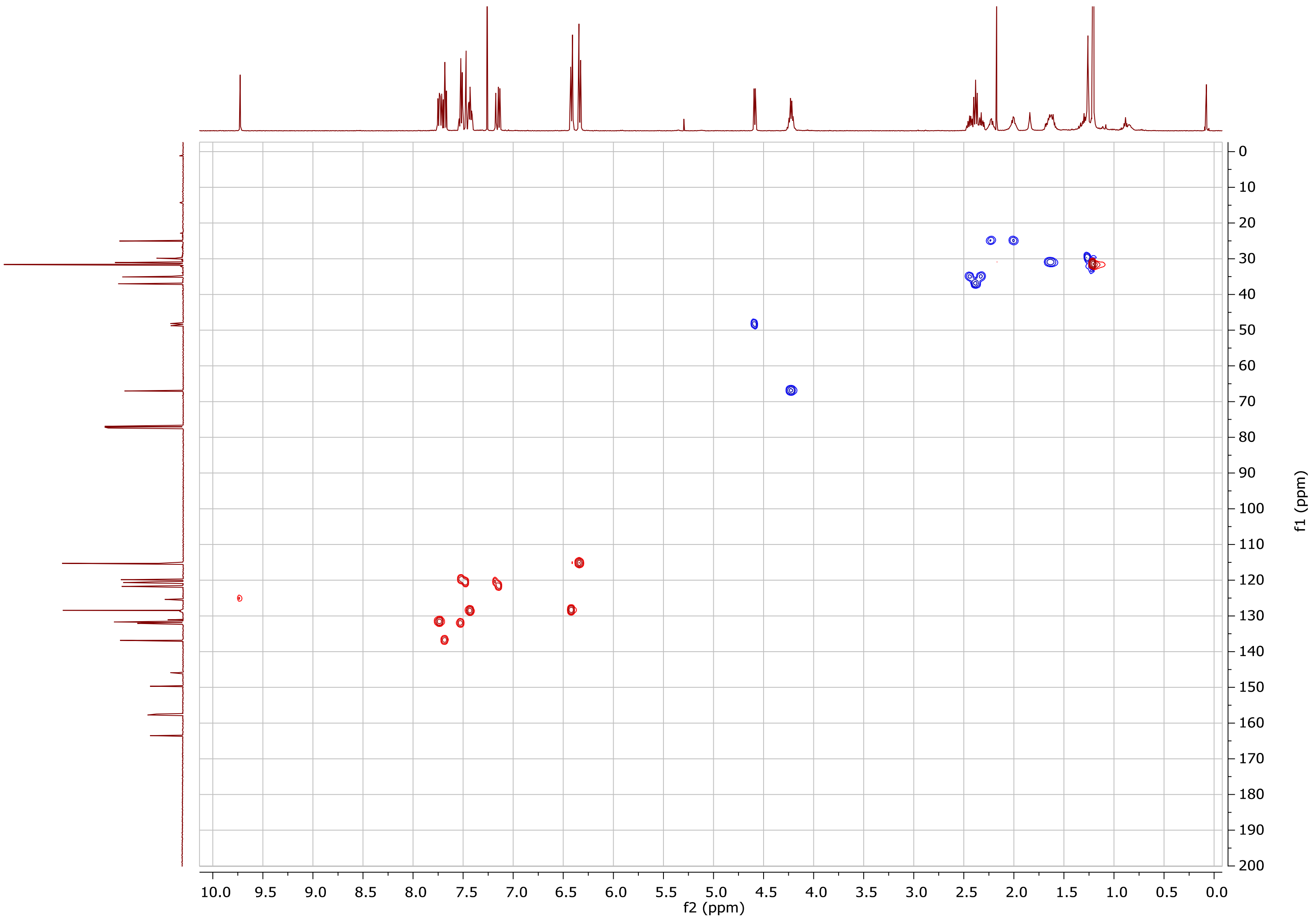

Rotaxane S1 HMBC ($CDCl_3$, 500 MHz, 300 K)

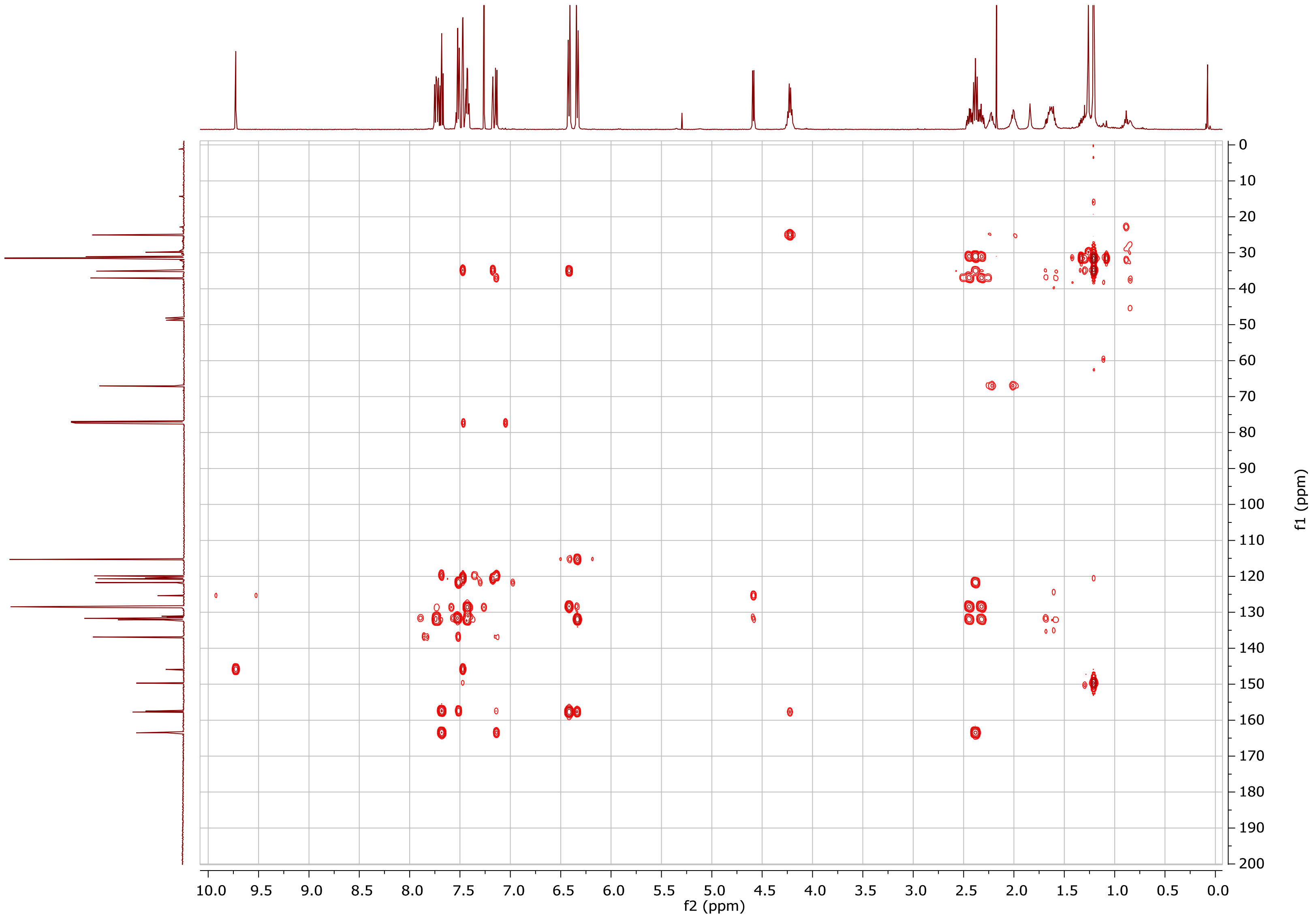

Rotaxane 4 ^{1}H NMR ($CDCl_3$, 500 MHz, 300 K)

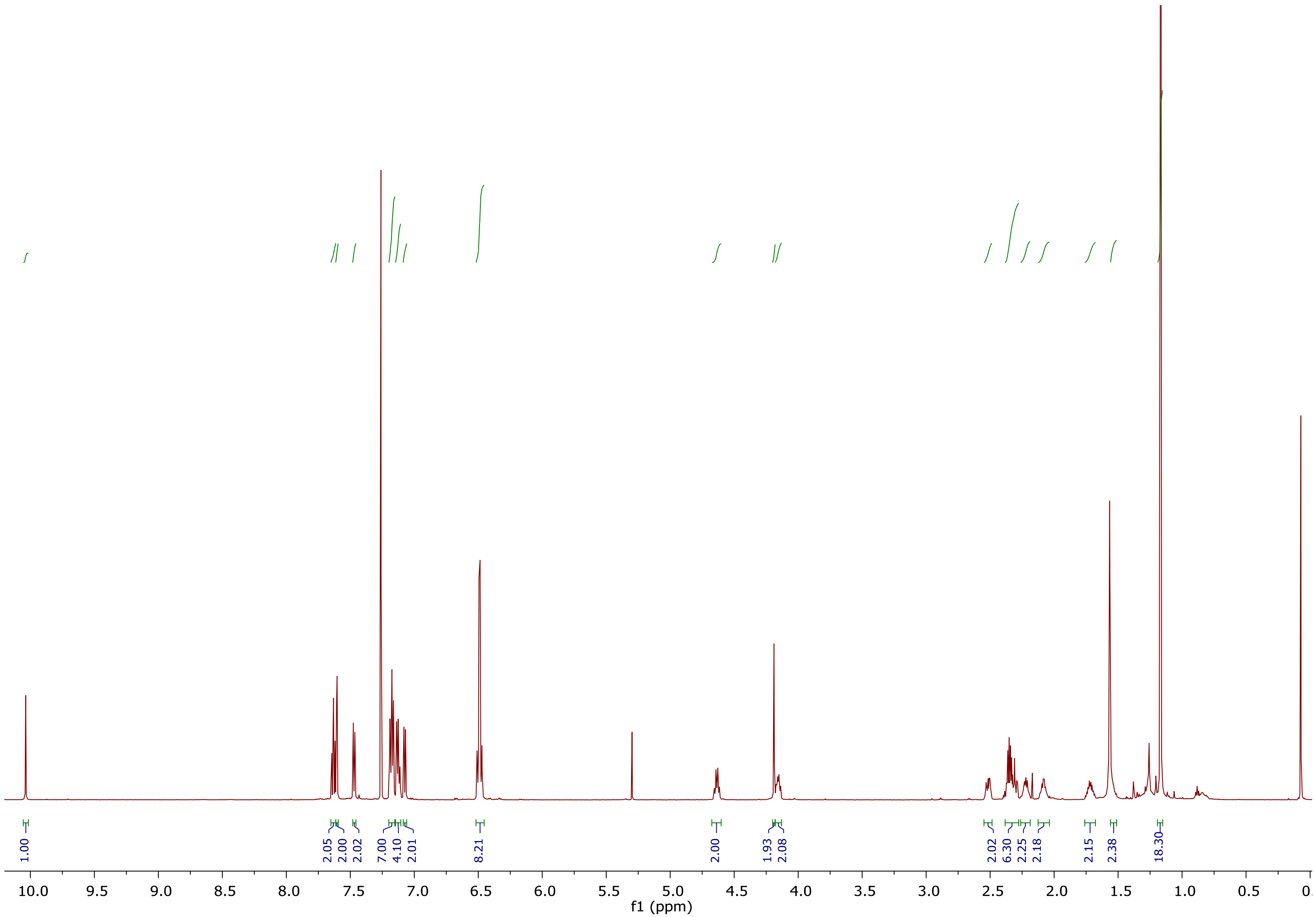

Rotaxane 4 ^{31}P NMR ($CDCl_3$, 202 MHz, 300 K)

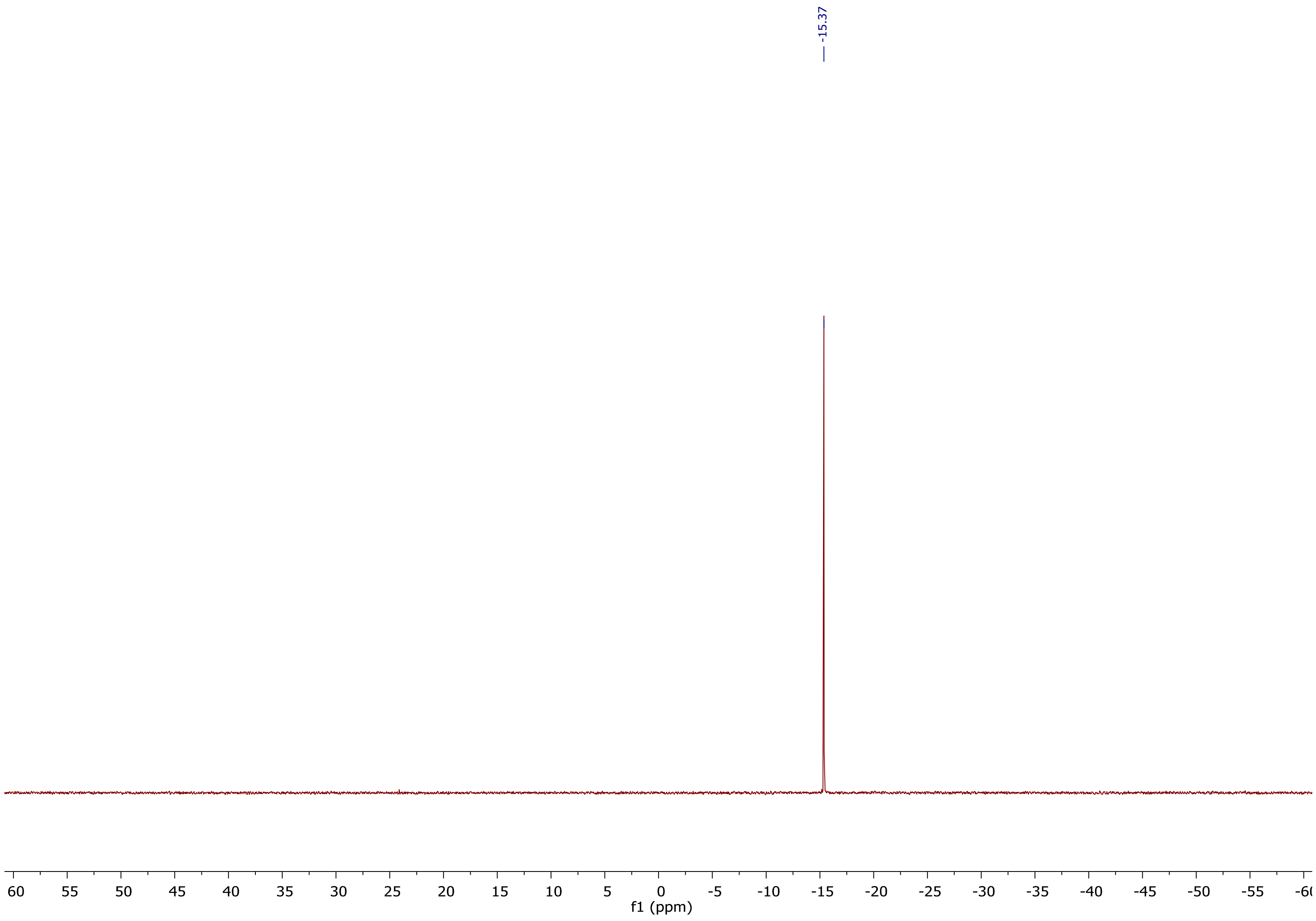

Rotaxane 4 ^{13}C NMR ($CDCl_3$, 125 MHz, 300 K)

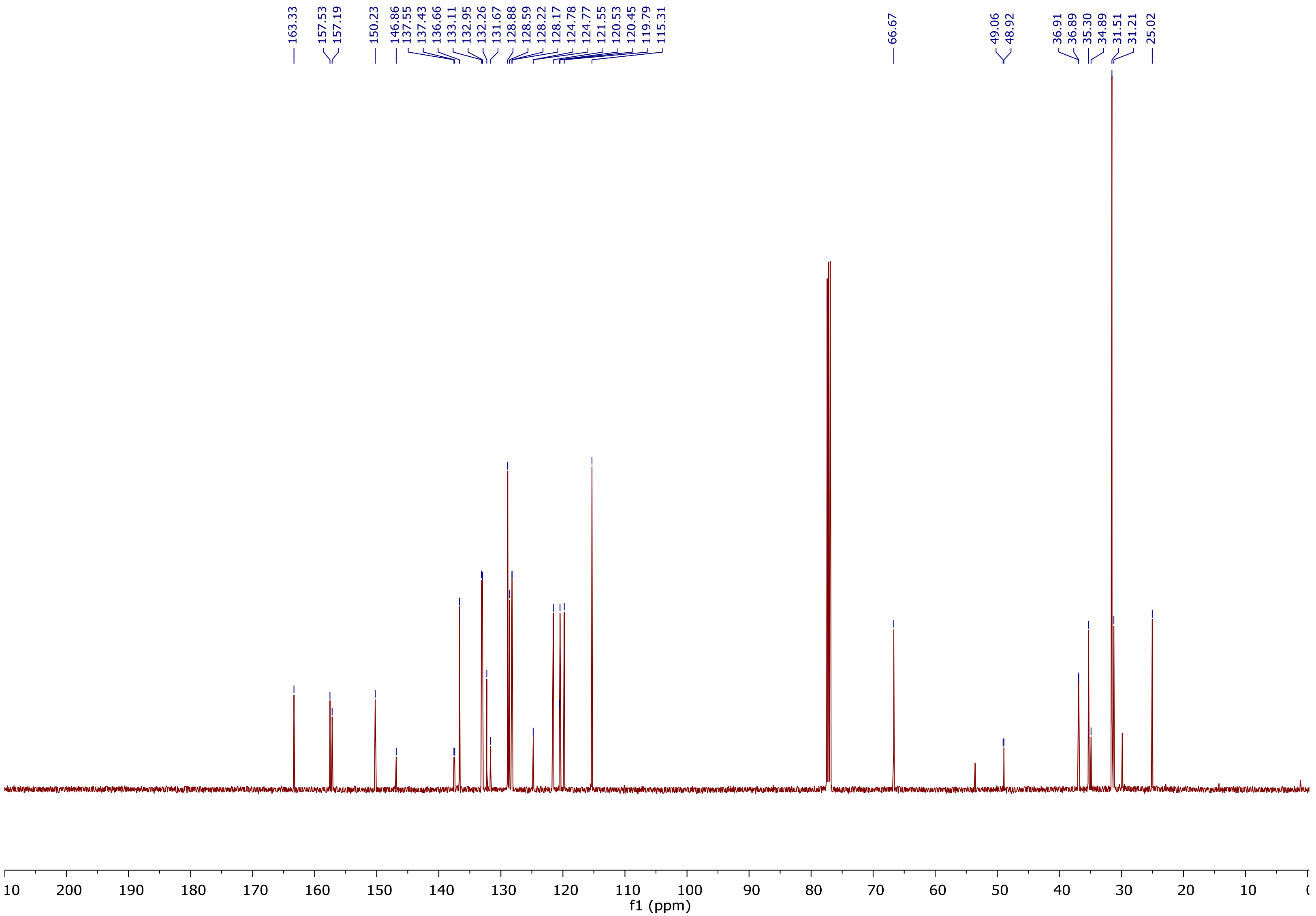

Rotaxane 4 COSY ($CDCl_3$, 500 MHz, 300 K)

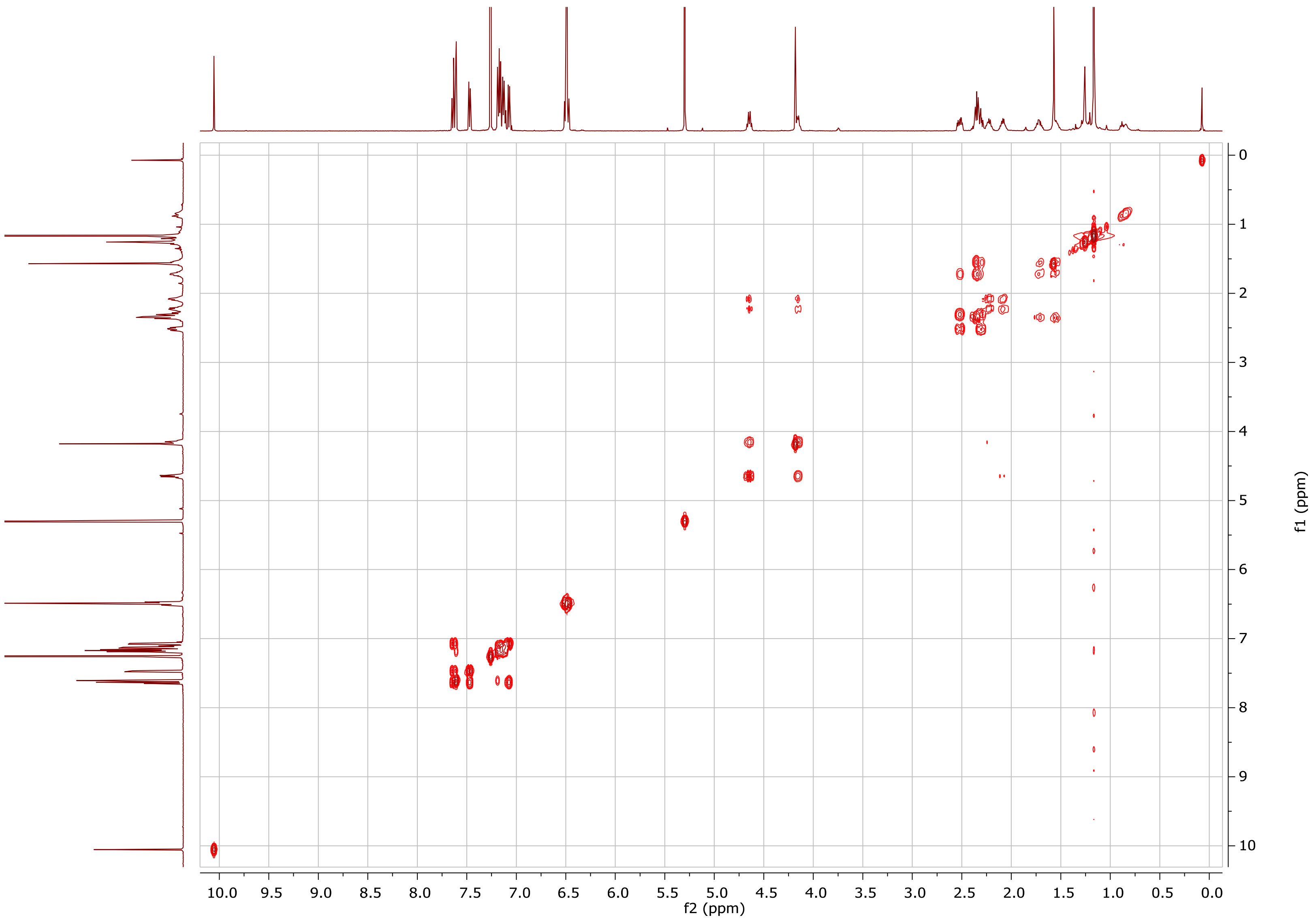

Rotaxane 4 HSQC ($CDCl_3$, 500 MHz, 300 K)

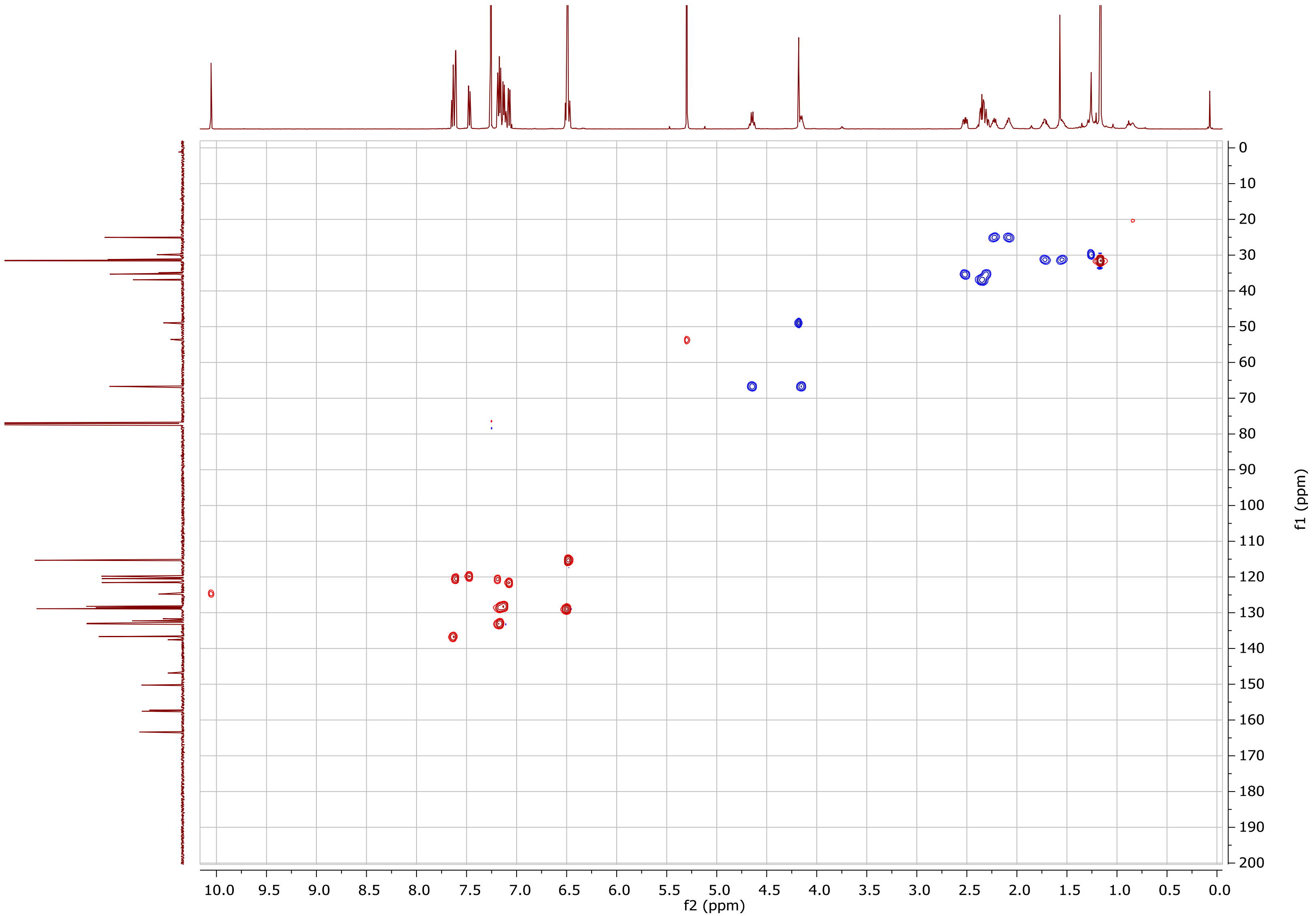

Rotaxane 4 HMBC ($CDCl_3$, 500 MHz, 300 K)

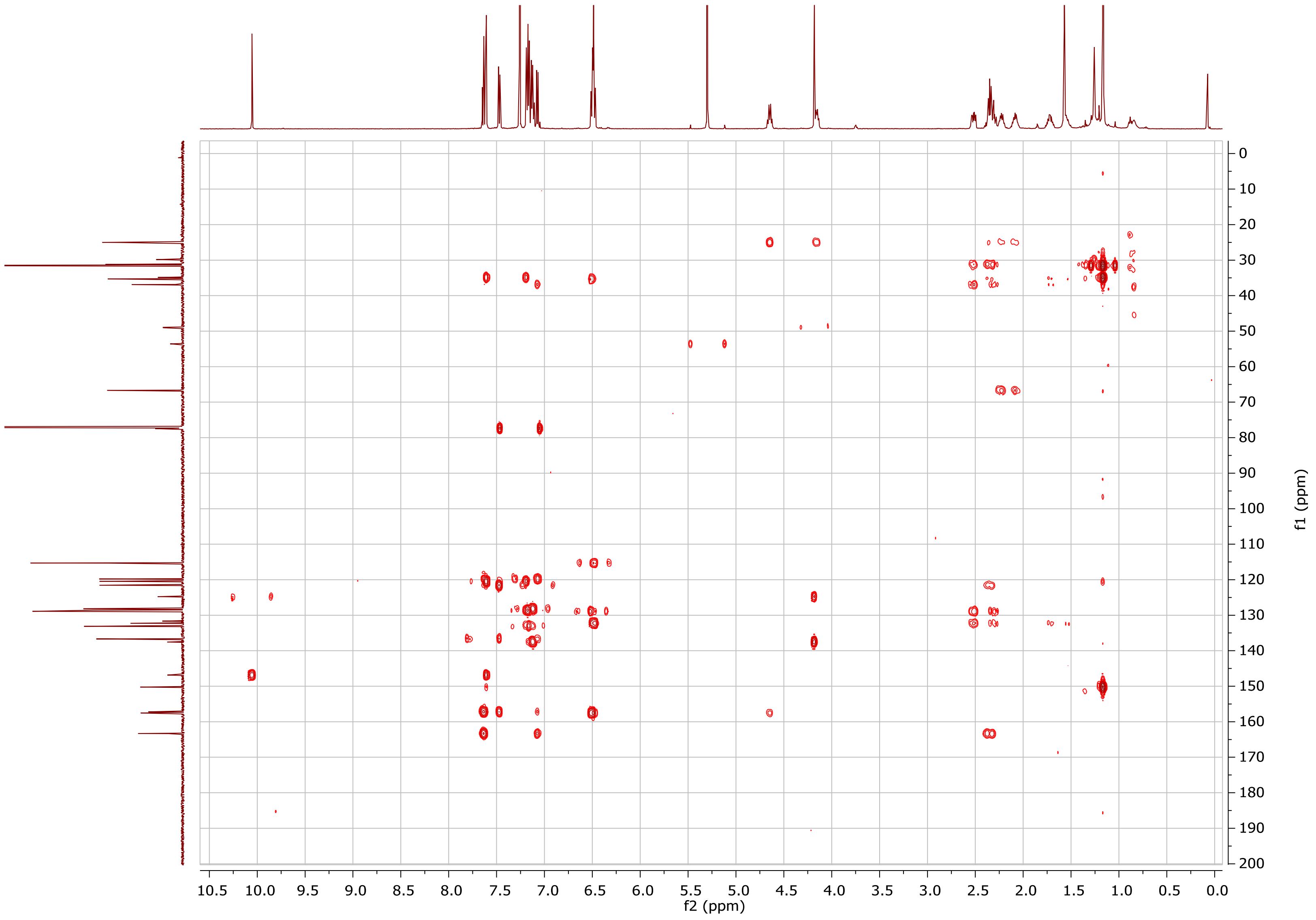

Rotaxane [4AuCl] ^{1}H NMR ($CDCl_3$, 600 MHz, 300 K)

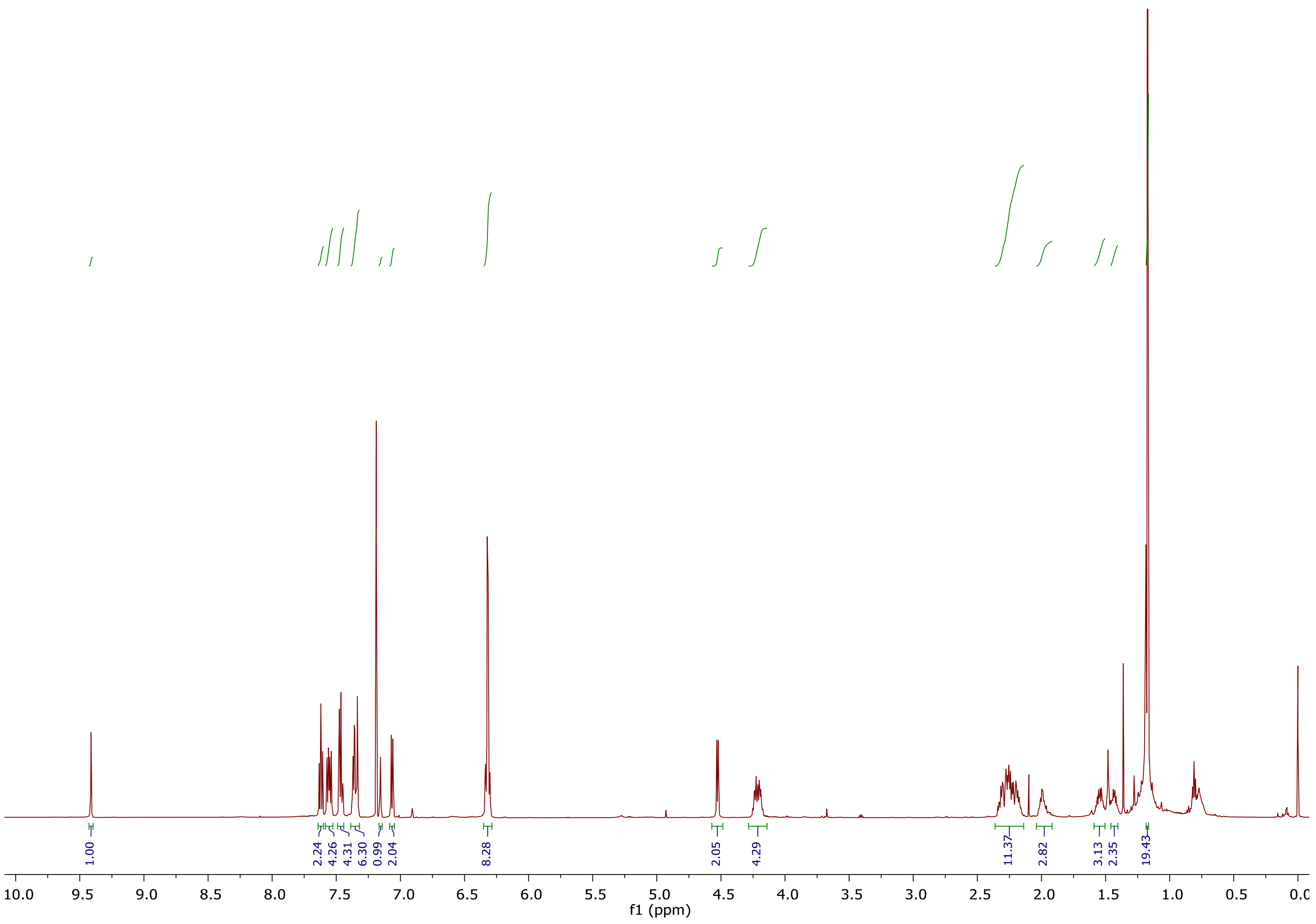

Rotaxane [4AuCl] ^{31}P NMR ($CDCl_3$, 240 MHz, 300 K)

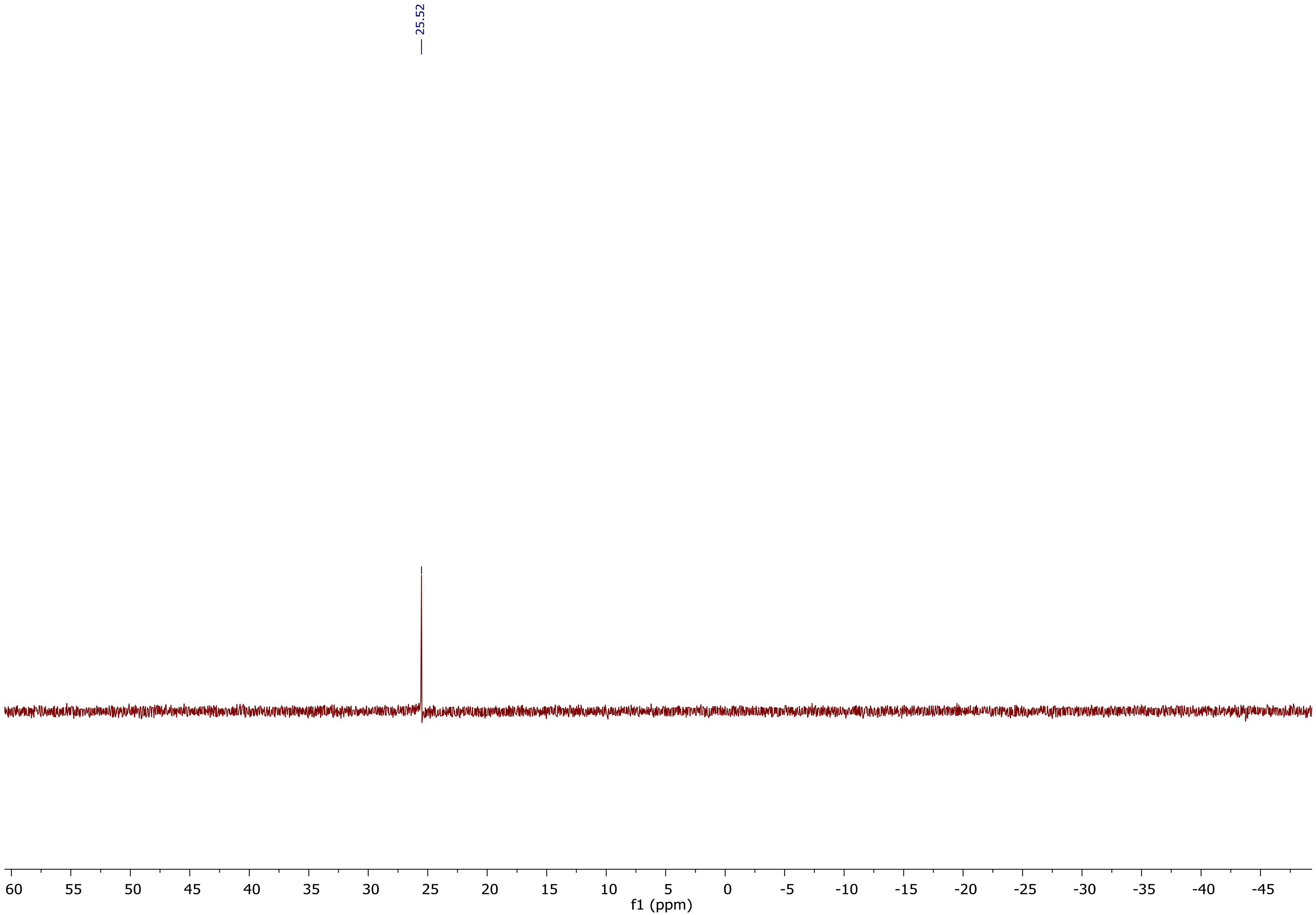

Rotaxane [4AuCl] ^{13}C NMR ($CDCl_3$, 125 MHz, 300 K)

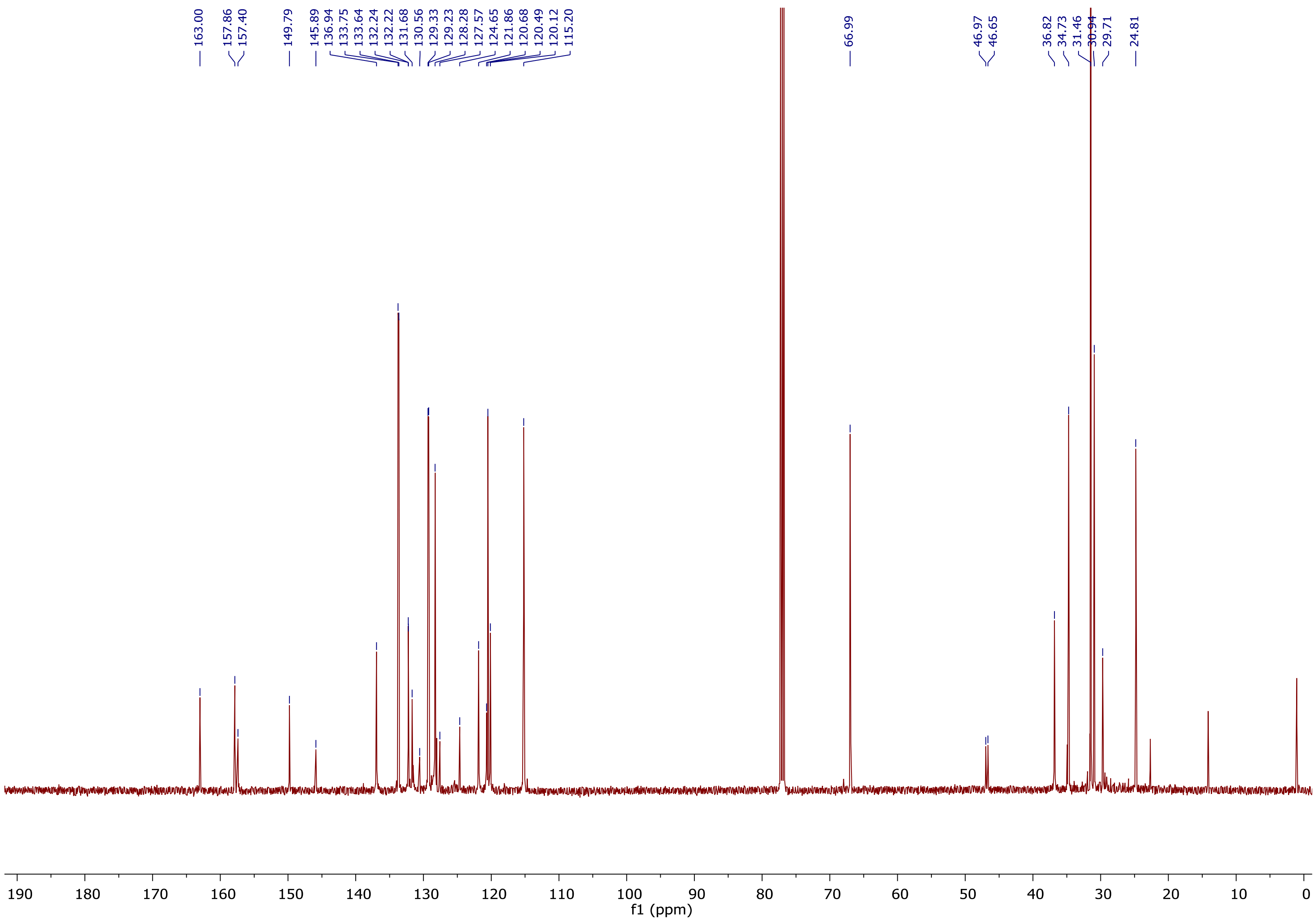

Rotaxane [4AuCl] COSY ($CDCl_3$, 500 MHz, 300 K)

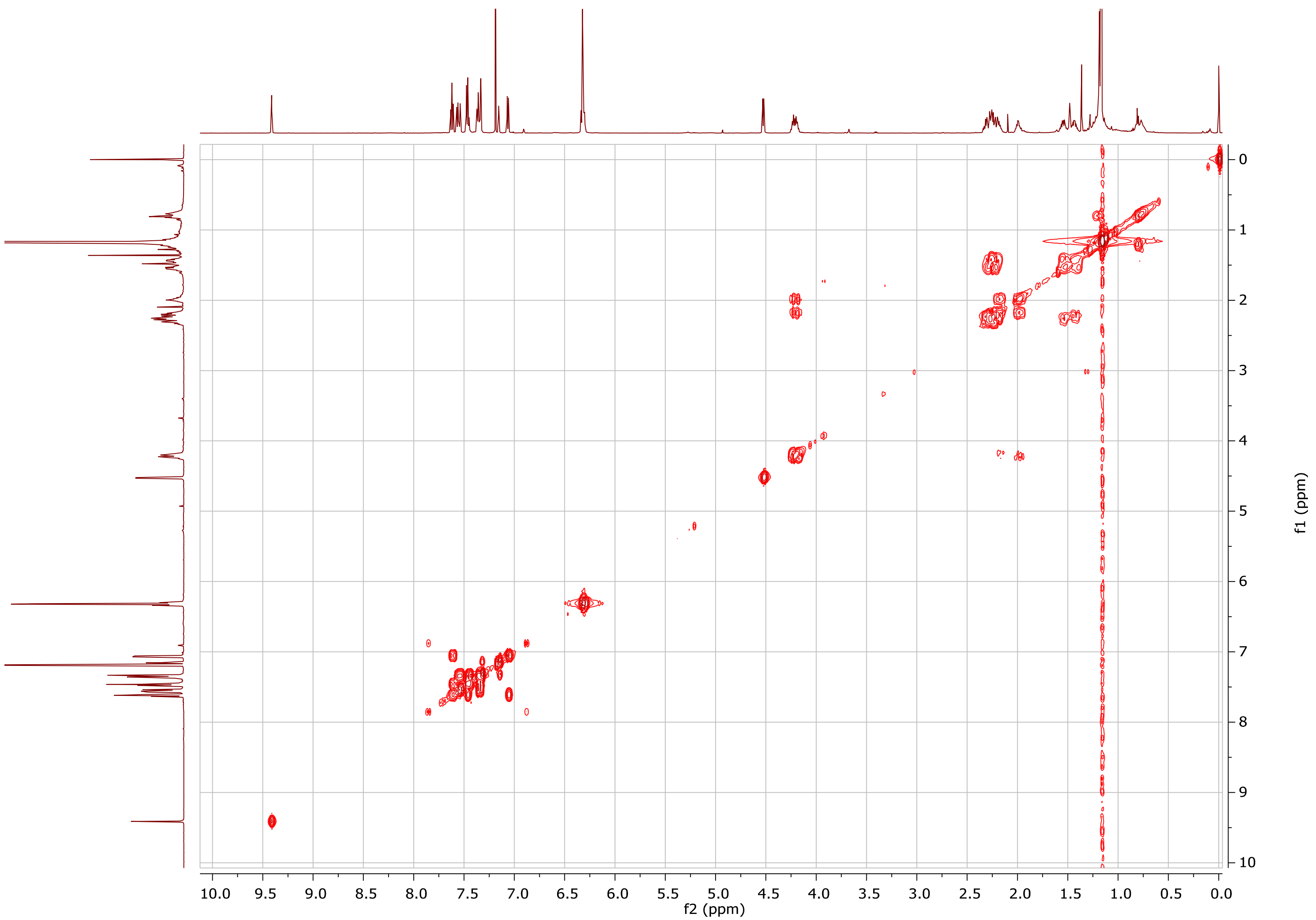

Rotaxane [4AuCl] HSQC ($CDCl_3$, 500 MHz, 300 K)

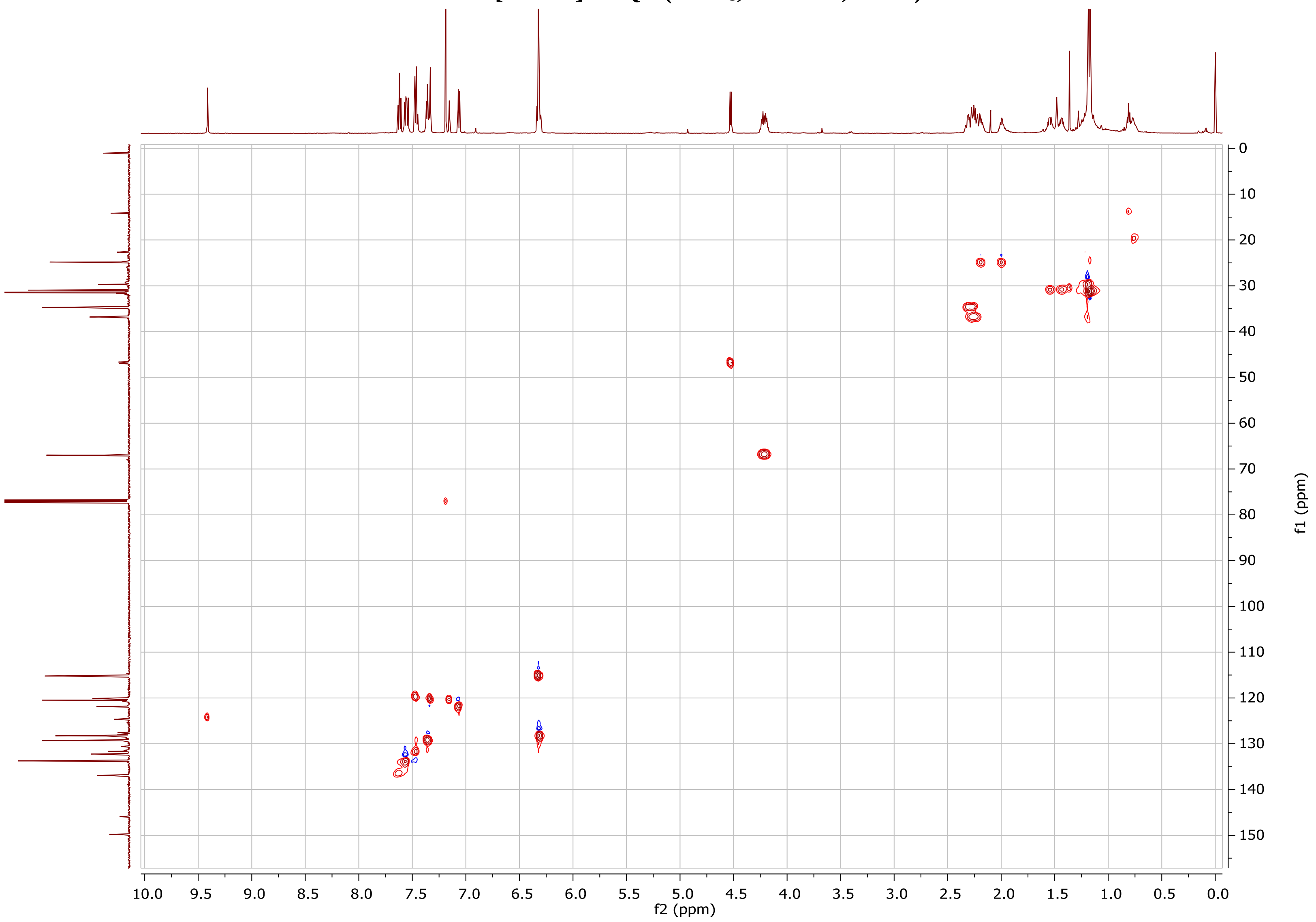

Rotaxane [4AuCl] HMBC ($CDCl_3$, 500 MHz, 300 K)

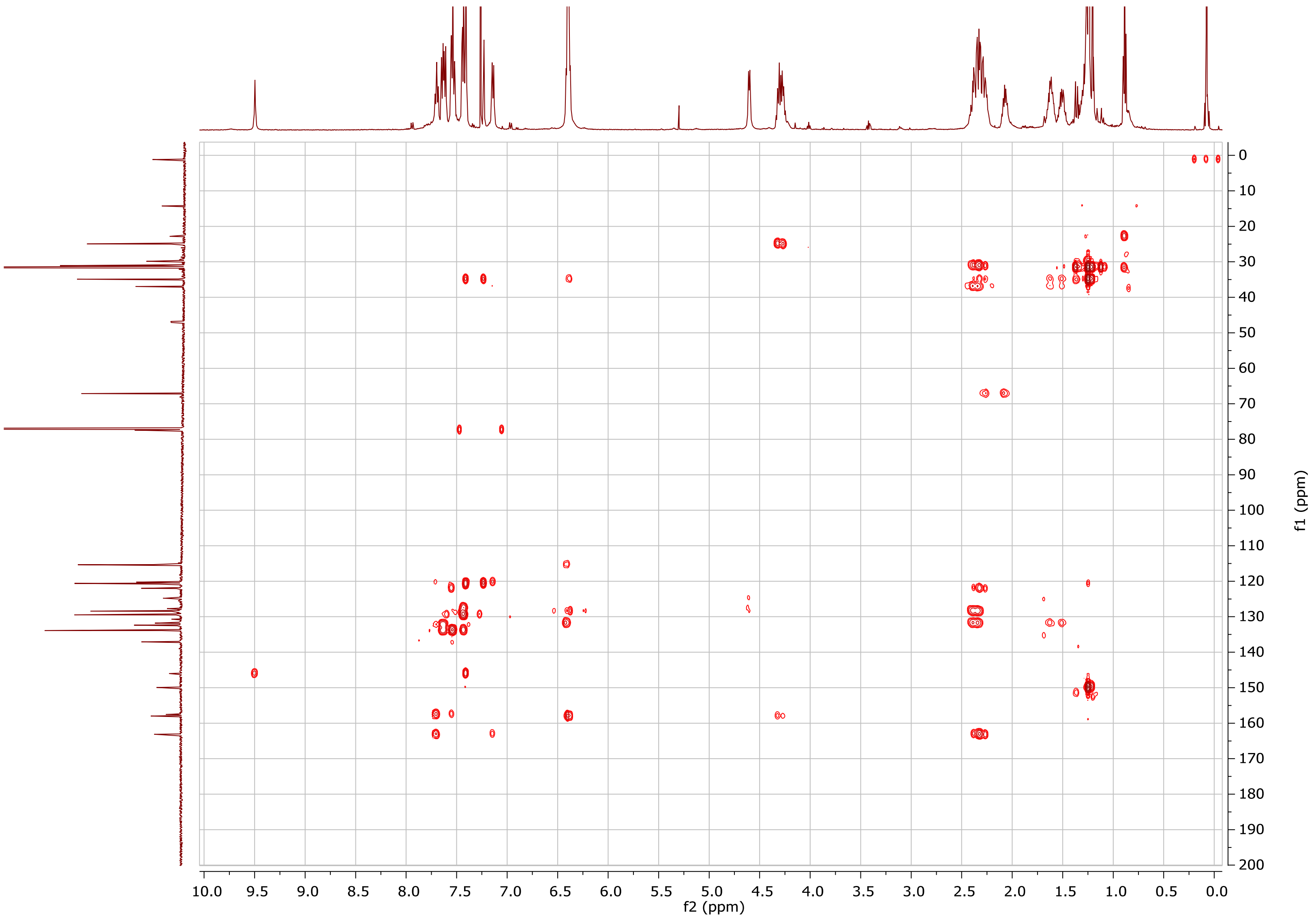

Thread S2 ^{1}H NMR ($CDCl_3$, 600 MHz, 300 K)

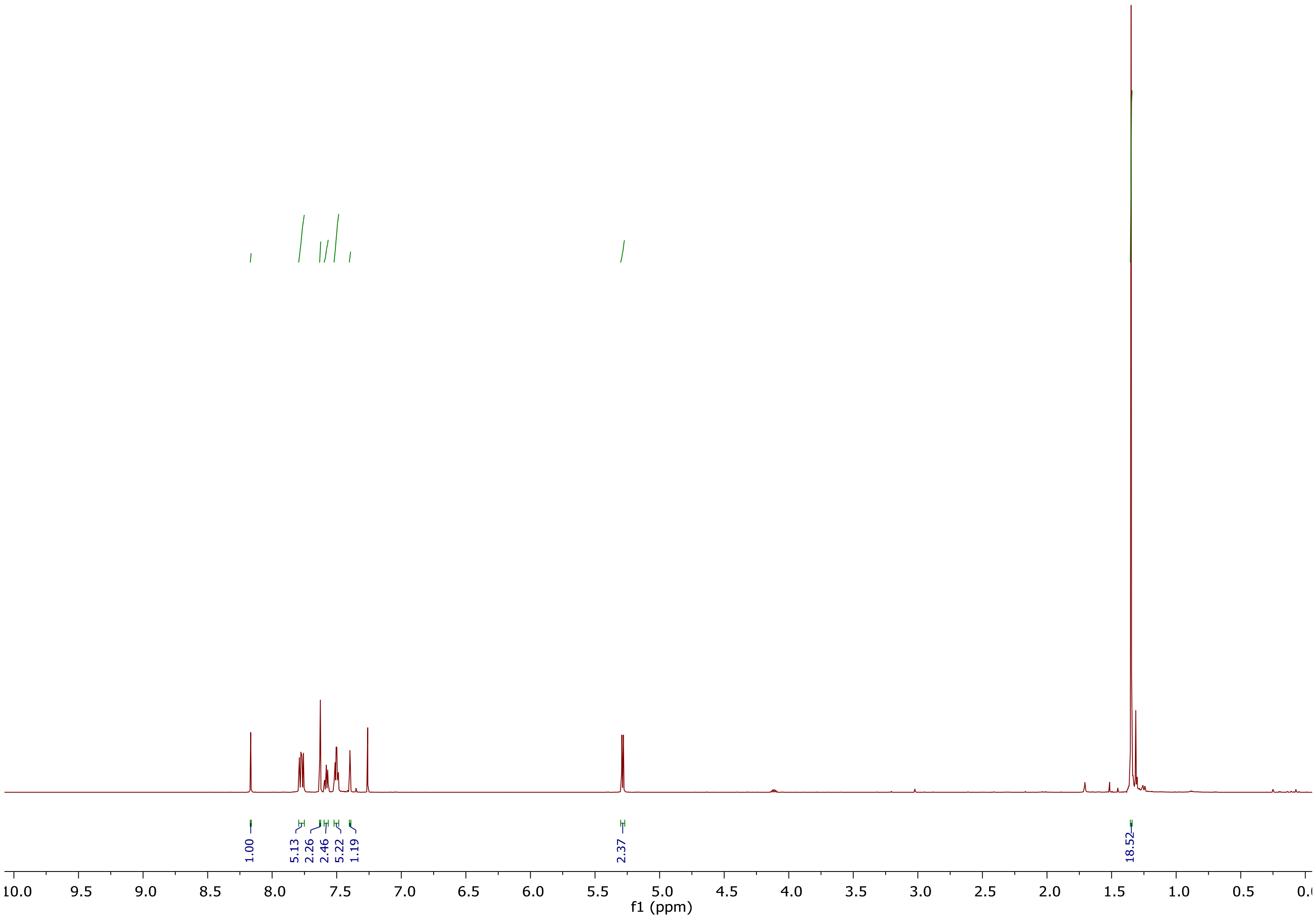

Thread S2 ^{31}P NMR ($CDCl_3$, 240 MHz, 300 K)

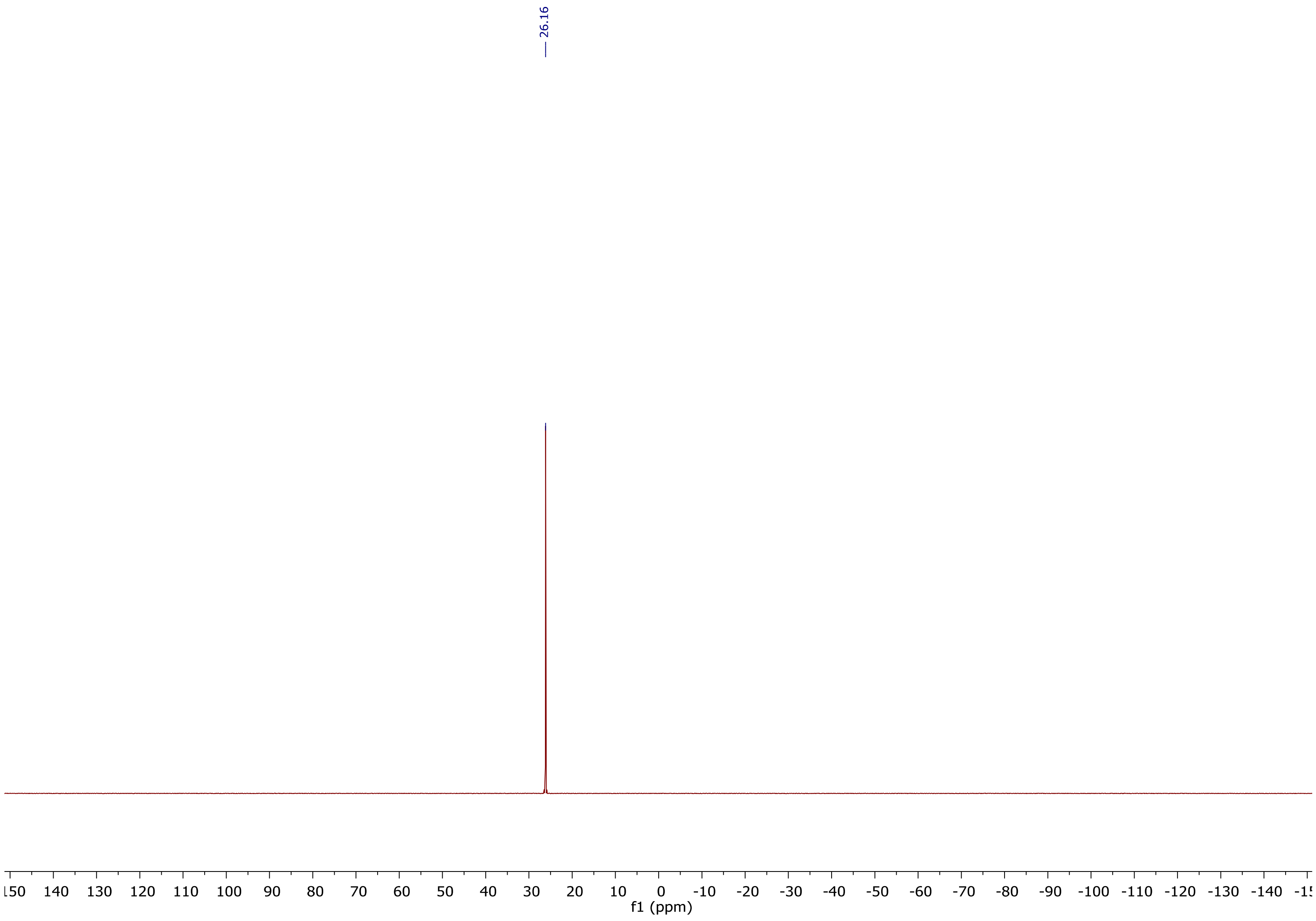

Thread S2 ^{13}C NMR ($CDCl_3$, 151 MHz, 300 K)

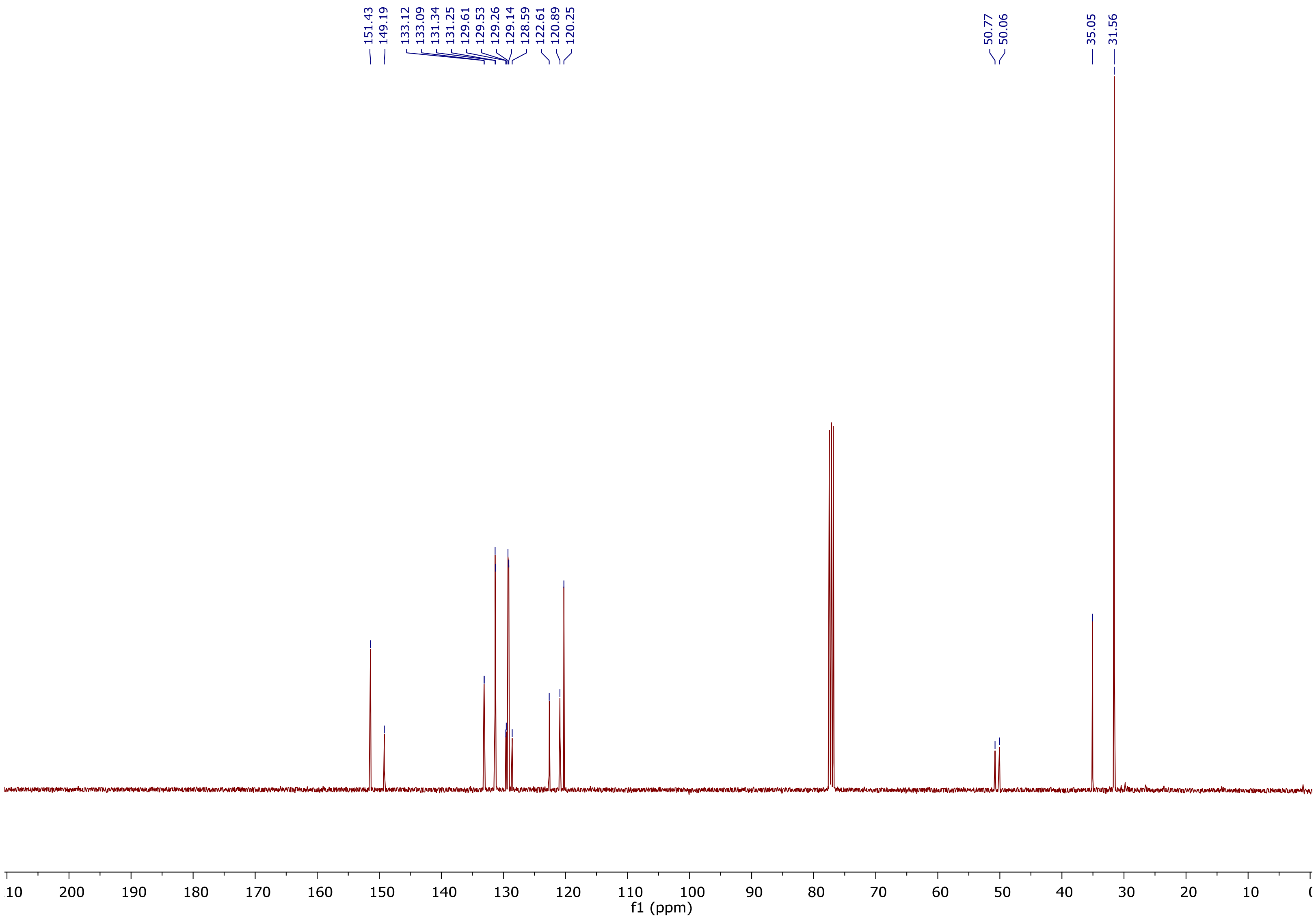

Thread S2 COSY ($CDCl_3$, 600 MHz, 300 K)

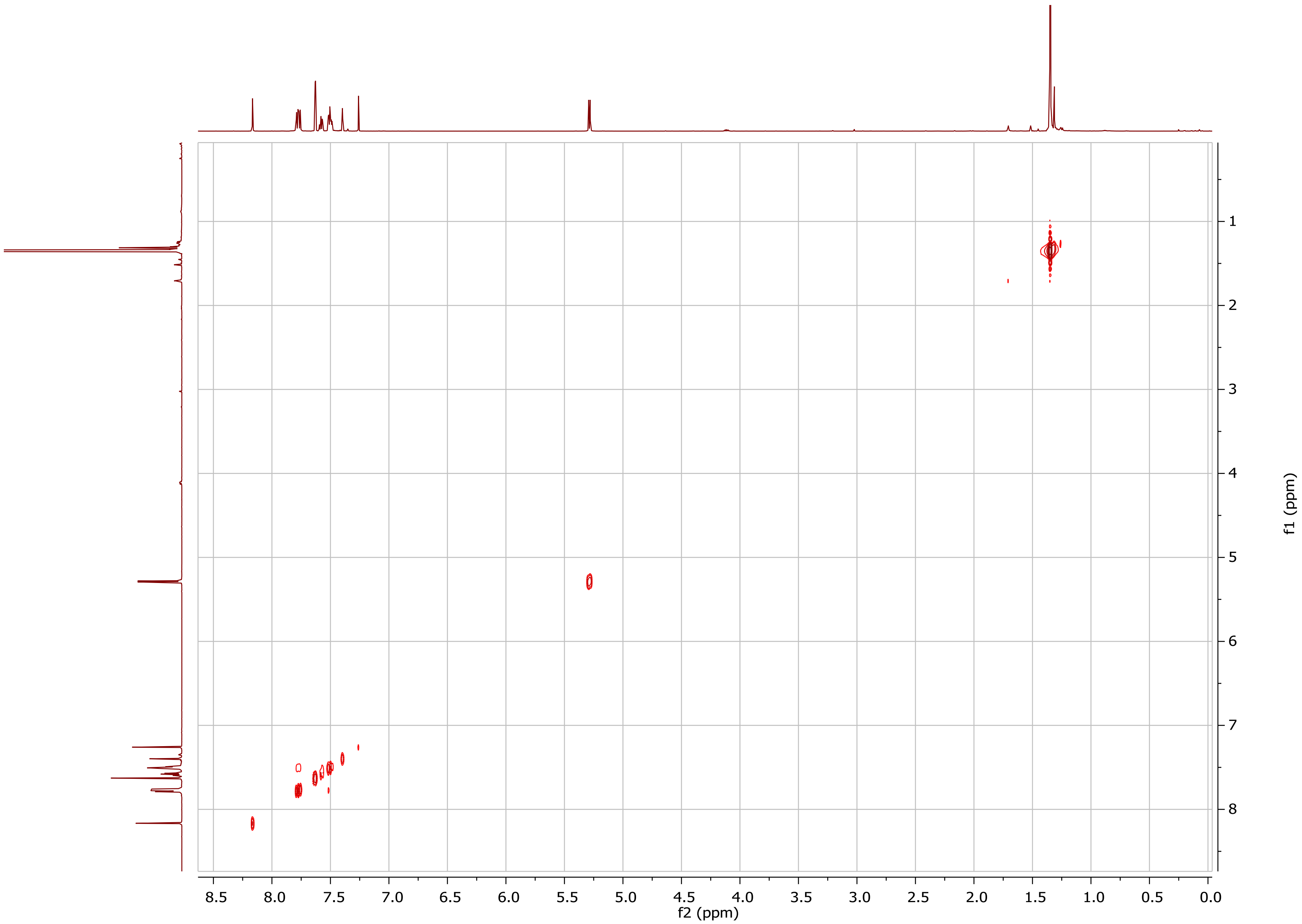

Thread S2 HSQC ($CDCl_3$, 600 MHz, 300 K)

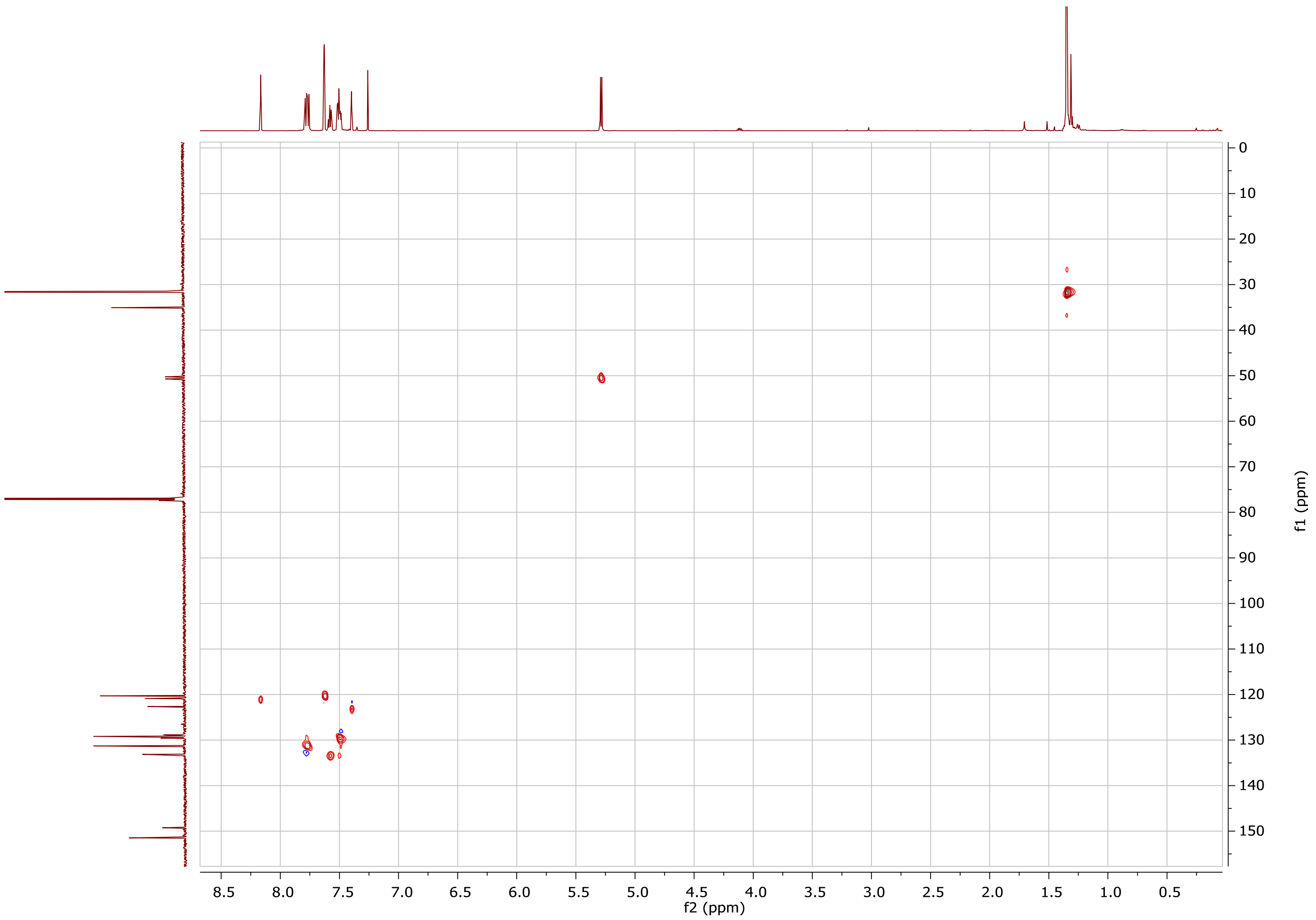

Thread S2 HMBC ($CDCl_3$, 600 MHz, 300 K)

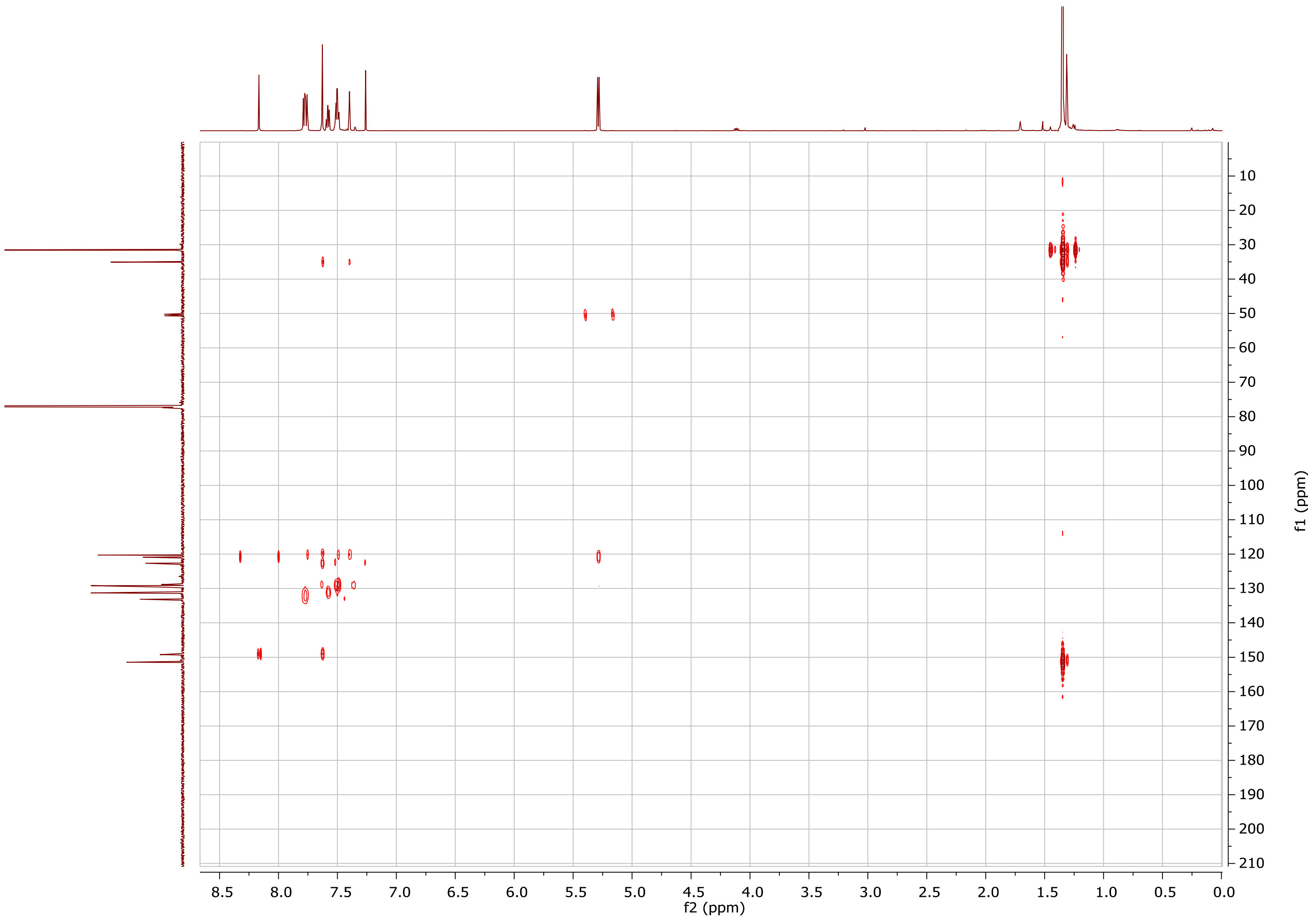

Thread 5 ^{1}H NMR ($CDCl_3$, 600 MHz, 300 K)

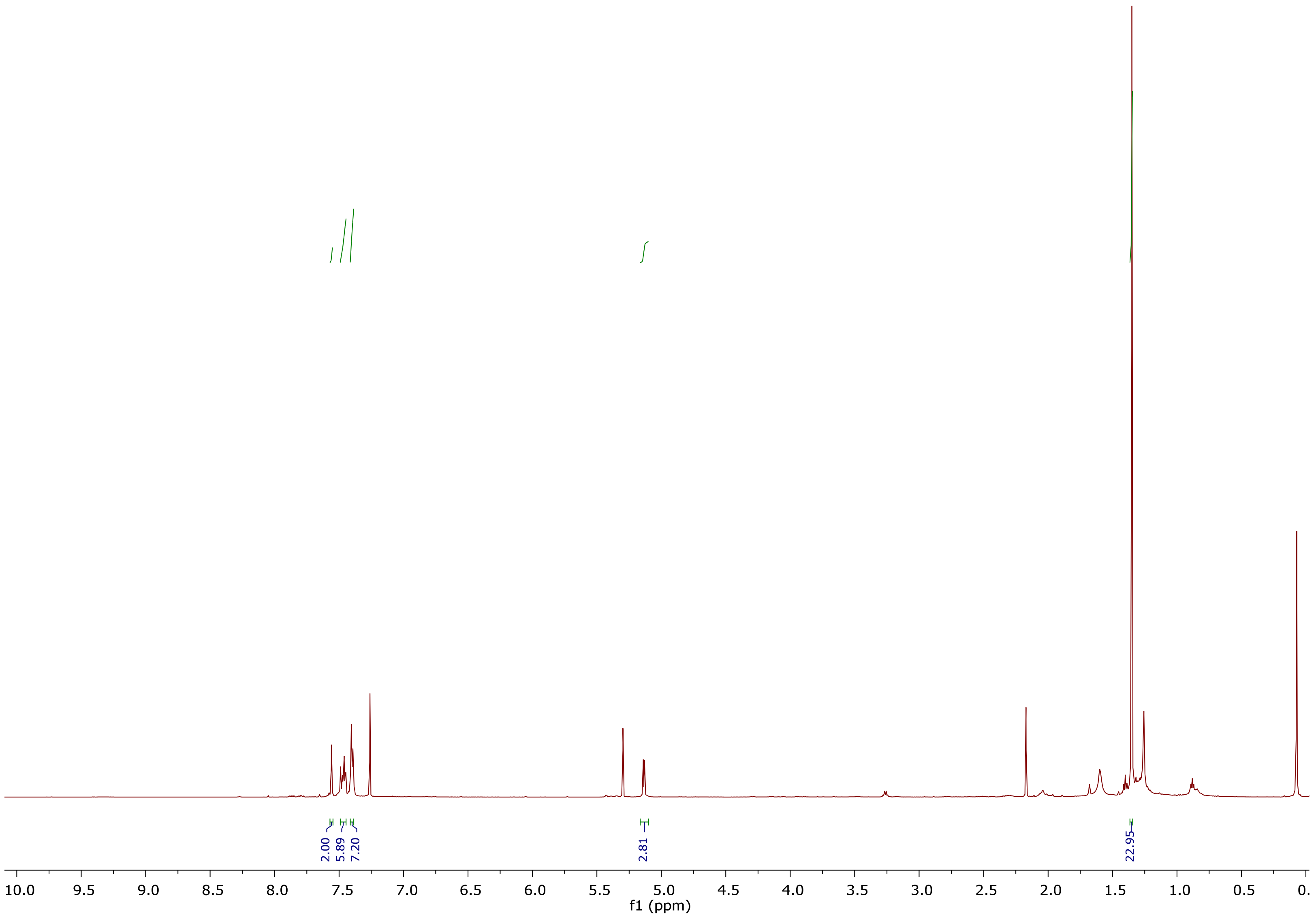

Thread 5 ^{31}P NMR ($CDCl_3$, 240 MHz, 300 K)

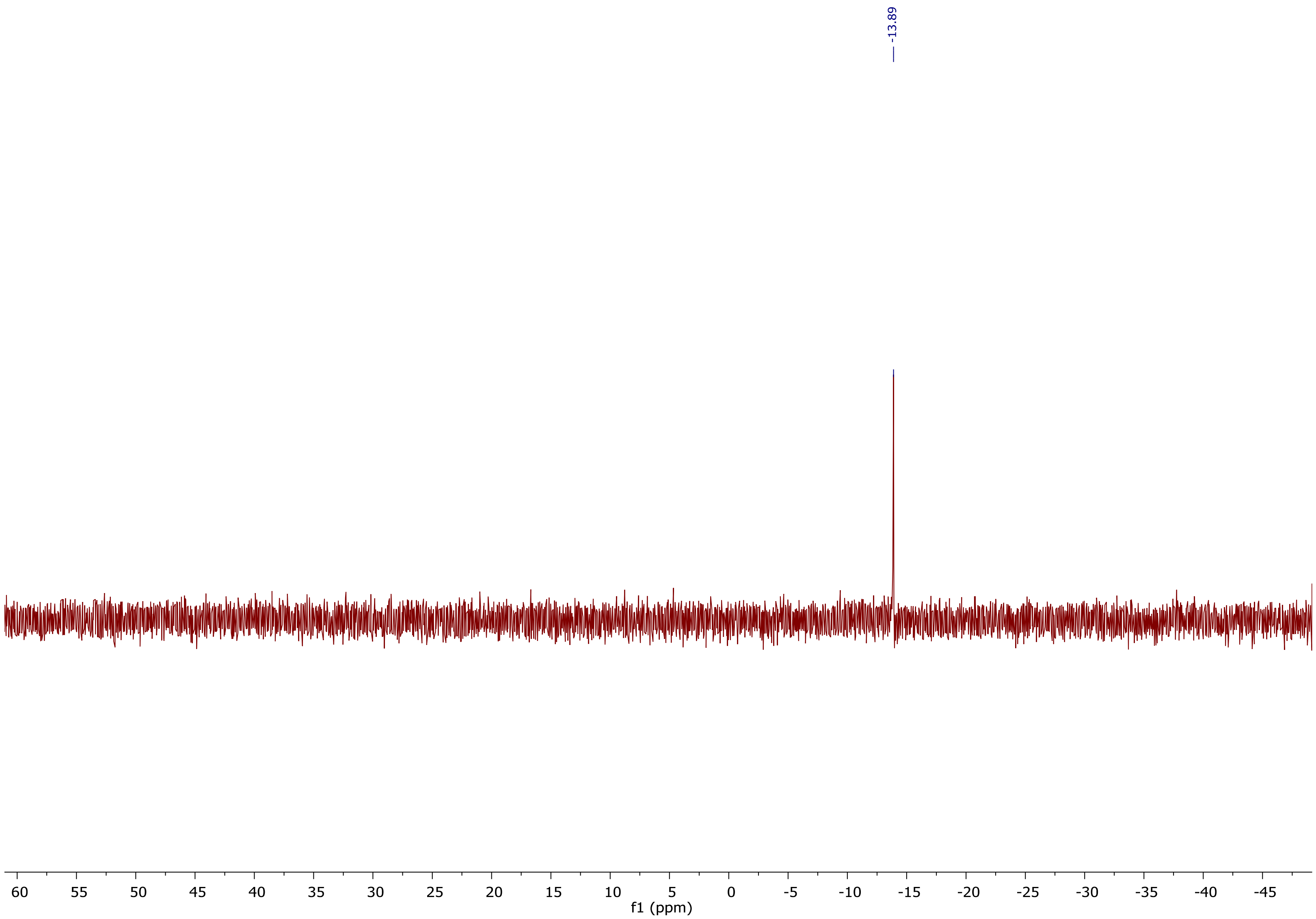

Thread 5 ^{13}C NMR ($CDCl_3$, 151 MHz, 300 K)

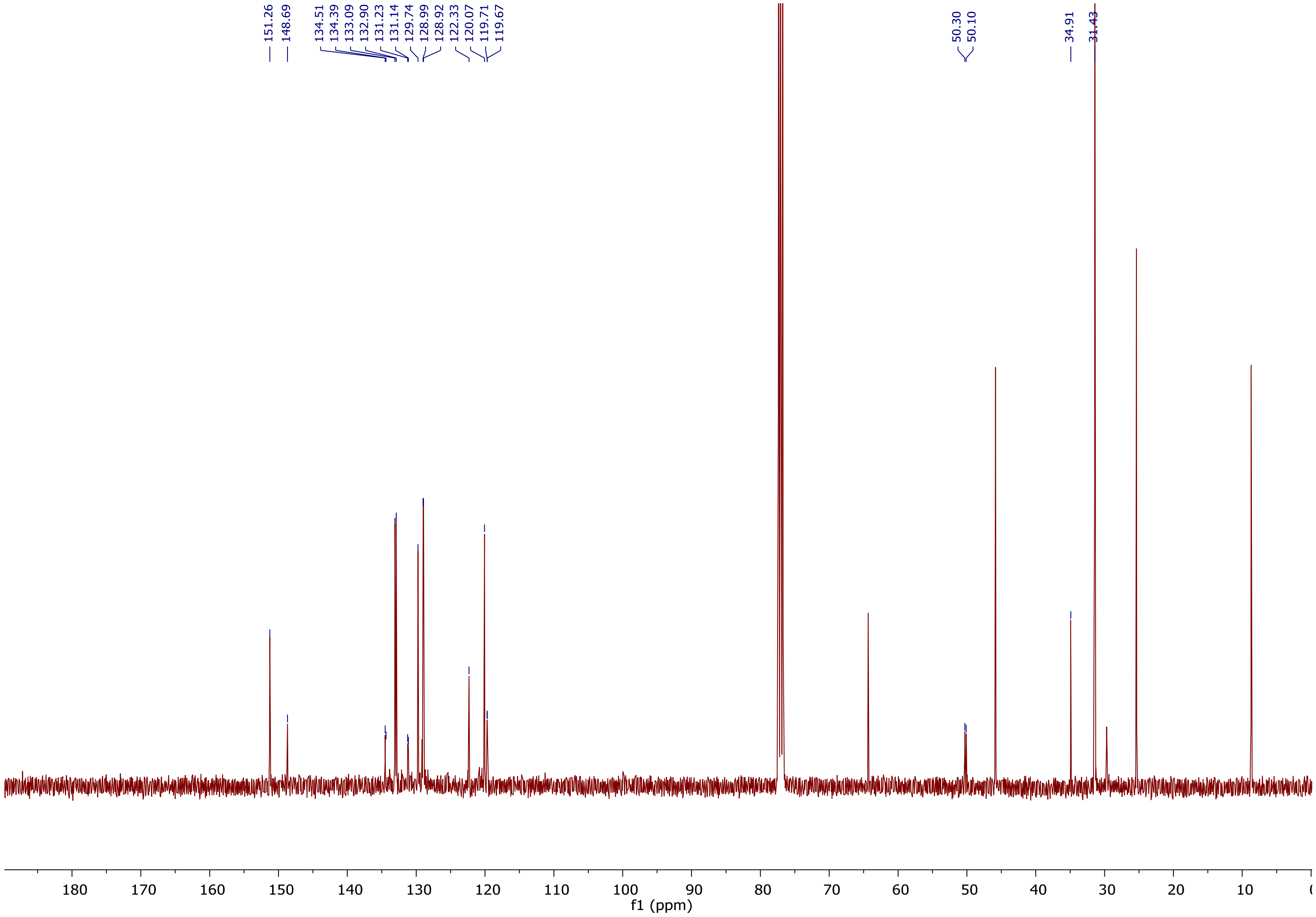

Thread 5 COSY NMR ($CDCl_3$, 600 MHz, 300 K)

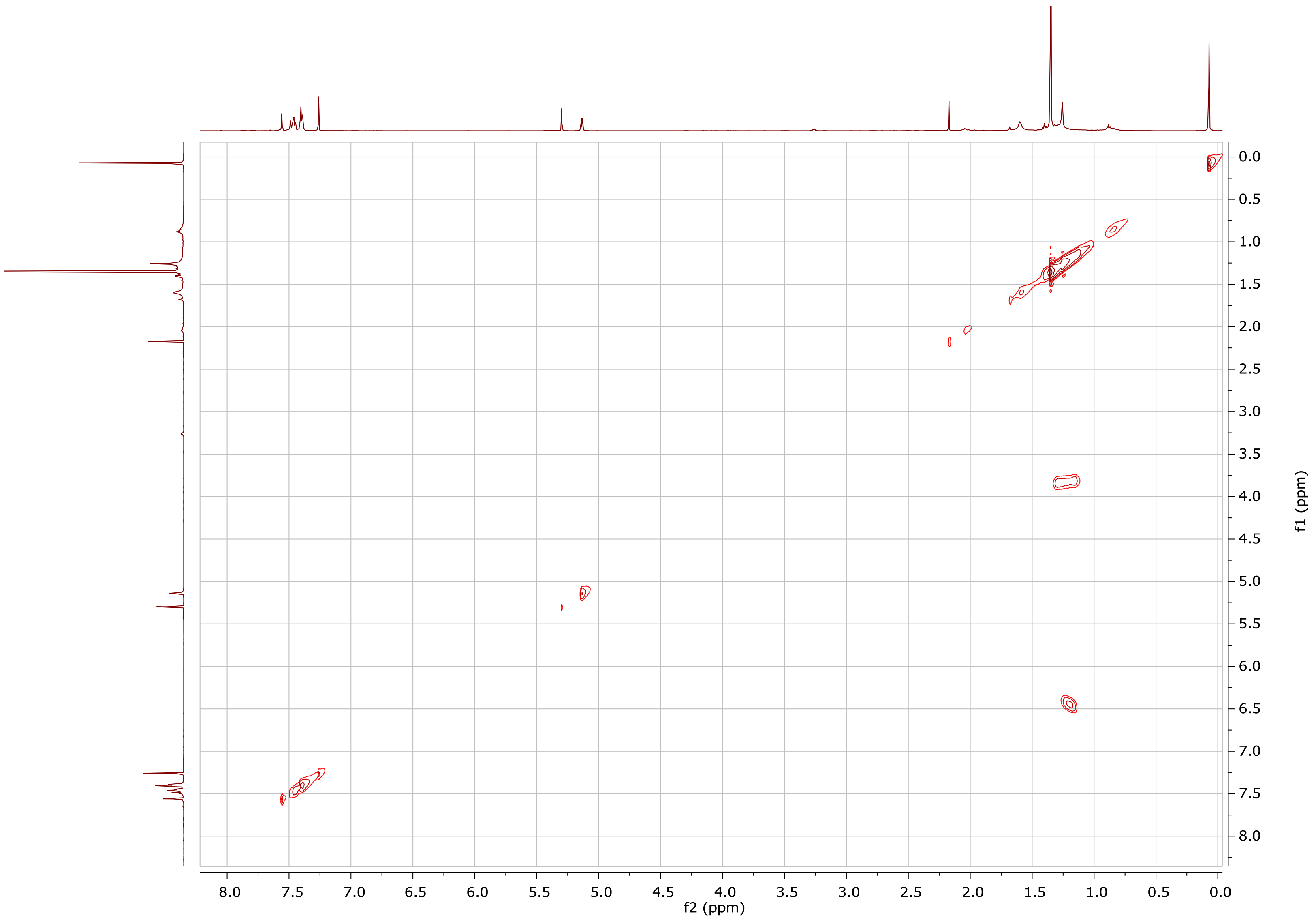

Thread [5AuCl] ^1H NMR ($CDCl_3$, 500 MHz, 300 K)

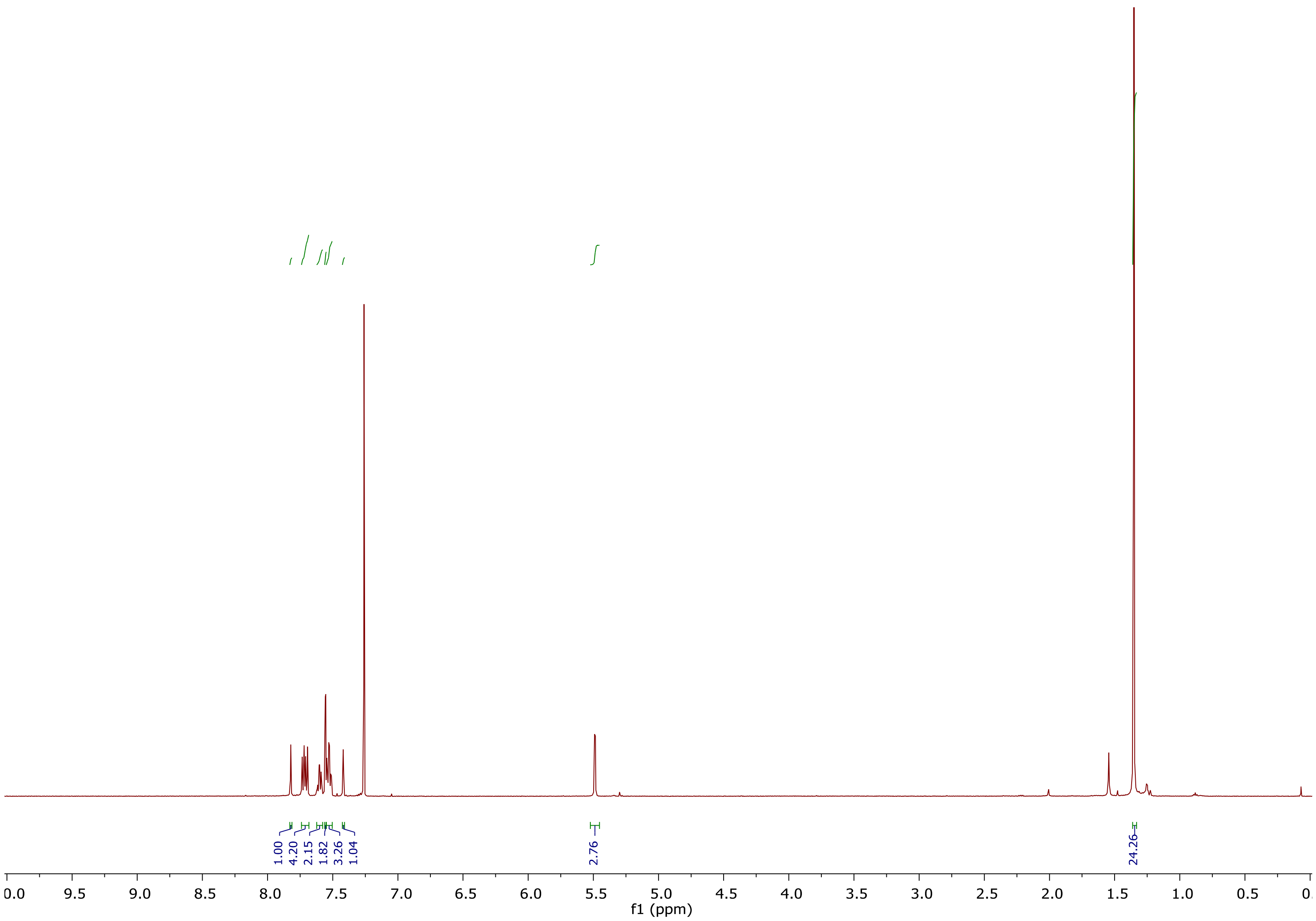

Thread [5AuCl] ^{31}P NMR ($CDCl_3$, 202 MHz, 300 K)

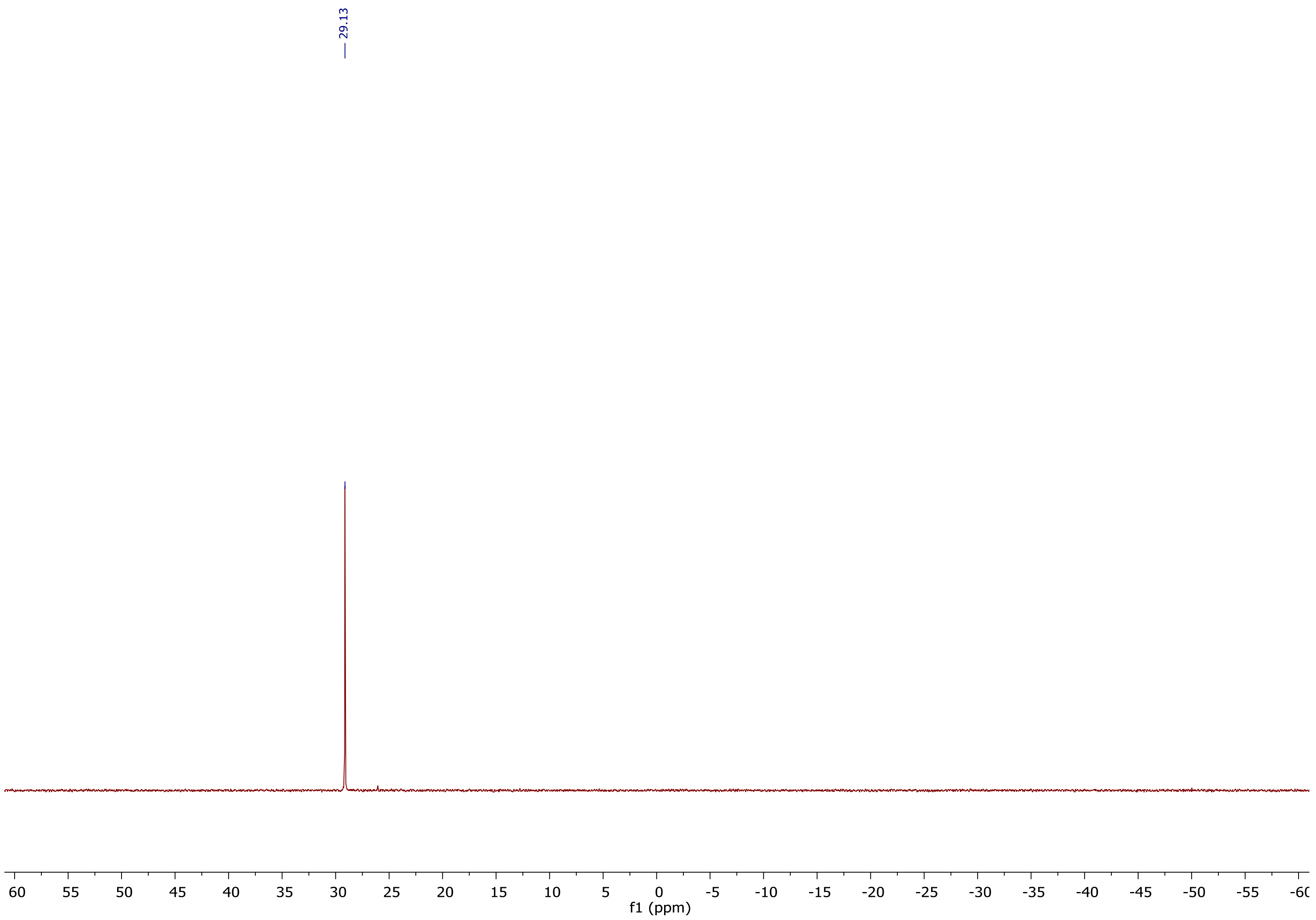

Thread [5AuCl] ^{13}C NMR ($CDCl_3$, 125 MHz, 300 K)

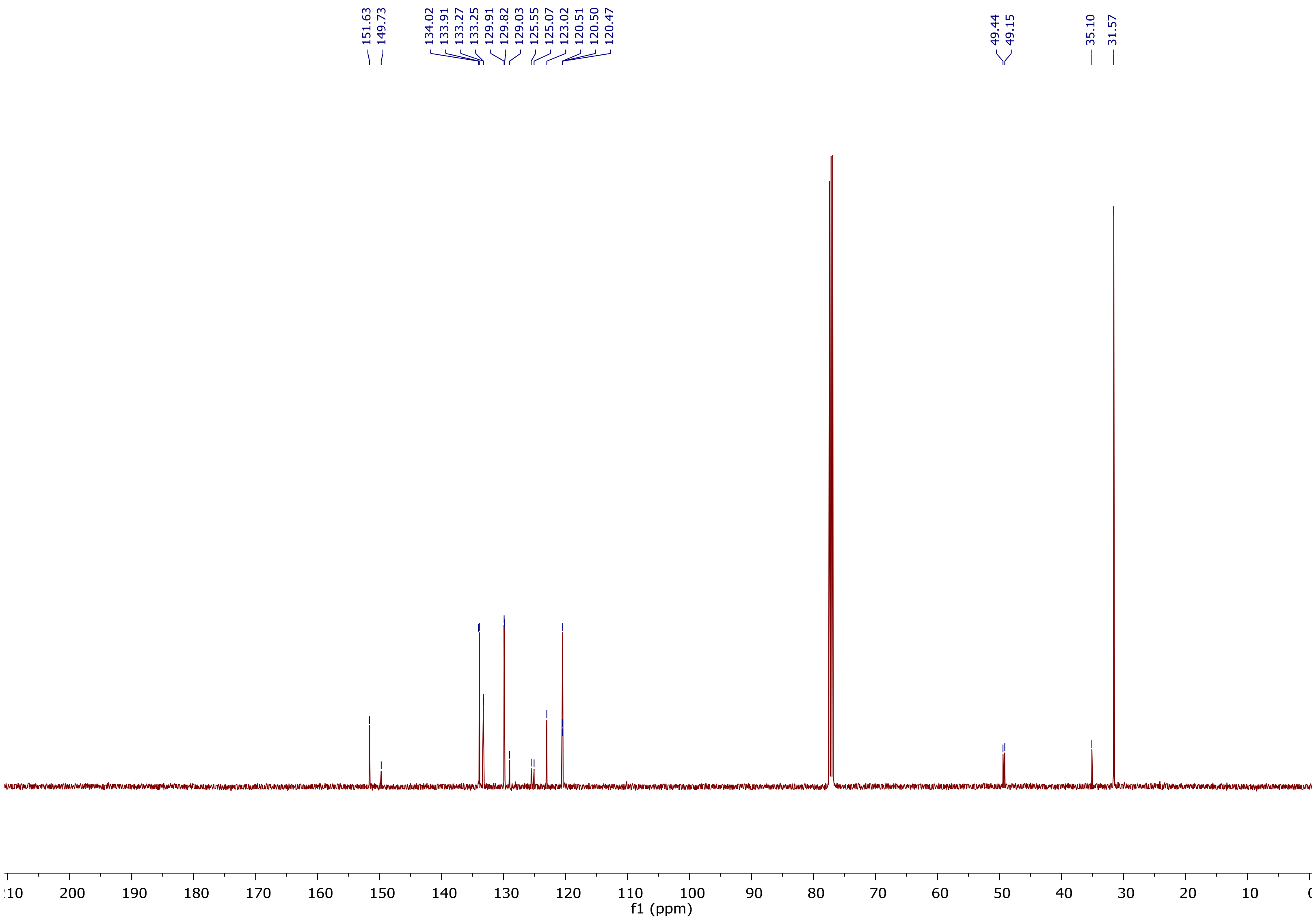

Thread [5AuCl] COSY ($CDCl_3$, 500 MHz, 300 K)

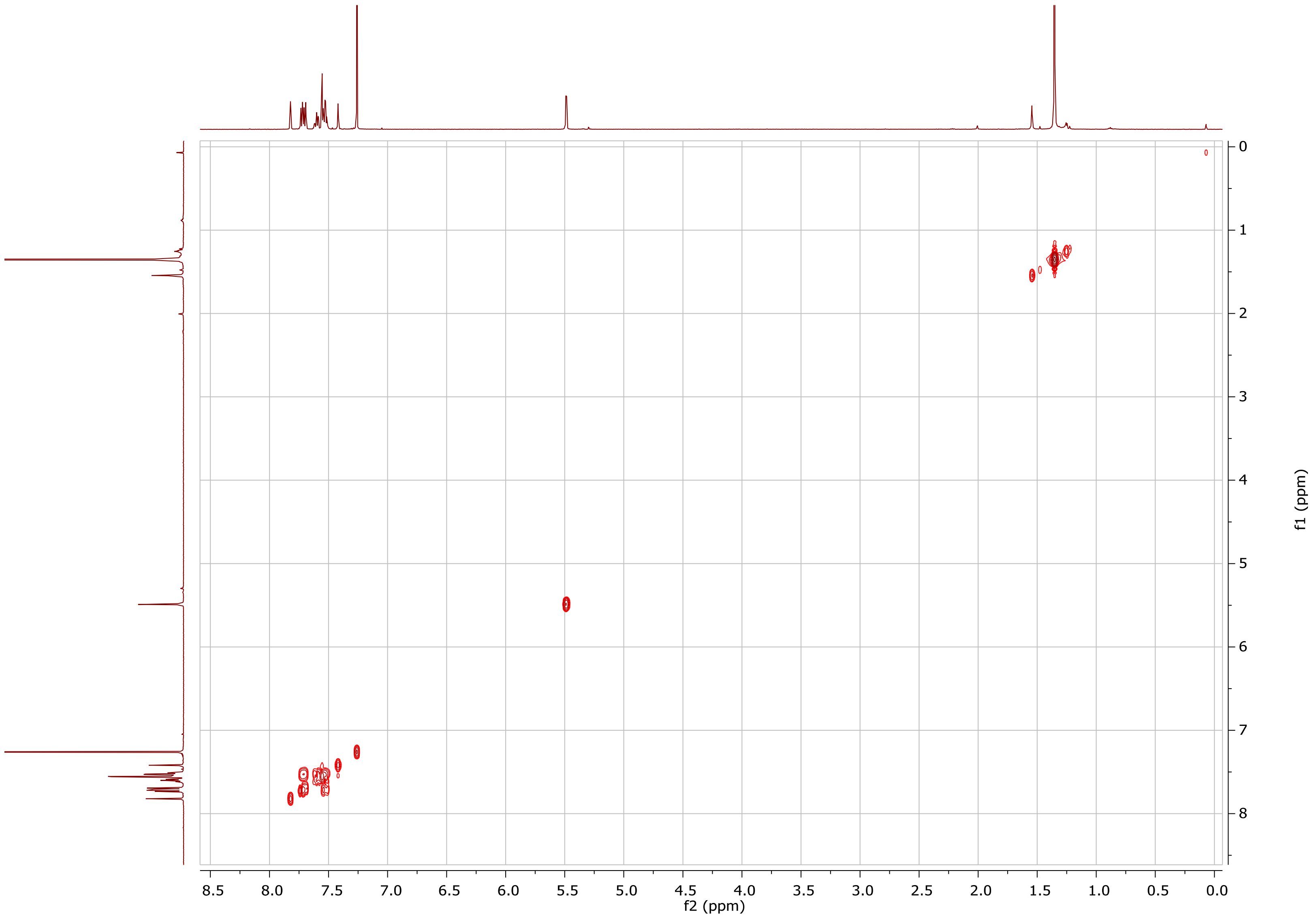

Thread [5AuCl] HSQC ($CDCl_3$, 500 MHz, 300 K)

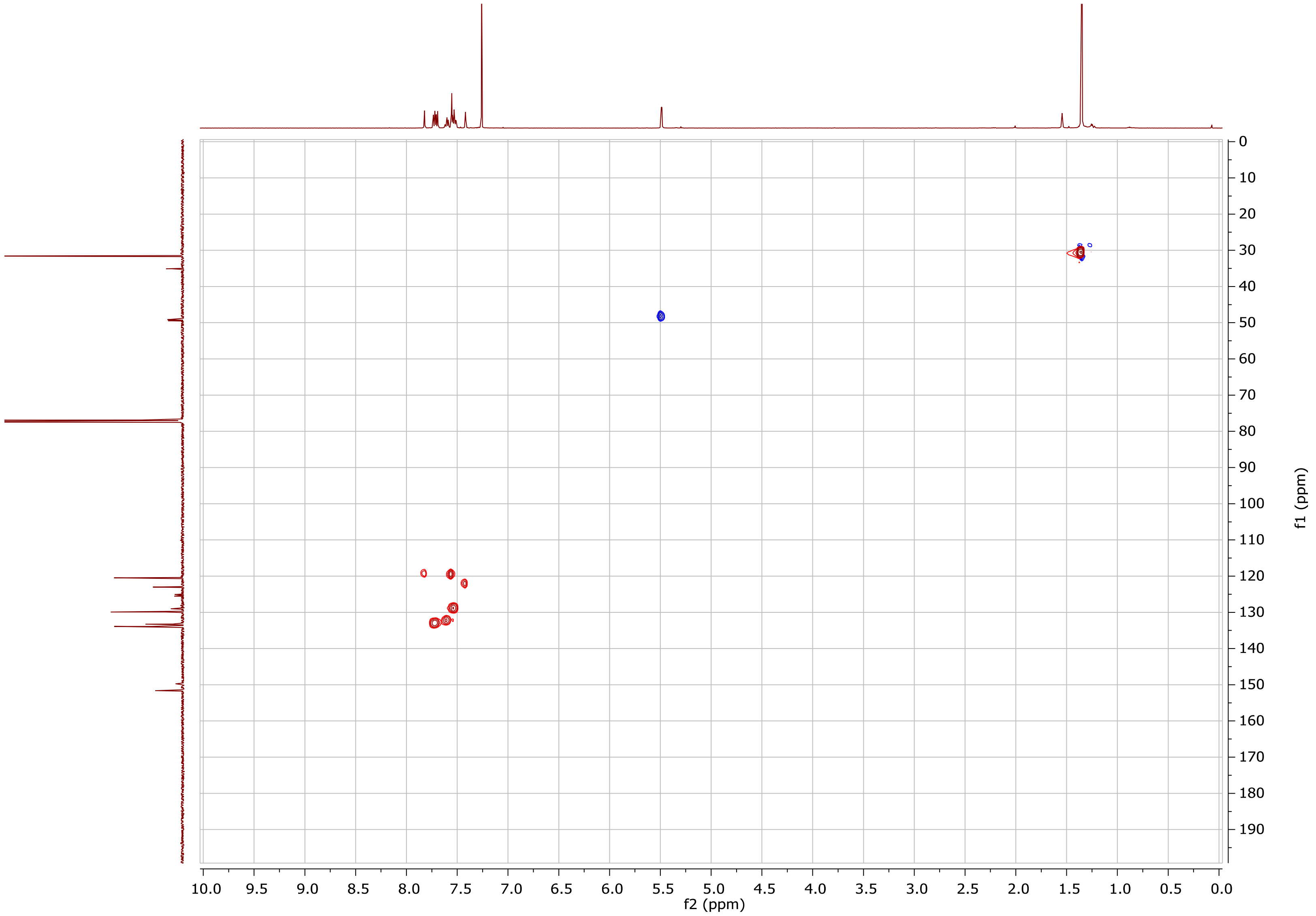

Thread [5AuCl] HMBC ($CDCl_3$, 500 MHz, 300 K)

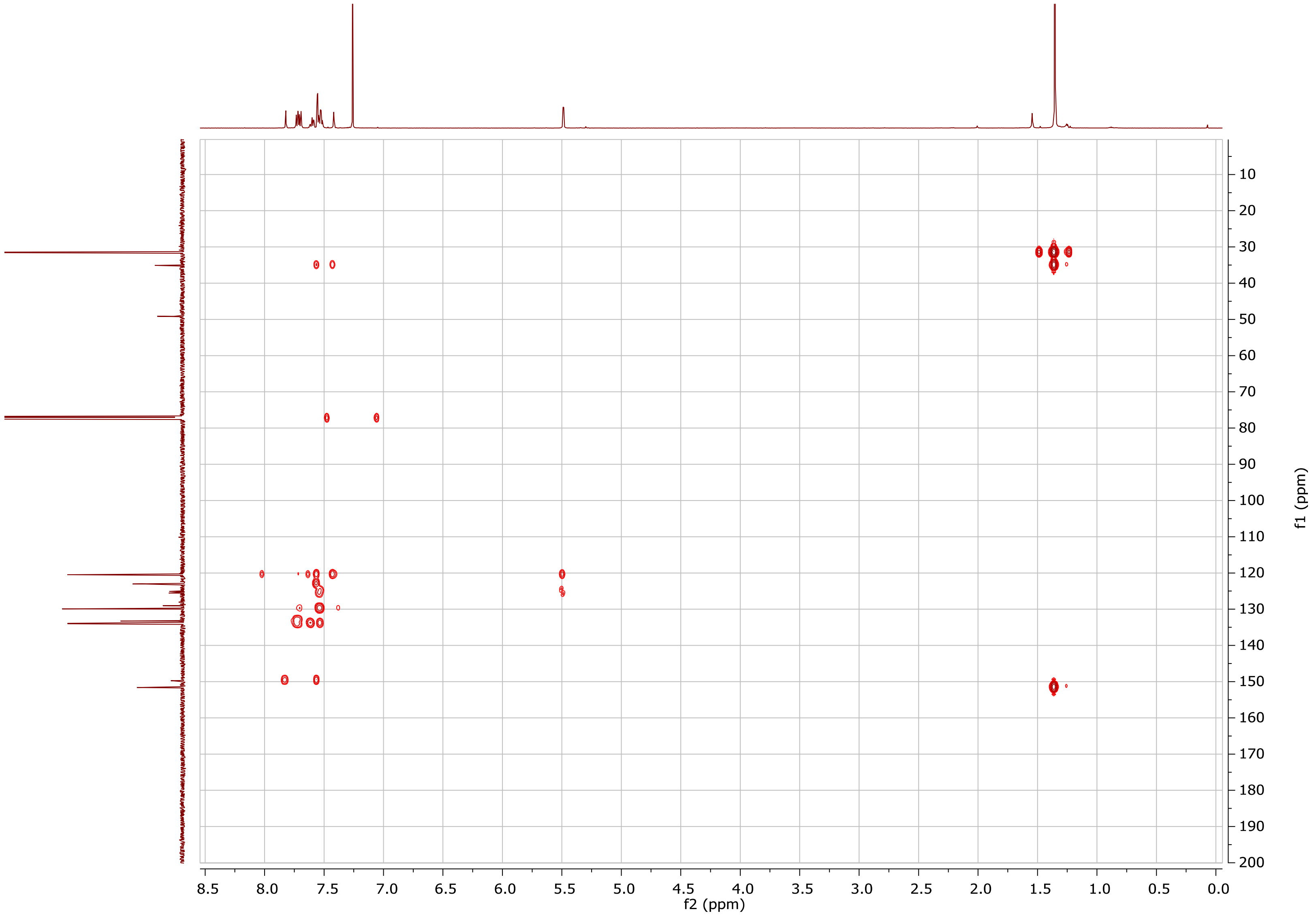

Rotaxane [4AuClH]$AuCl_2$ ^{1}H NMR ($CDCl_3$, 400 MHz, 300 K)

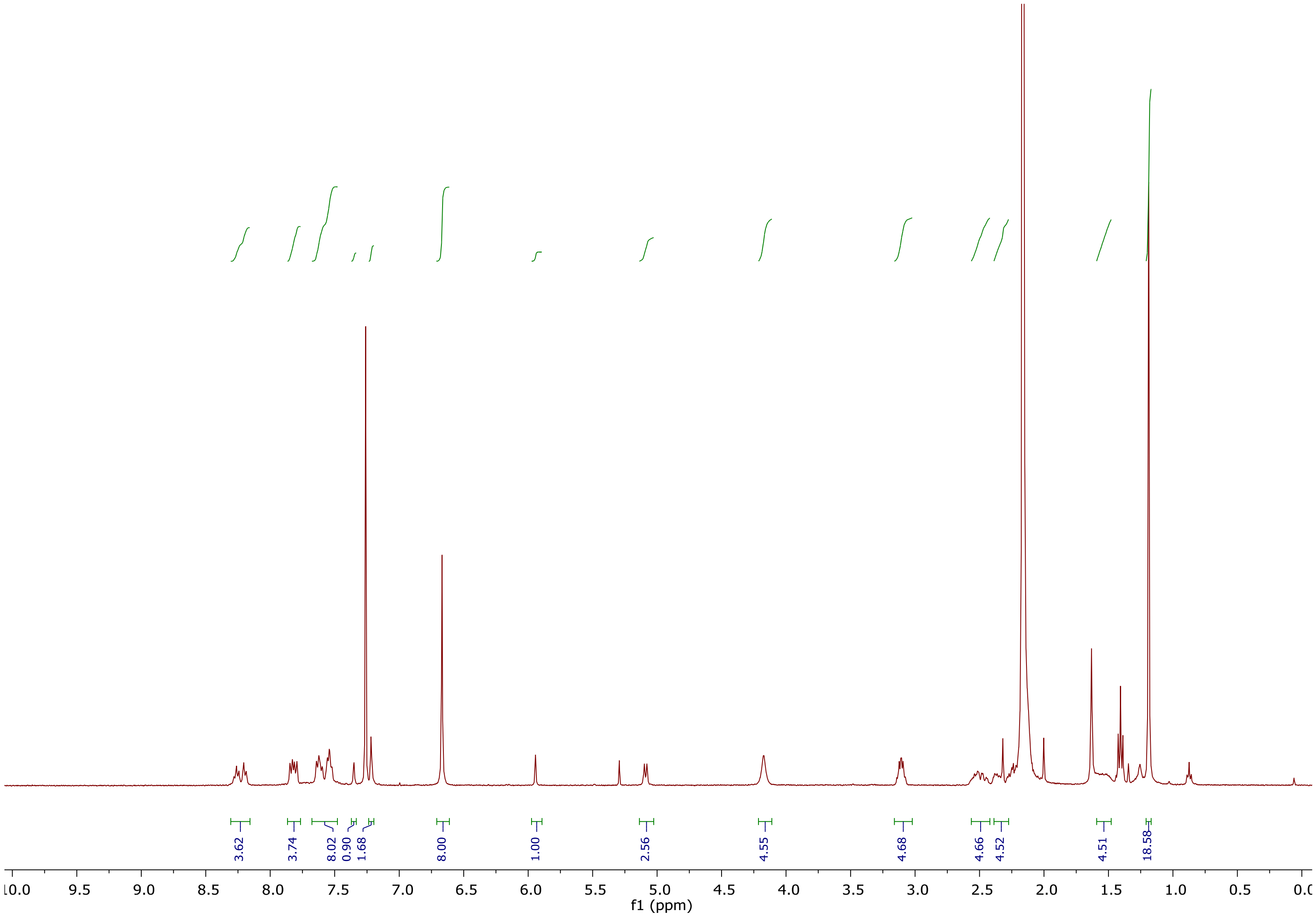

Rotaxane [4AuClH]$AuCl_2$ ^{31}P NMR ($CDCl_3$, 160 MHz, 300 K)

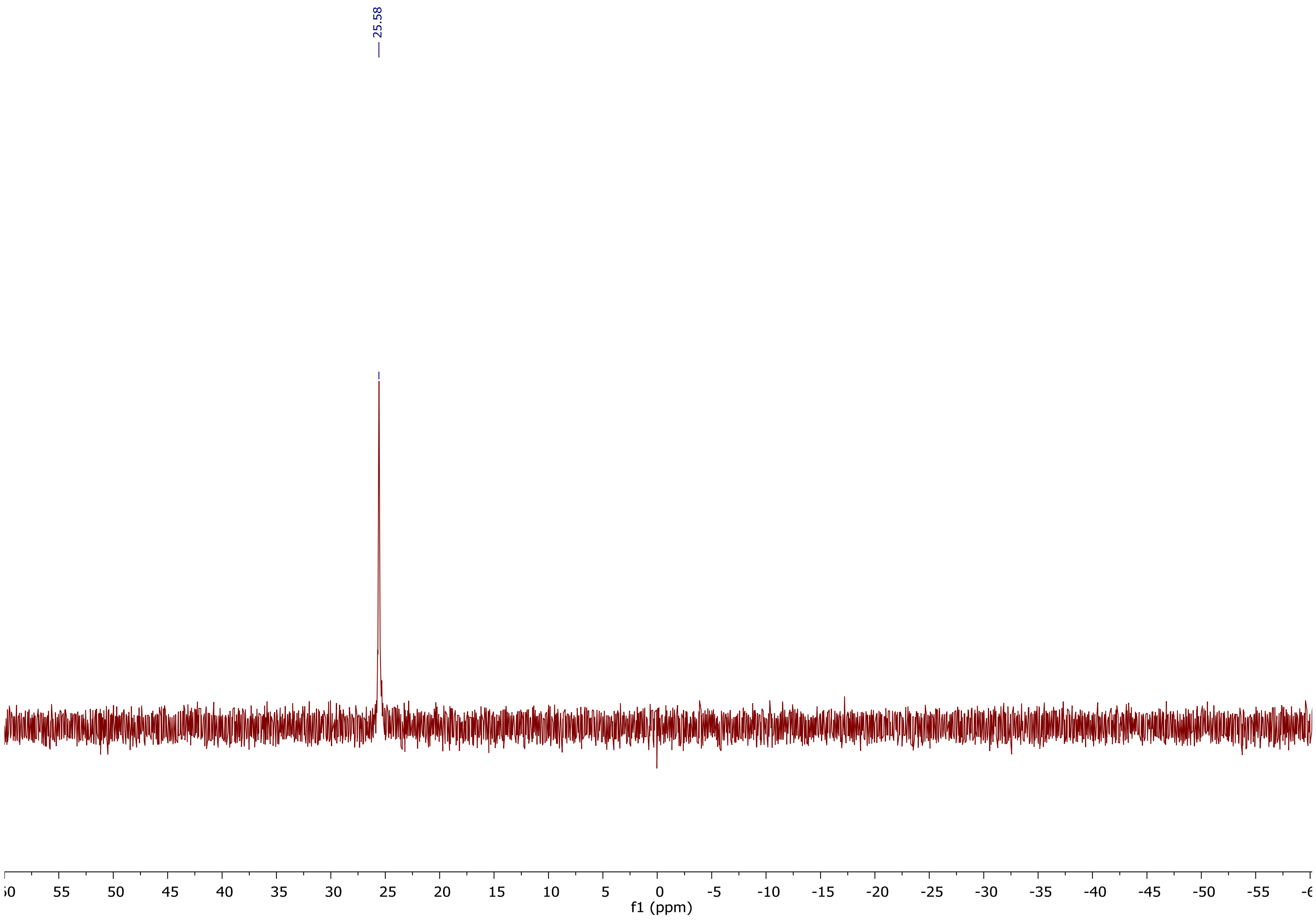

Rotaxane [4Au]SbF_6 ^1H NMR ($CDCl_3$, 600 MHz, 300 K)

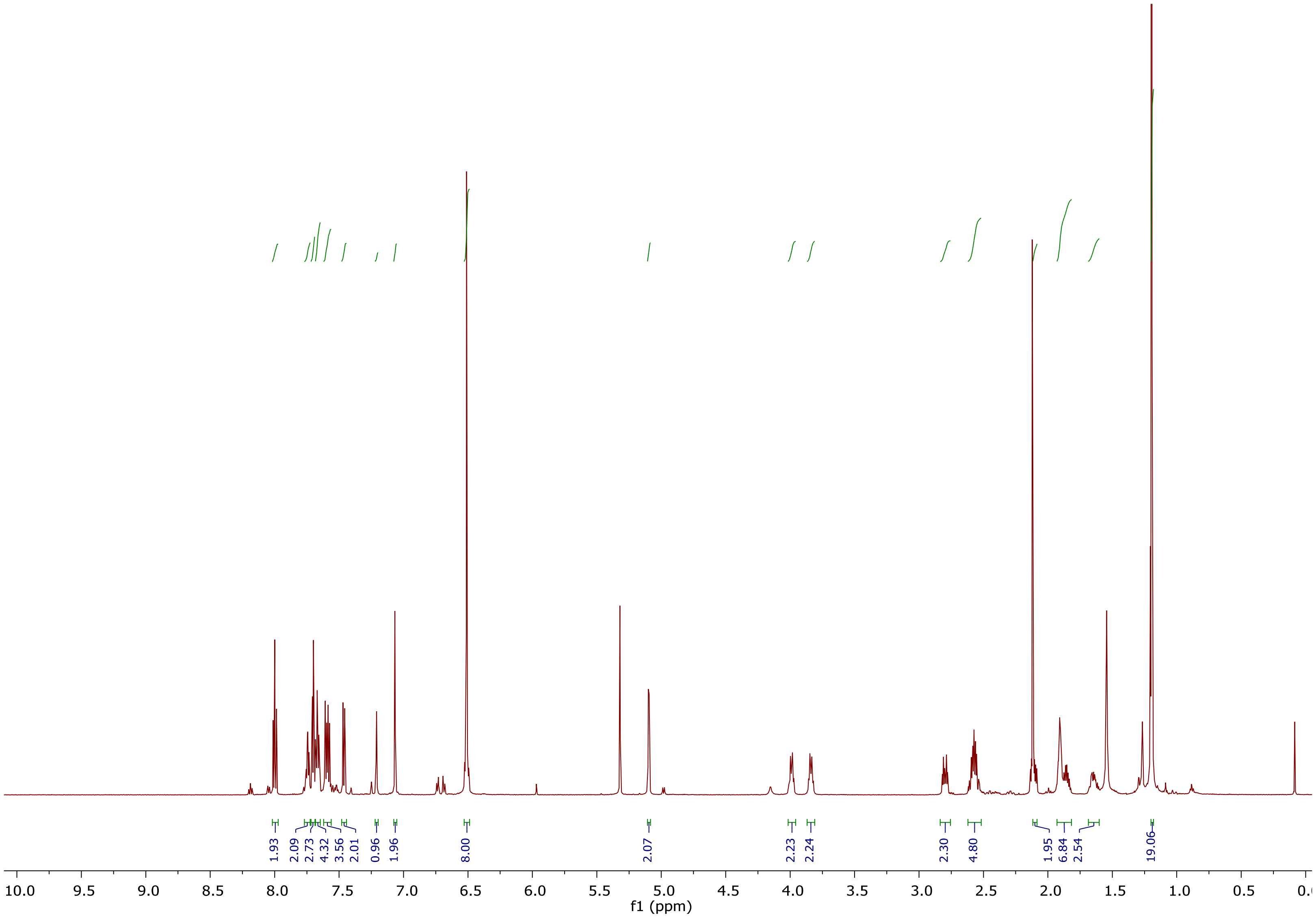

Rotaxane [4Au]SbF_6 ^{31}P NMR ($CDCl_3$, 240 MHz, 300 K)

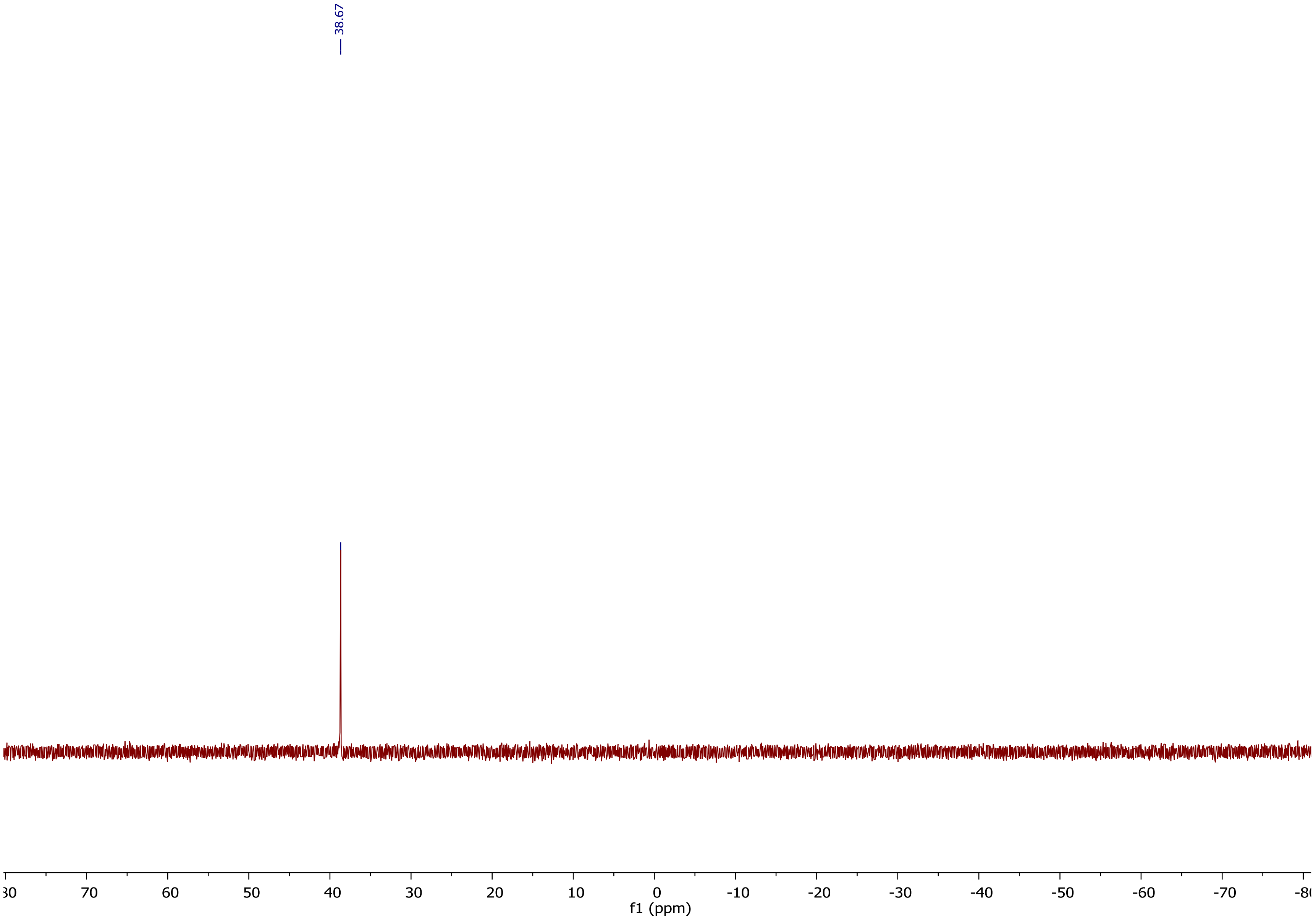

Rotaxane [4Au]SbF_6 ^{13}C NMR ($CDCl_3$, 151 MHz, 300 K)

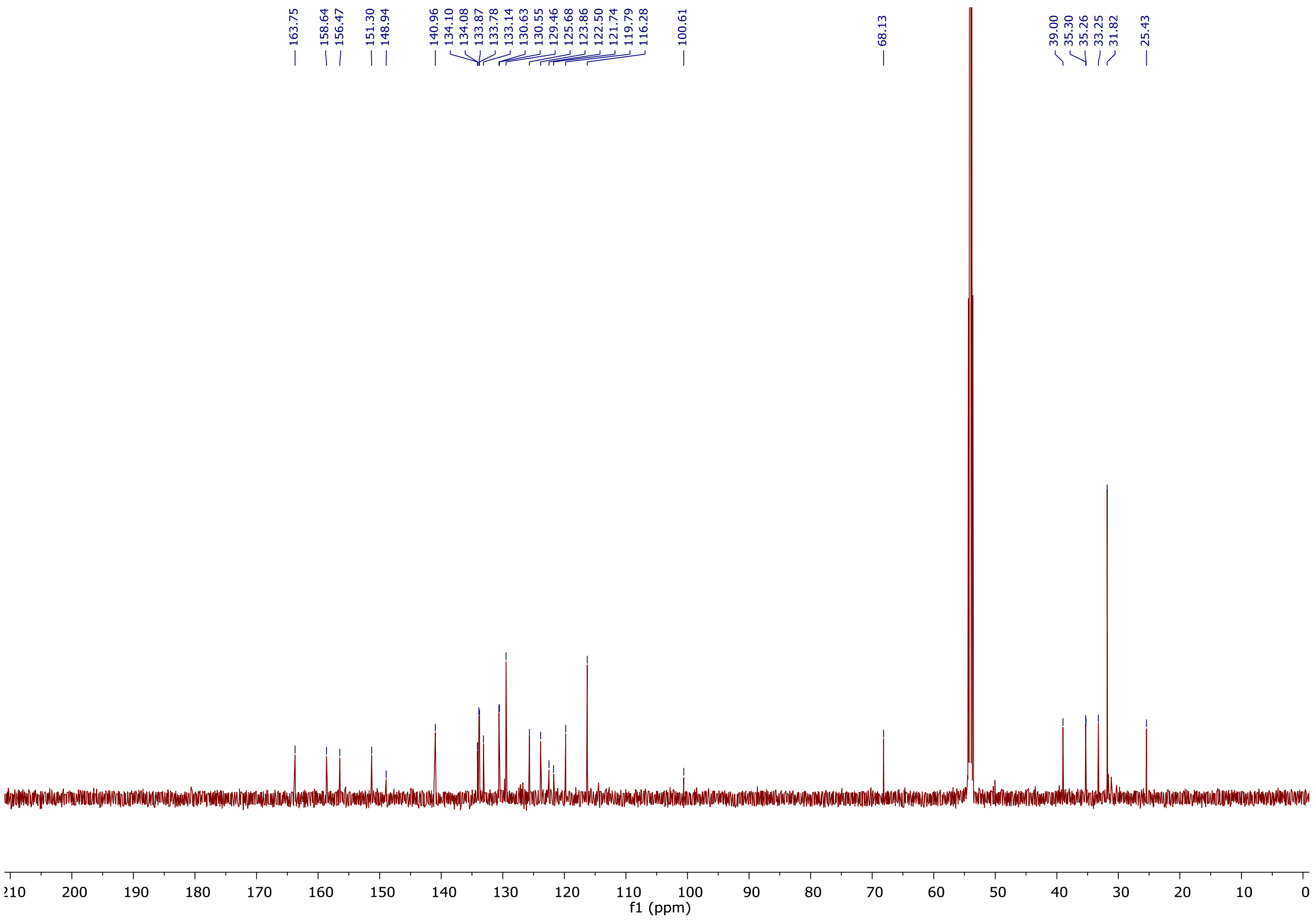

Rotaxane [4Au]SbF_6 COSY ($CDCl_3$, 600 MHz, 300 K)

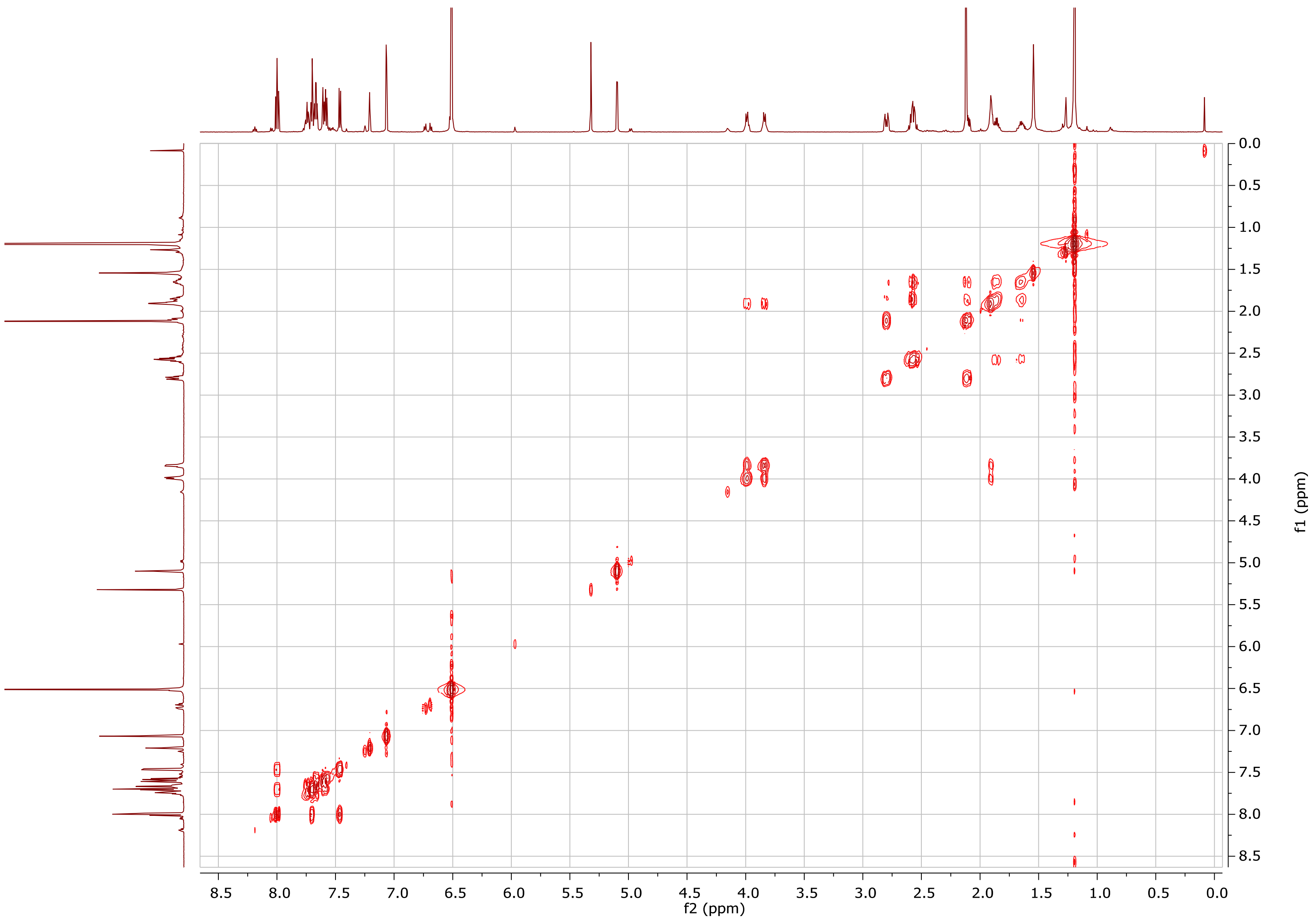

Rotaxane [4Au]SbF$_6$ HSQC ($CDCl_3$, 600 MHz, 300 K)

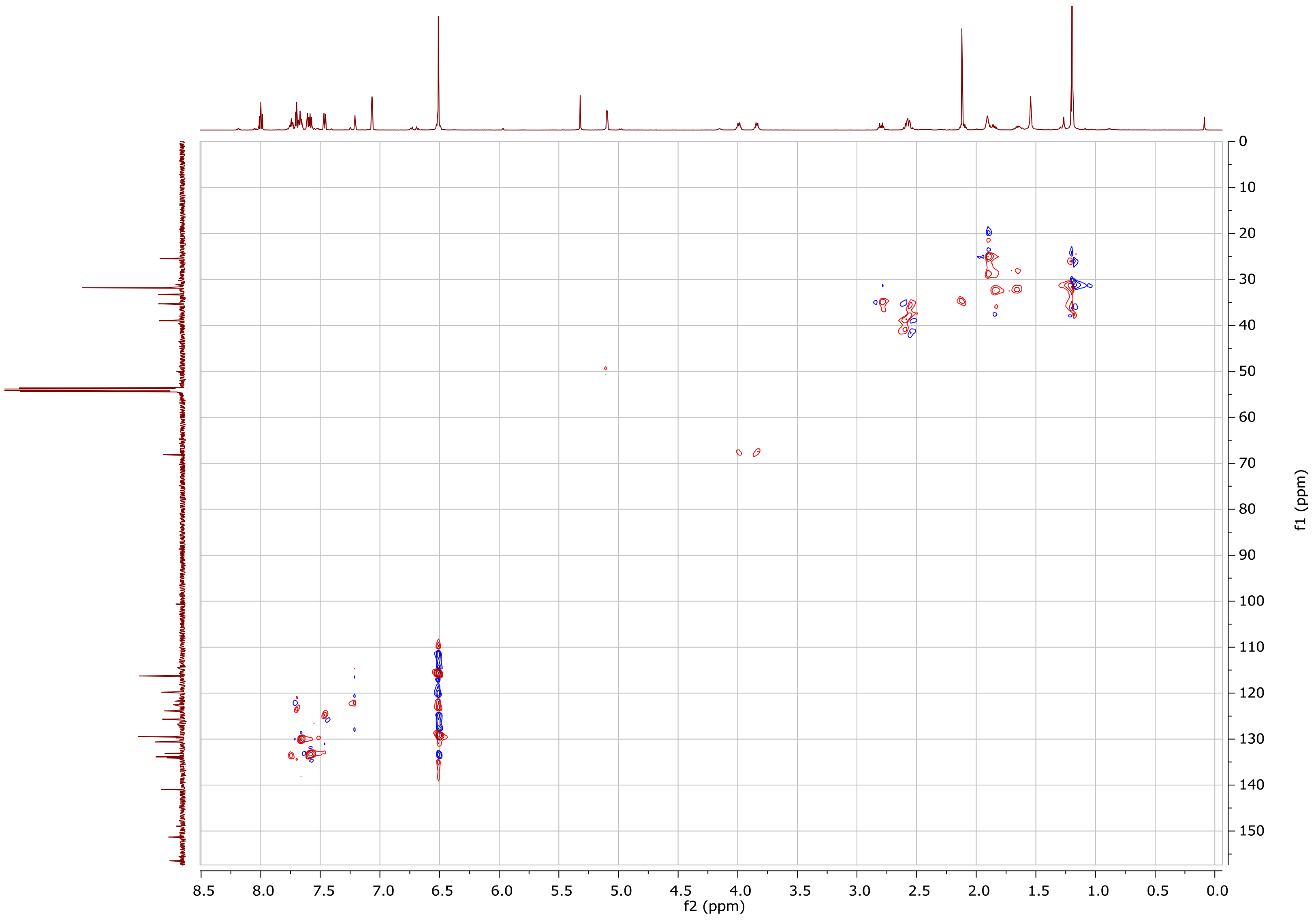

Rotaxane [4Au]SbF_6 HMBC ($CDCl_3$, 600 MHz, 300 K)

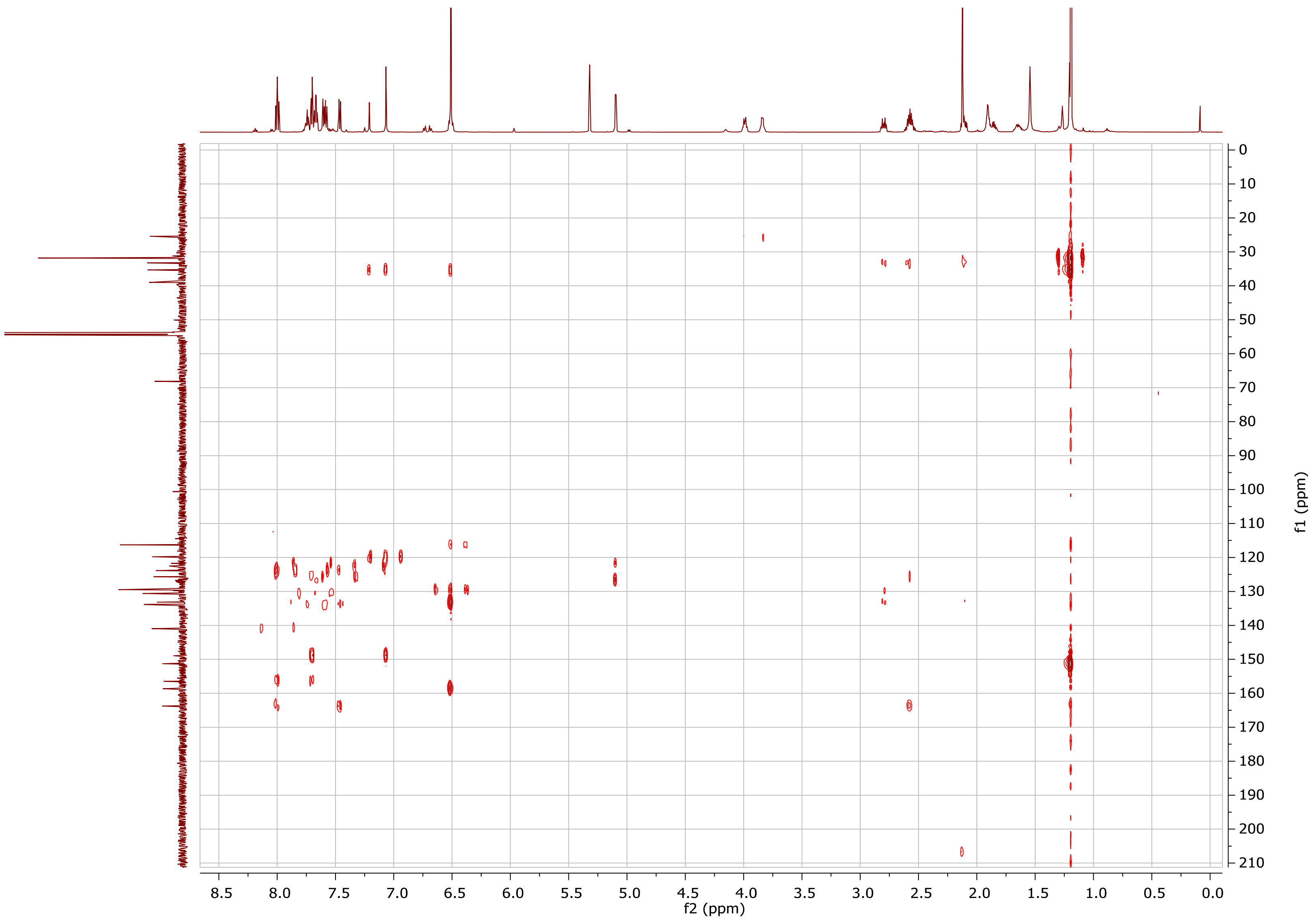

Thread [5Au]SbF_6 ^{1}H NMR (CD_2Cl_2, 500 MHz, 300 K)

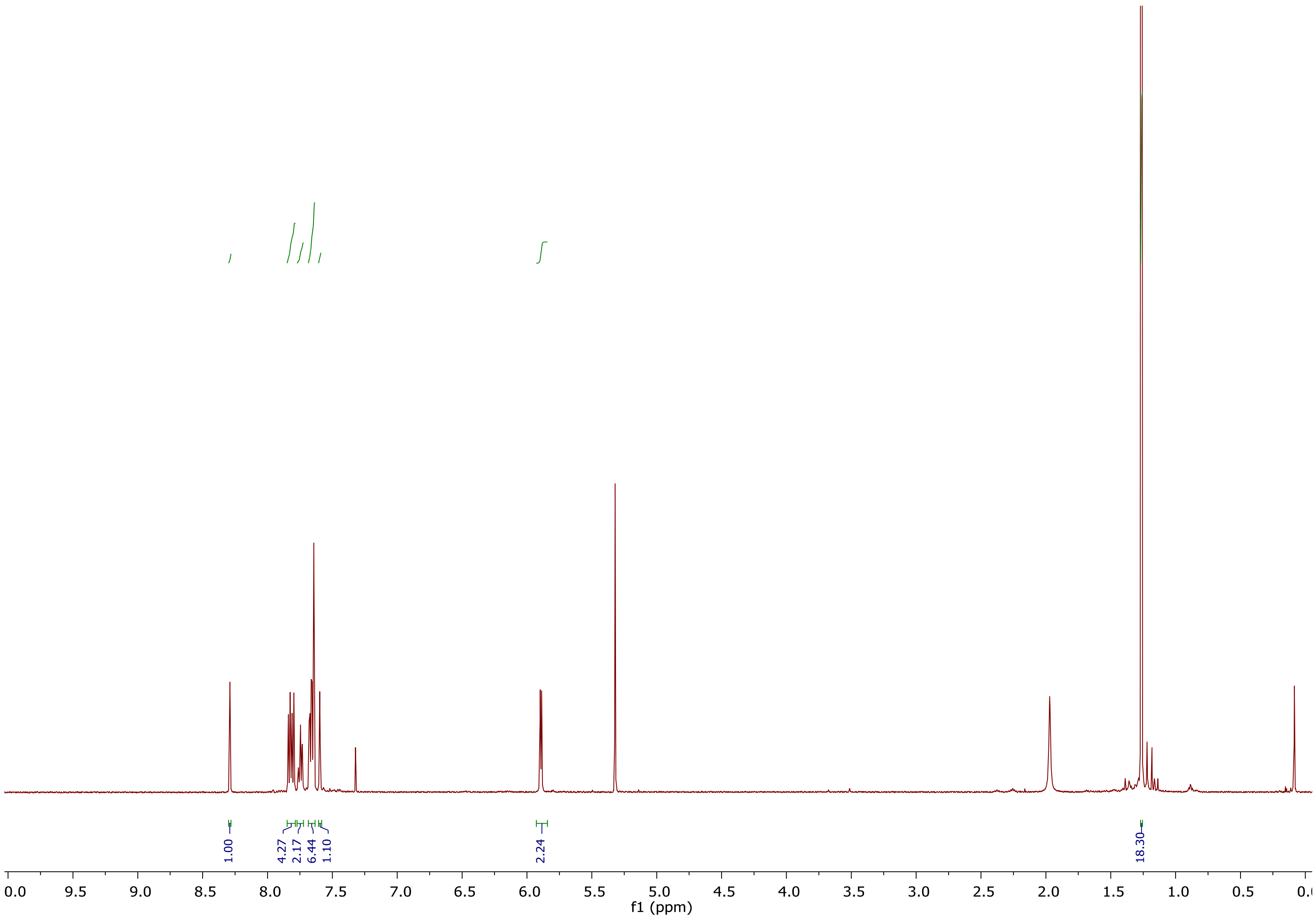

Thread [5Au]SbF_6 ^{31}P NMR (CD_2Cl_2, 500 MHz, 300 K)

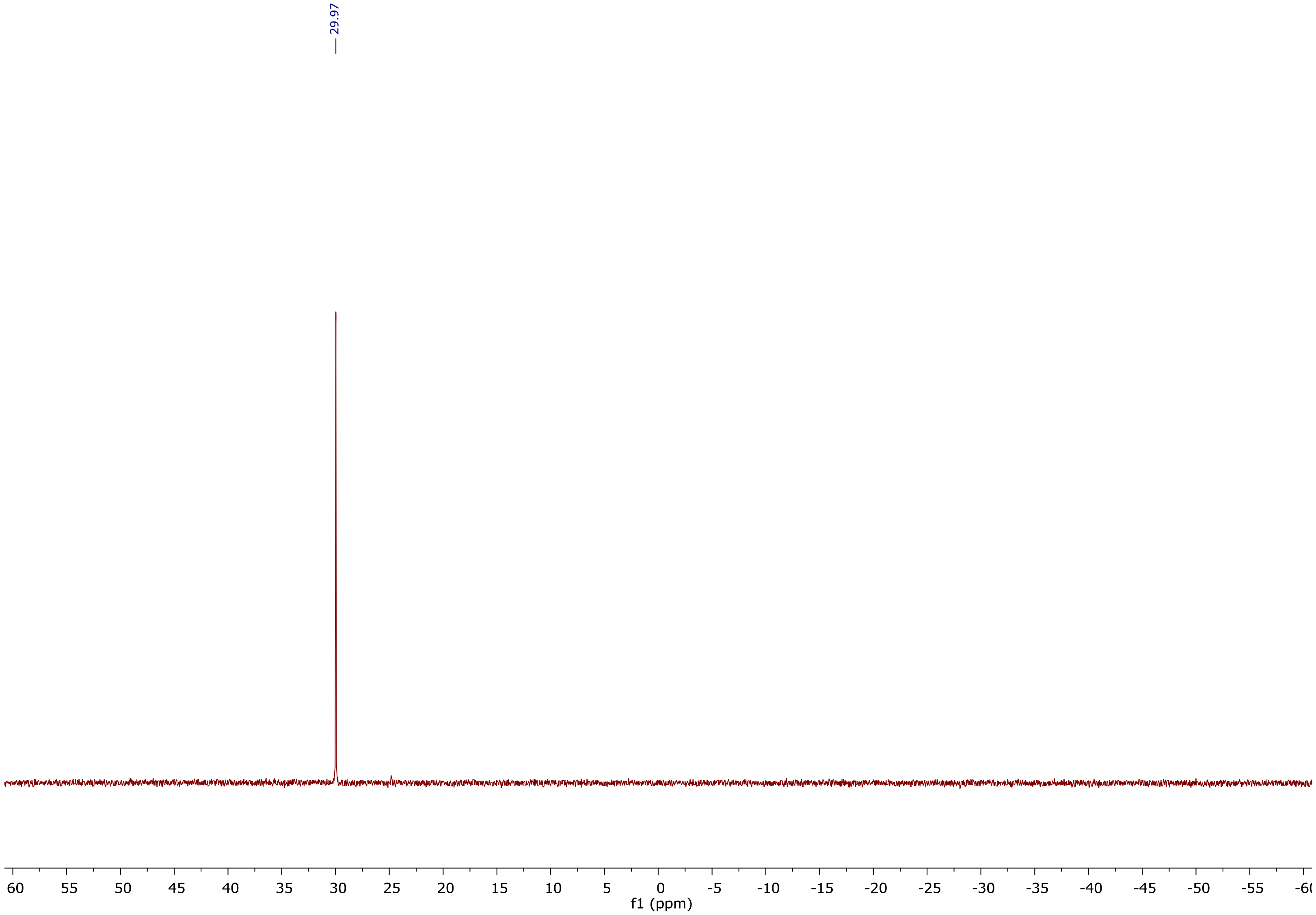

Thread [5Au]SbF_6 ^{13}C NMR (CD_2Cl_2, 125 MHz, 300 K)

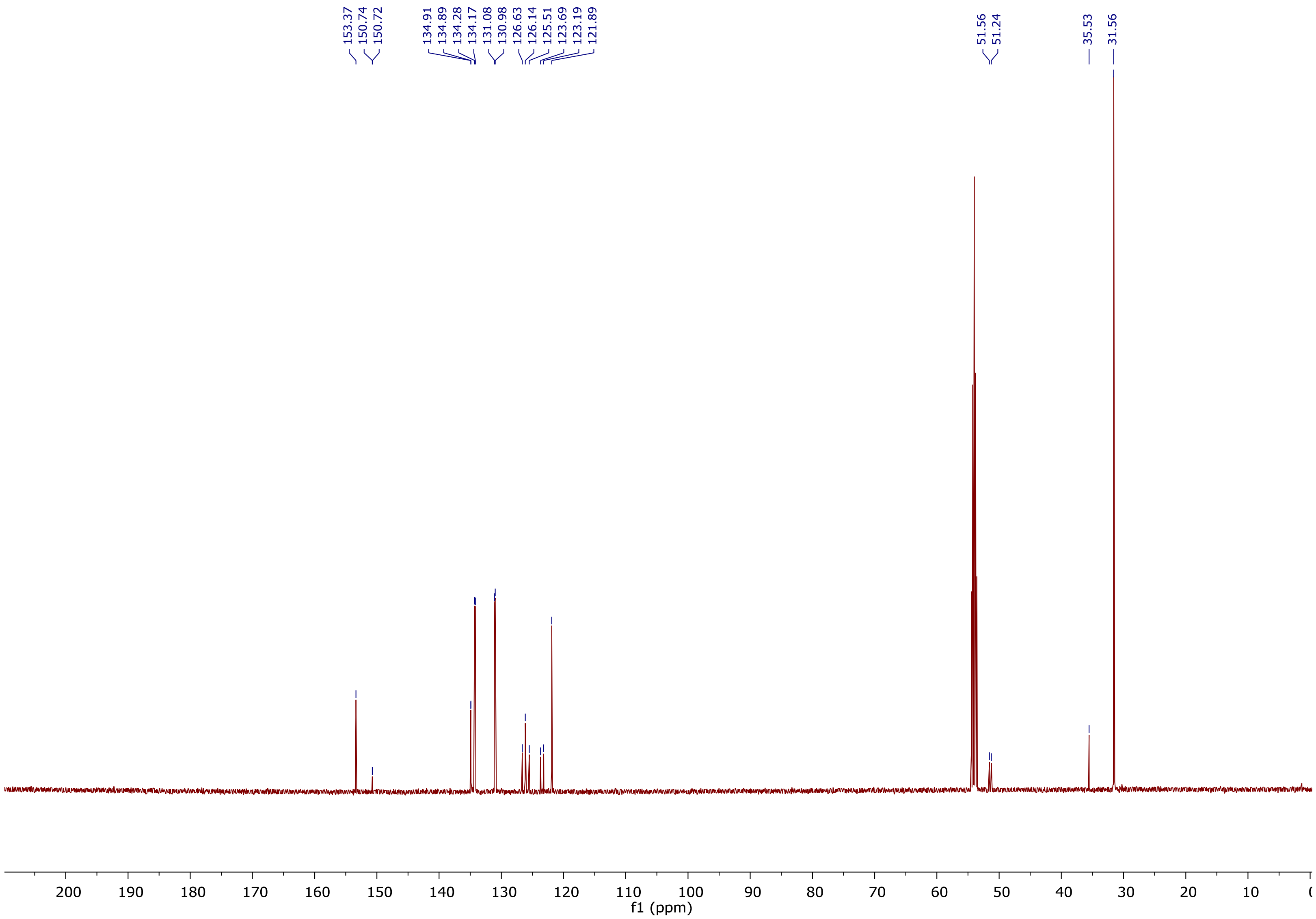

Thread [5Au]SbF_6 COSY (CD_2Cl_2, 500 MHz, 300 K)

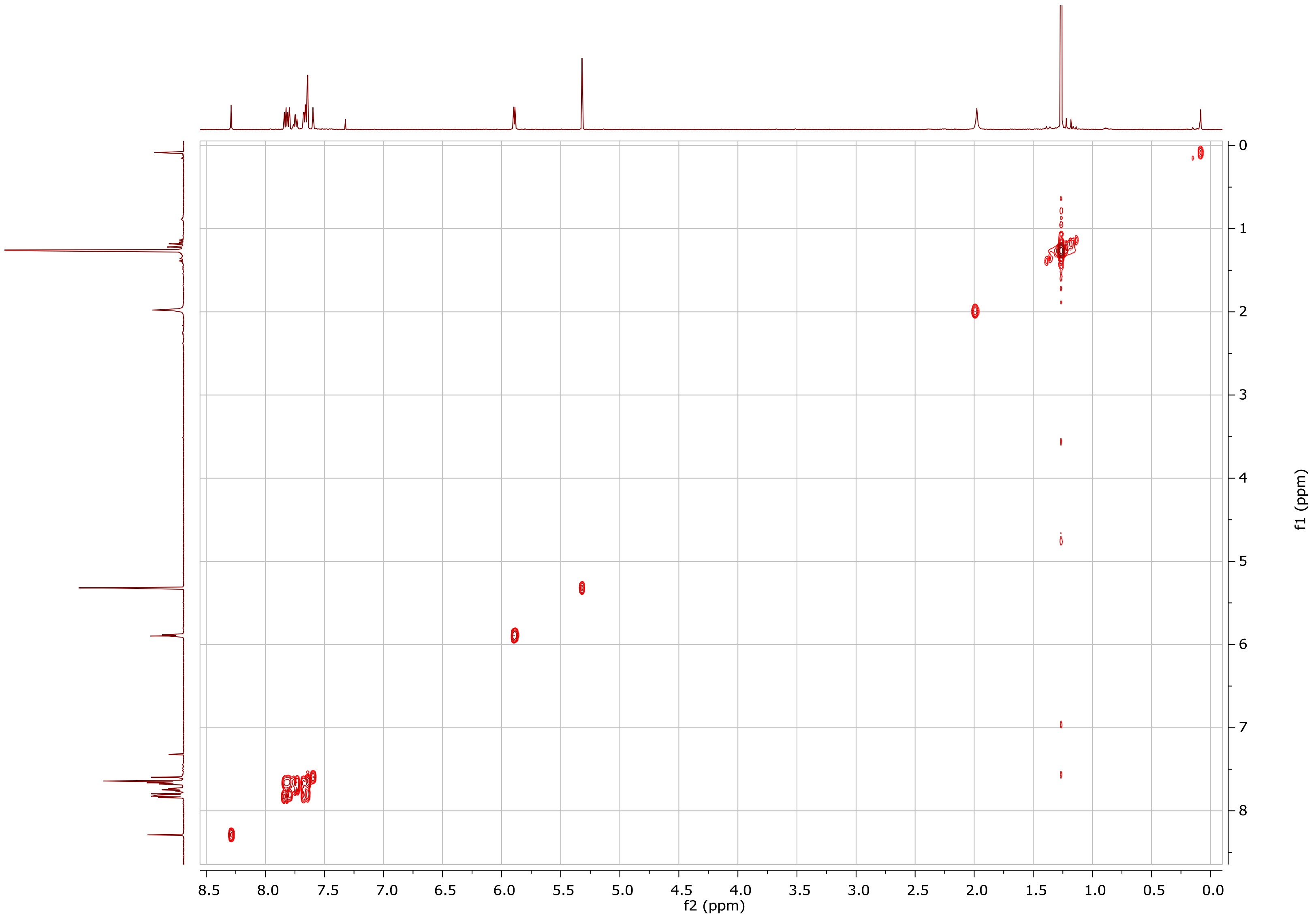

Thread [5Au]SbF_6 HSQC (CD_2Cl_2, 500 MHz, 300 K)

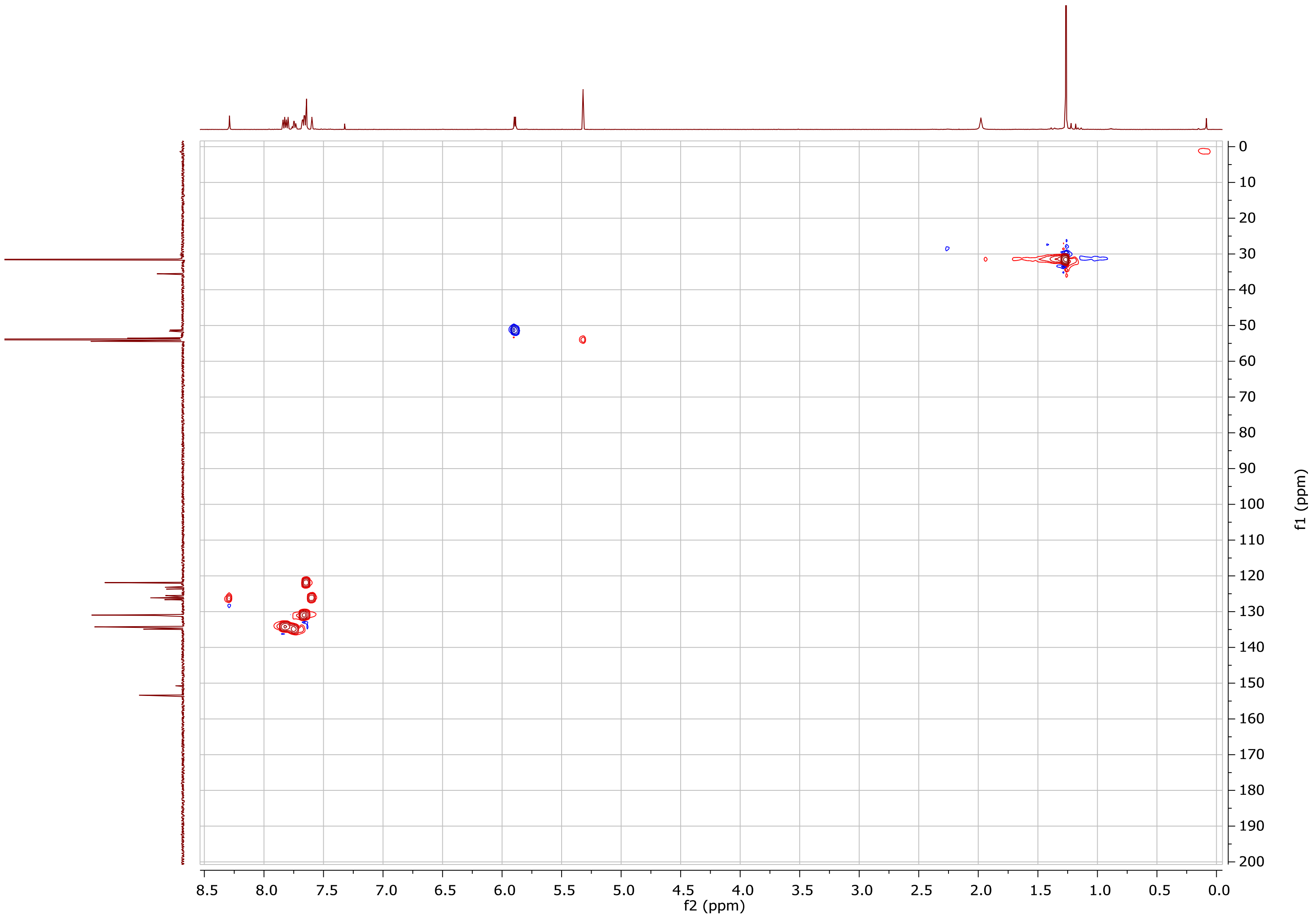

Thread [5Au]SbF_6 HMBC (CD_2Cl_2, 500 MHz, 300 K)

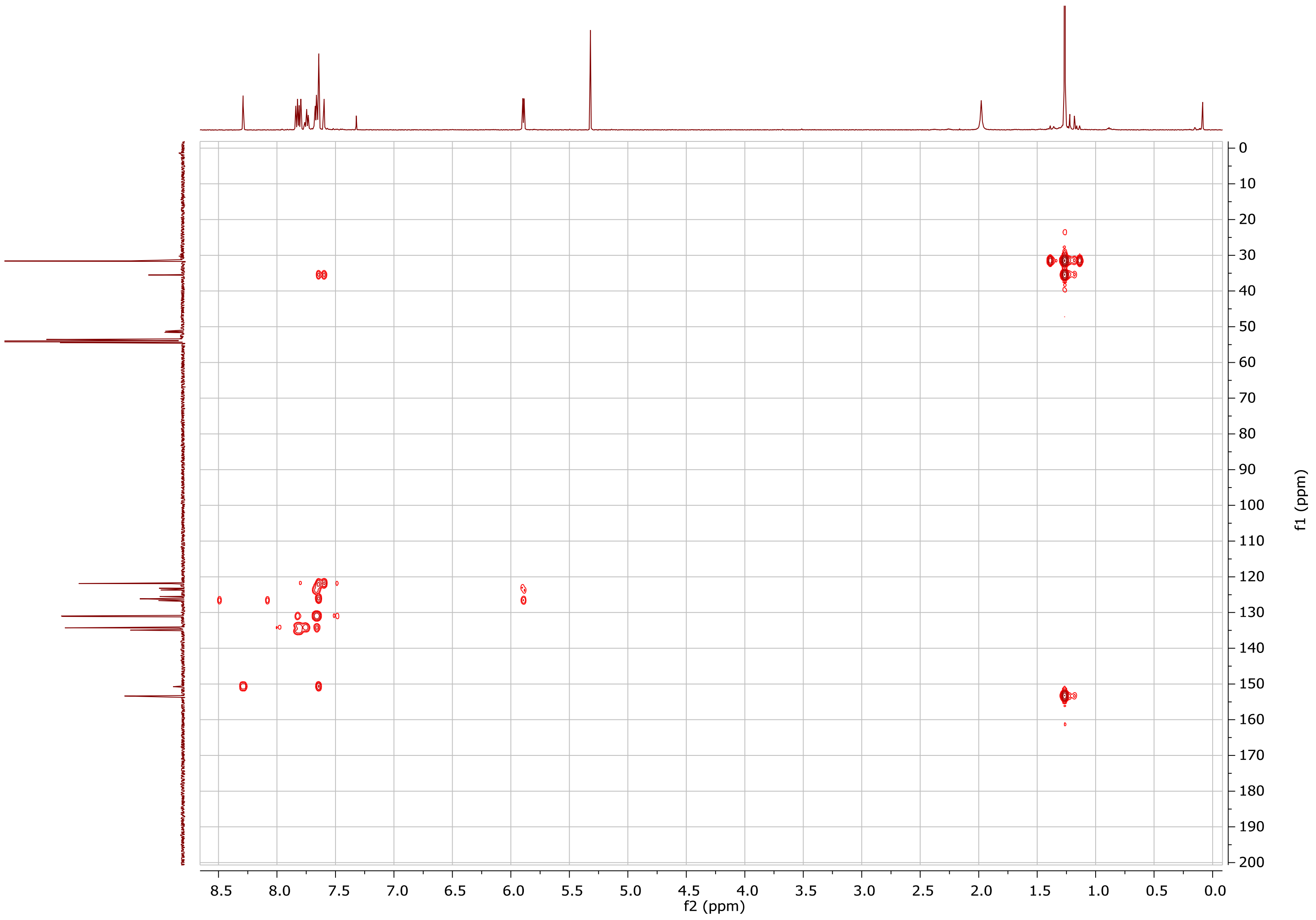

Rotaxane [4AuCu]PF_6SbF_6 ^1H NMR ($CDCl_3$, 400 MHz, 300 K)

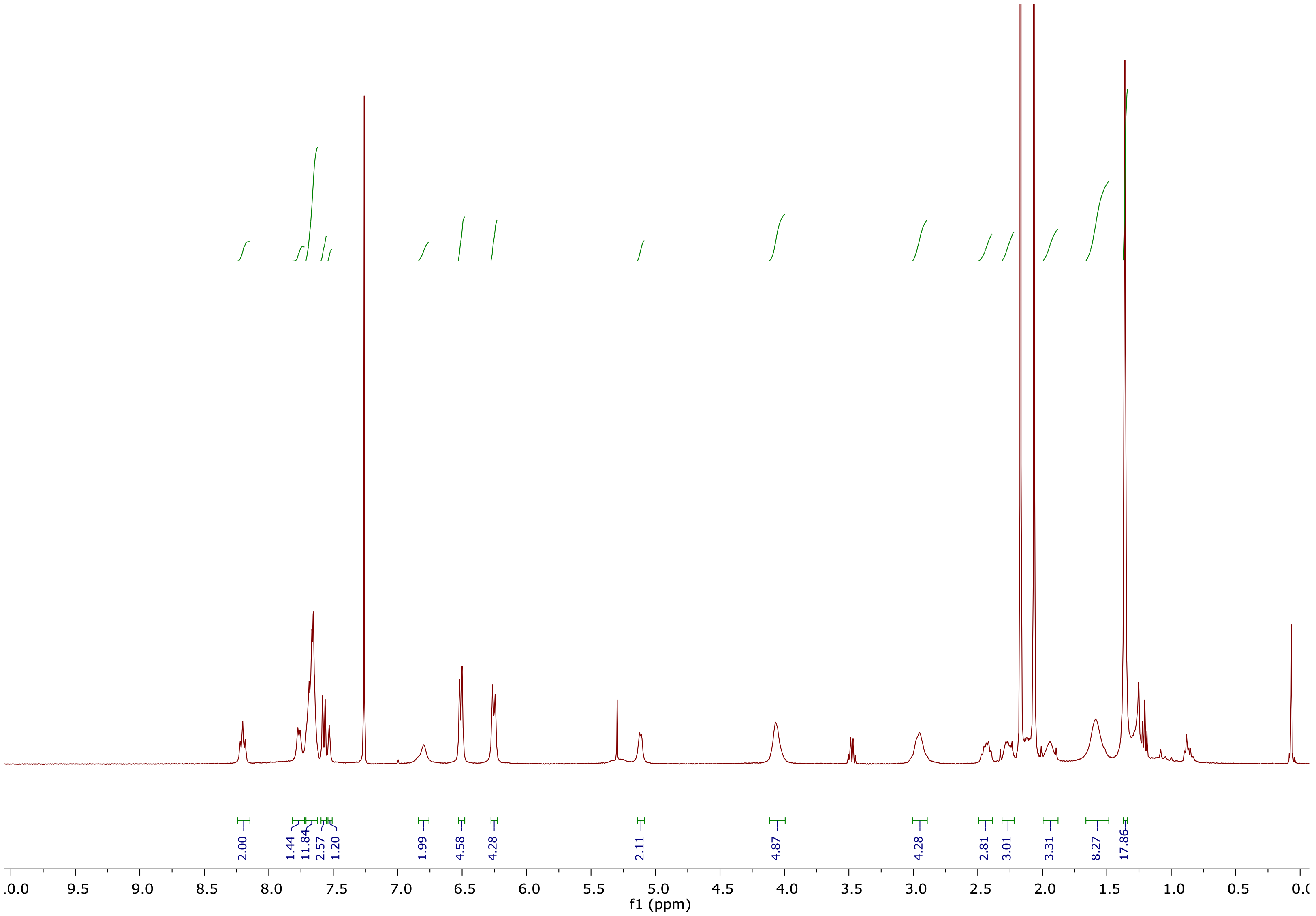

Rotaxane [4AuCu]PF_6SbF_6 ^{31}P NMR ($CDCl_3$, 160 MHz, 300 K)

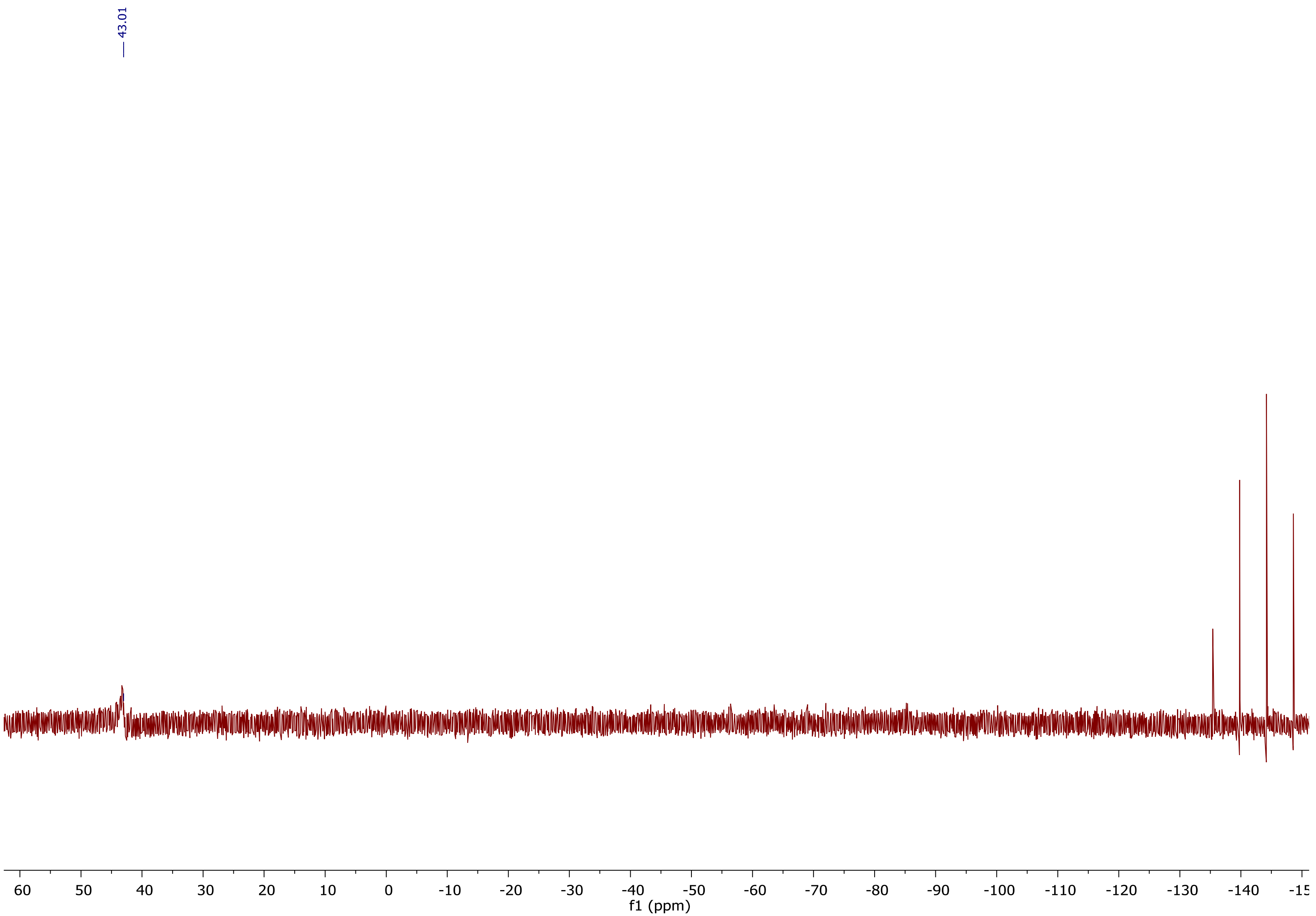

Cyclopropane 10 ^{1}H NMR ($CDCl_3$, 400 MHz, 300 K)

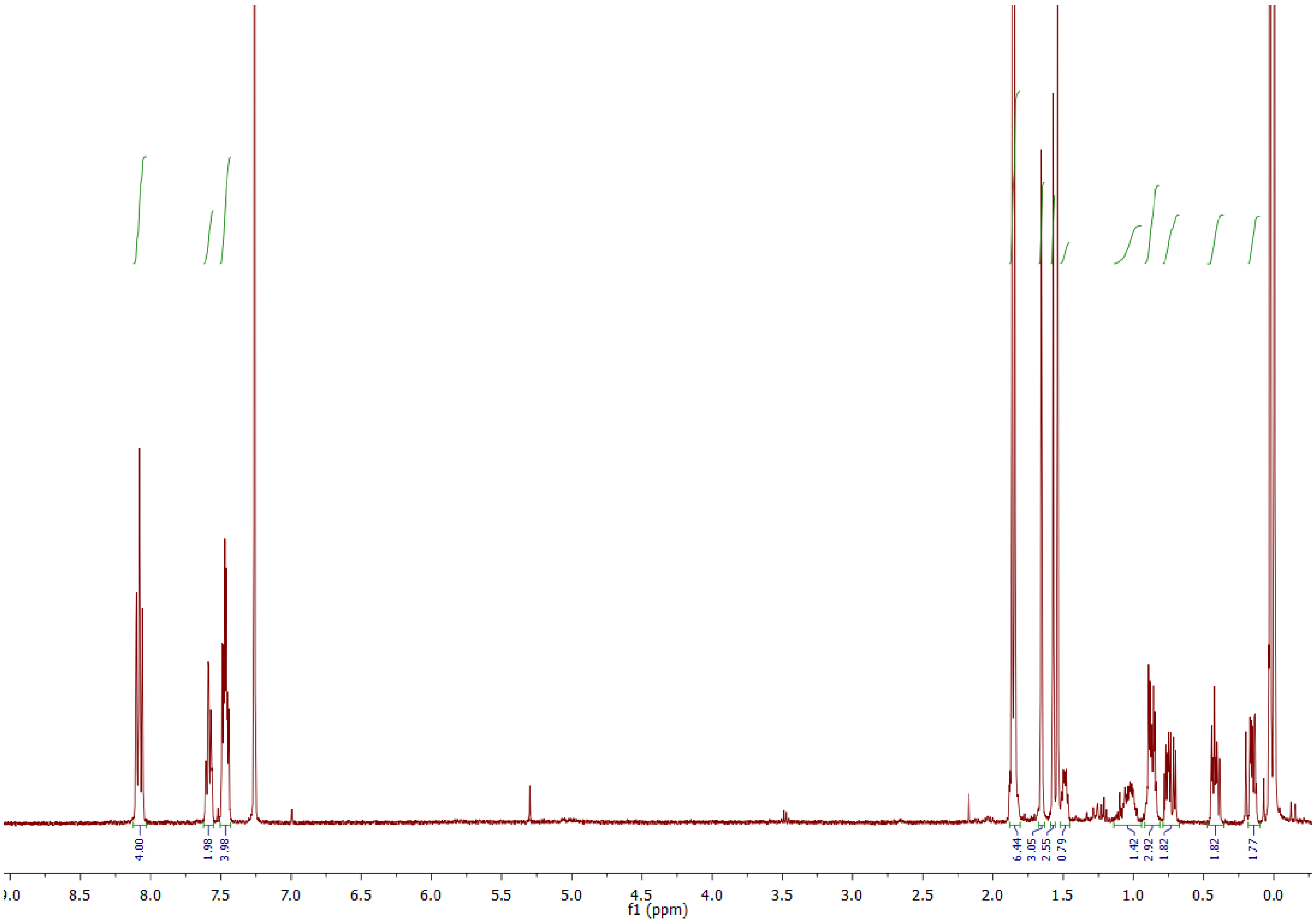

Cyclopropane 10 ^{13}C NMR ($CDCl_3$, 100 MHz, 300 K)

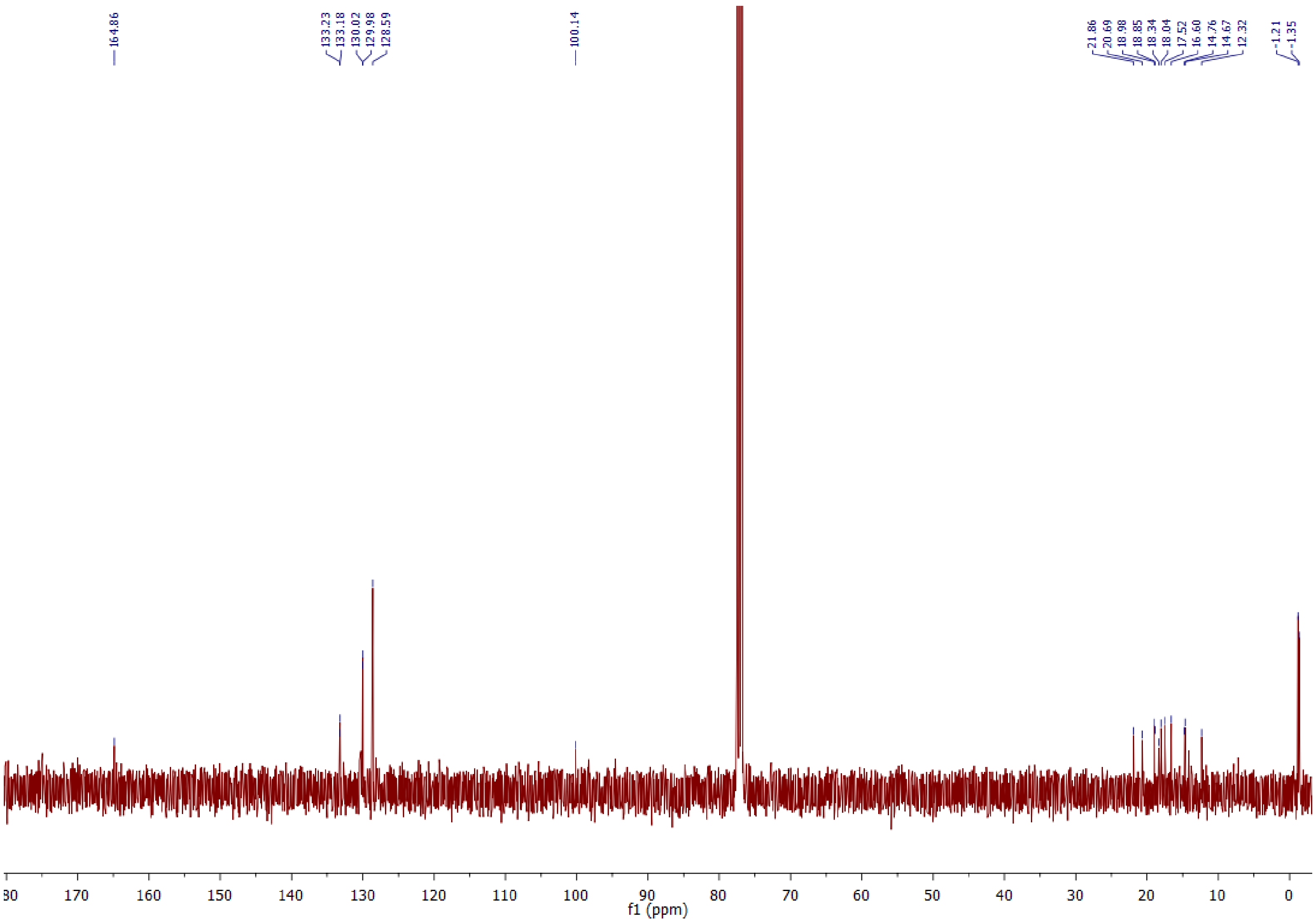

5 References

(1) J. Winn, A. Pinczewska, S. M. Goldup, *J. Am. Chem. Soc.* **2013**, *135*, 13318.

(2) H. Lahlali, K. Jobe, M. Watkinson, S. M. Goldup, *Angew. Chem. Int. Ed.*, **2011,** *50***,** 4151.

(3) E. M. Schuster, M. Botoshansky, M. Gandelman, *Angew. Chem. Int. Ed.* **2008**, *47*, 4555.

(4) M. J. Johansson, D. J. Gorin, S. T. Staben, F. D. Toste, *J. Am. Chem. Soc.* **2005**, *127*, 18002.
